# Synergistic passivation and stepped-dimensional perovskite analogs enable high-efficiency near-infrared light-emitting diodes

**DOI:** 10.1038/s41467-022-35218-0

**Published:** 2022-12-02

**Authors:** Yongjie Liu, Chen Tao, Yu Cao, Liangyan Chen, Shuxin Wang, Pei Li, Cheng Wang, Chenwei Liu, Feihong Ye, Shengyong Hu, Meng Xiao, Zheng Gao, Pengbing Gui, Fang Yao, Kailian Dong, Jiashuai Li, Xuzhi Hu, Hengjiang Cong, Shuangfeng Jia, Ti Wang, Jianbo Wang, Gang Li, Wei Huang, Weijun Ke, Jianpu Wang, Guojia Fang

**Affiliations:** 1 Key Lab of Artificial Micro- and Nano-Structures of Ministry of Education of China, School of Physics and Technology, Wuhan University, Wuhan, China; 2 Key Laboratory of Flexible Electronics (KLOFE) and Institute of Advanced Materials (IAM), Jiangsu National Synergetic Innovation Center for Advanced Materials (SICAM), Nanjing Tech University (Nanjing Tech), Nanjing, China; 3 Institute of Flexible Electronics, Northwestern Polytechnical University (NPU), Xi’an, China; 4 School of Electrical and Electronic Engineering, Wuhan Polytechnic University, Wuhan, China; 5 National Laboratory of Solid-State Microstructures, School of Physics, Nanjing University, Nanjing, China; 6 College of Chemistry & Molecular Sciences, Wuhan University, Wuhan, China; 7 Department of Electronic and Information Engineering, The Hong Kong Polytechnic University, Hong Kong, China

**Keywords:** Organic LEDs, Photonic devices, Electronic devices

## Abstract

Formamidinium lead iodide (FAPbI_3_) perovskites are promising emitters for near-infrared light-emitting diodes. However, their performance is still limited by defect-assisted nonradiative recombination and band offset-induced carrier aggregation at the interface. Herein, we introduce a couple of cadmium salts with acetate or halide anion into the FAPbI_3_ perovskite precursors to synergistically passivate the material defects and optimize the device band structure. Particularly, the perovskite analogs, containing zero-dimensional formamidinium cadmium iodide, one-dimensional δ-FAPbI_3_, two-dimensional FA_2_FA_n-1_Pb_n_I_3n+1_, and three-dimensional α-FAPbI_3_, can be obtained in one pot and play a pivotal and positive role in energy transfer in the formamidinium iodide-rich lead-based perovskite films. As a result, the near-infrared FAPbI_3_-based devices deliver a maximum external quantum efficiency of 24.1% together with substantially improved operational stability. Combining our findings on defect passivation and energy transfer, we also achieve near-infrared light communication with device twins of light emitting and unprecedented self-driven detection.

## Abstract (web summary)

Defect-assisted nonradiative recombination and carrier aggregation at the interface hinder the potential of perovskites as emitter for light-emitting diodes. Here, Fang et al. achieve an external quantum efficiency of 24.1% by combining multidimensional perovskite with cascade conduction bands.

## Introduction

Metal halide perovskites have emerged as one of the most promising light emitters for great applications in lighting and display^1–3^. Specifically, near-infrared perovskite light-emitting diodes (PeLEDs) hold great potential in communication, medical treatment, and detection because of their low-cost fabrication and superior optoelectronic properties, such as narrow half-peak width and extremely high color purity^4^. These essential outstanding properties enable perovskites to be a fierce competitor of other commercial light emitters, such as organic emitters, III–V inorganic semiconductors, and colloidal quantum dots^5^. Owing to excellent perovskite film quality and appropriate device structures, near-infrared PeLEDs have made significant breakthroughs and yielded external-quantum efficiencies (EQE) up to 22.8% in the last few years^1,6–14^.

However, it is a complex energy transfer and conversion process from loading bias to light emission, where two tremendous challenges that need to be addressed simultaneously. One is severe defect-assisted nonradiative recombination in perovskite films. Both positive and negative defects arise spontaneously during the self-assembly nucleation and growth process, resulting in an undesirable increase of nonradiative recombination^7,15–18^. The other one comes from the inefficient carrier transport, which originates from the heterogeneous distribution of energy domains in perovskite films and the band offset between perovskite emitters and carrier injection layers^19–22^. Many efforts have been devoted to introducing either defect passivators or carrier injection enhancers, especially for near-infrared PeLEDs. For instance, Jia et al. introduced Pb(SCN)_2_ into the nano-island structure-based PeLEDs to passivate the defects, thus resulting in the highest radiance to date^23^. Besides, although many previous reports mentioned that the FAPbI_3_ in luminescence is a three-dimensional (3D) structure, and an additional 2-Phenylethanamine hydroiodide (PEAI) can be used to form a two-dimensional (2D) structure to optimize the device performance^24^. Wang et al. incorporated 1-naphthylmethylamine iodide (NMMI) into the perovskite layers to produce quasi-two-dimensional quantum wells and enhance the carrier injection efficiency^25^. Even with so much impressive work to improve the crystal quality or optimize the energy transfer, further enhancement of the near-infrared PeLEDs’ performance is still limited by the single function of these additives. Therefore, it is desirable to have superior strategies that can solve both two essential problems simultaneously and assemble an ideal device band structure for carrier injection to minimize energy losses.

Herein, we introduced a series of multifunctional cadmium salt additives with acetate or halide anion to form efficient FA-based perovskite emitters, which contained stepped-dimensional perovskite analogs and could solve both serious defect-induced nonradiative recombination and inefficient carrier injection challenges. The best representative cadmium acetate (CdAc_2_) with different charge functional groups was used to analyze the effect of these cadmium salts on perovskites. Lewis-acid functional cadmium cation (Cd^2+^) and Lewis-alkali functional group C=O in CdAc_2_ have a good affinity to bind with the lead and iodine vacancies on the perovskite crystal surface, respectively^7,26–30^. Such synergistic cation-anion passivation effects of CdAc_2_ were proved by the density functional theory (DFT) calculations and our experimental results. More interestingly, the introduction of Cd^2+^ led to a 4.17 eV-bandgap zero-dimensional (0D) perovskitoid of FA_2_CdI_4_ into the FAPbI_3_ light-emission layers, which was confirmed by single-crystal X-ray crystallography analyses. In addition to 0D FA_2_CdI_4_, we also found that there are one-dimensional (1D) yellow-phase (δ-phase) FAPbI_3_ and 2D FA_2_FA_n−1_Pb_n_I_3n+1_ in our FAPbI_3_ films. Although 1D δ-phase (a yellow and non-perovskite phase) FAPbI_3_ with a wide bandgap is usually very harmful to the devices^31^, we found that 1D δ-FAPbI_3_ can play a pivotal and positive role in charge injection for our devices. And different from traditional 2D perovskites with a formula of (A’)_2_(A)_n−1_B_n_X_3n+1_ requiring one bulk organic molecule as an A’-site cation and the other small molecule as an A-site cation^32–34^, our 2D (FA)_2_(FA)_n-1_Pb_n_I_3n+1_ perovskites contained FA^+^ as both A’ and A-site cation by using excess formamidinium iodide (FAI) in perovskite precursors. Consequently, the stepped-dimensional perovskite analogs (from 0D FA_2_CdI_4_ to 1D δ-FAPbI_3_, 2D FA_2_FA_n-1_Pb_n_I_3n+1_, and 3D α-FAPbI_3_) gave a cascade band structure and could minimize energy losses of PeLEDs, which benefit from both effective defect passivation and energy-transfer optimization comparing with traditional 3D perovskite emitters. Therefore, the resultant near-infrared PeLEDs with CdAc_2_ additive and excess FAI yielded a significantly improved average EQE of 22.4 ± 1.7% with a record value of 24.1% at 804 nm. With the aid of a polydimethylsiloxane (PDMS) organic glass hemisphere, we successfully enhanced the device out-coupling efficiency and further pushed the EQE to 29%. In addition to PeLEDs, our devices exhibited very short electrical and light-response time, enabling a practical NIR-communication system composed of device twins, in which one for light emitting and the other for self-driven detection.

## Results and discussion

### Synergistic passivation of the representative CdAc_2_ additive

**Fig. 1:**
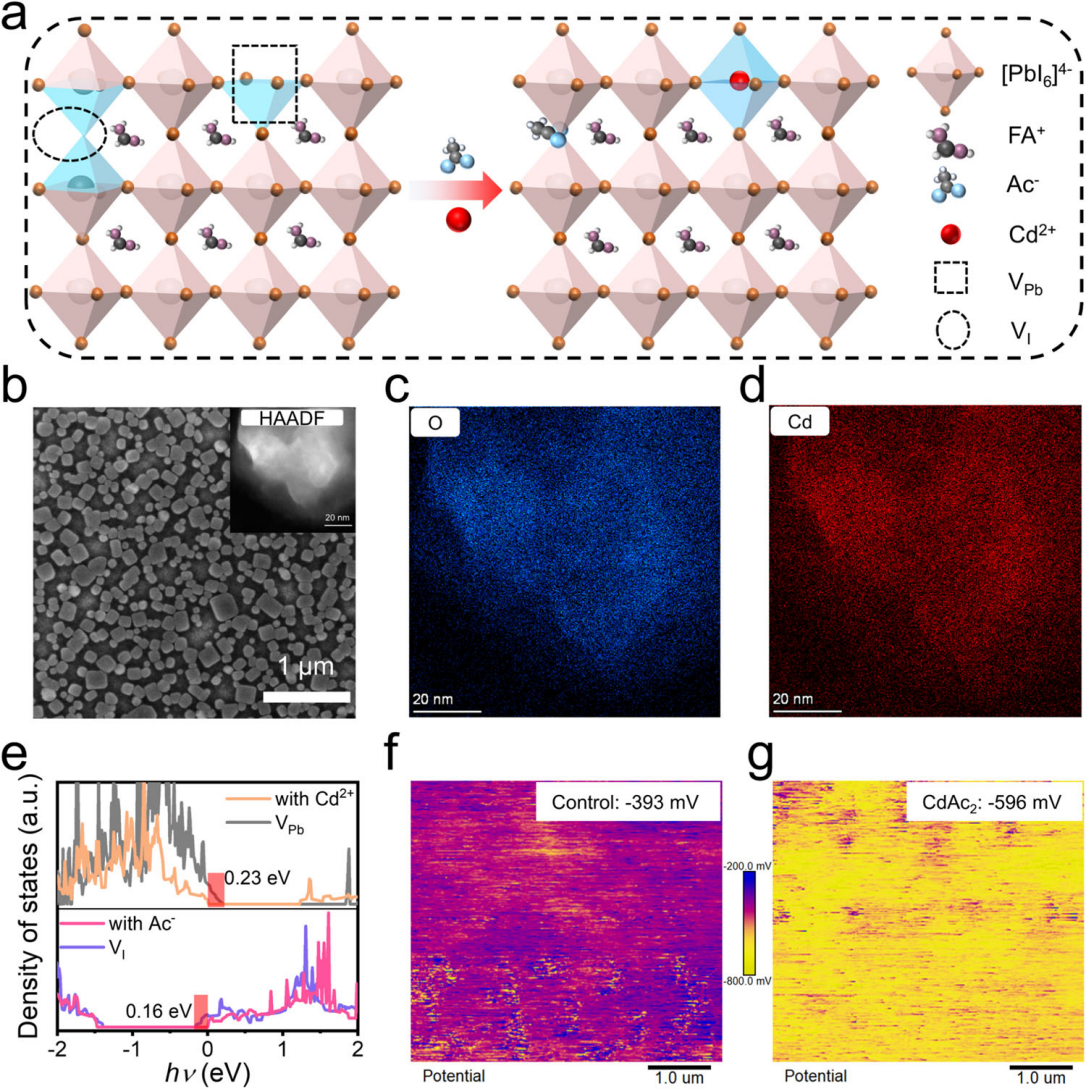
Effects of CdAc_2_ on perovskite grains and films. **a** Schematic diagram of defect passivation on the FAPbI_3_ perovskite grain surface by CdAc_2_. **b** Scanning electron microscope (SEM) image of a FAPbI_3_ perovskite film with 2.5% CdAc_2_, the scale bar for the image is 1 μm. Inset: a HAADF-STEM image of a perovskite grain extracted from the CdAc_2_-added FAPbI_3_ without 5AVA. Energy-dispersive spectrum (EDS) mapping images of **c** O and **d** Cd, the scale bars for these images are 20 nm. **e** Density of states (DOS) of an un-passivated and passivated surface with Cd^2+^ or Ac^−^. Kelvin probe force microscopy (KPFM) images of the films **f** without and **g** with CdAc_2_.

Defect-induced nonradiative recombination is one of the most important factors affecting the performance of perovskite devices. The introduction of the representative CdAc_2_ additive could first passivate the relevant defects. A schematic of two surface defects on FAPbI_3_-based perovskites, negatively charged defects caused by lead vacancies (V_Pb_) and positively charged defects caused by iodine vacancies (V_I_), is shown in Fig. 1a. We used CdAc_2_ as a cation-anion passivator because the intrinsic defects on the FAPbI_3_ grain surface can serve as nonradiative recombination centers and lead to poor performance and serious ion migration^35^. In general, lead and halogen vacancies are ubiquitous at the surface of solution-processed perovskite grains^15,36,37^, which can easily trap excitons and cause nonradiative recombination. For comparison, we precisely introduced and investigated the influences of different passivation molecules (PMs: cadmium iodide (CdI_2_), or lead acetate (PbAc_2_), CdAc_2_) on solution-processed FAPbI_3_ perovskite emitters. Briefly, the perovskite precursors were prepared by dissolving FAI, lead iodide (PbI_2_), 5-aminovaleric acid (5AVA), and PMs with a molar ratio of 2/1/0.85/*x* (*x* = 0~0.2). Excess FAI was used to promote the formation of α-phase FAPbI_3_, as demonstrated in literature^23,31^. The 5AVA could provide initial passivation and form a dispersed island-like structure that would increase the light out-coupling efficiency in perovskite films^1^.

We observed a visual color change in PbI_2_ solution with CdI_2_, CdAc_2_, and formamidinium acetate (FAAc), implying a strong interaction between Cd^2+^ and Ac^−^ with PbI_2_ components (Supplementary Figure 1). Figure 1a shows the possible passivation model of how CdAc_2_ passivated the two types of defects on the periphery of perovskite crystals. On one hand, the positively charged Cd^2+^ can effectively interact with the negatively charged I^−^ around the V_Pb_ on the grain surface. On the other hand, the lone pair of electrons in Ac^−^ can combine with the uncoordinated lead, thus passivating the positively charged defects caused by V_I_^29,38^. Therefore, the simultaneous introduction of Ac^−^ and Cd^2+^ provides a possible strategy for synergistic passivation.

To severally investigate the effects of Cd^2+^ and Ac^−^ ions on the optical and electrical properties of the perovskite films, we prepared a series of perovskite films with different PMs. Perovskite films without PMs were also fabricated as a reference. The added PMs contained different functional cations and anions such as separate Cd^2+^ (CdI_2_), separate Ac^−^ (PbAc_2_), and both of them (CdAc_2_). Figure 1b presents a scanning electron microscope (SEM) image of a representative CdAc_2_-added film and Supplementary Fig. 2 also provides SEM images of the initial perovskite film and the films with CdI_2_, PbAc_2_, and CdAc_2_ additives for comparison. We can see that all these samples showed a nano-island structure, but their morphology owned a slight difference. The grains of the CdAc_2_-added films exhibited more cubic-shape characteristics rather than obvious grain enlargement as literature reported^39^. We supposed that the Cd^2+^ and Ac^-^ were distributed uniformly on the surface of individual grains, which were verified by high-angle annular dark-field scanning transmission electron microscopy (HAADF-STEM) and energy-dispersive X-ray spectroscopy (EDS) measurements (Fig. 1b–d, Supplementary Fig. 3).

To illustrate the interaction of Cd^2+^ and Ac^−^ on perovskites, X-ray photoelectron spectroscopy (XPS) measurements were conducted and the relevant results are given in Supplementary Figure 4. Note that all PMs-added samples were obtained using the optimal addition amounts (that is, CdI_2_:10%, PbAc_2_:5%, and CdAc_2_:2.5%), unless particularly stated. For the films with different PMs, the binding energies of I 3*d* and Pb 4*f* shifted in different directions, which result from the overlaps of the electron clouds between Cd, O in Ac^−^, and the other elements on the surrounding lattice. Cd is much less attractive to electrons in the outer layer than other elements, and O shows the maximum attractive ability^38,40^. And when both Ac^−^ and Cd^2+^ were added to the perovskite films, the binding energies of all related elements became slightly larger compared with the control group, which means that the whole crystal exhibited a more stable structural feature^41^. Note that after introducing Cd^2+^ (CdI_2_ and CdAc_2_ groups), two extra peaks were observed at 412.96 eV and 405.5 eV, corresponding to Cd 3*d*_5/2_ and Cd 3*d*_3/2_, respectively. This indicated the successful bonding of Cd ions with perovskite components. Fourier Transform Infrared (FTIR) spectroscopy measurements were then performed to further confirm the interaction between Ac^-^, Cd^2+^, and perovskite (Supplementary Fig. 5). Notably, the C=O vibration peak at 1688 cm^−1^ in the mixture of FAAc and PbI_2_ shifted towards the direction of that in PbAc_2_, which strongly demonstrates the interaction between Pb^2+^ and Ac^-^ group^38^. The addition of both PbAc_2_ and CdAc_2_ can change the stretching and bending vibrations of the -NH_2_ group, suggesting a potential strong hydrogen bonding-like interaction between the Ac^-^ and perovskite lattice, which may result in dual-passivation effects. Ultraviolet-visible (UV–Vis) absorption and photoluminescence (PL) analyses were then conducted to investigate the optical characteristics of the perovskite films with different PMs (Supplementary Fig. 6). The differences in film morphology and crystallinity caused variations in absorption intensity but not the onset of the film absorption, implying that the addition of PMs did not significantly alter the perovskite optical bandgap. We then calculated the Urbach energy (*E*_u_) of the perovskite films with different PMs through their corresponding absorption curves. The introduction of CdAc_2_ effectively reduced the *E*_u_ to 126 meV, in comparison with the *E*_u_ of 140 meV for the control sample. Such a lowered *E*_u_ usually indicates a reduced number of band-edge trap states^10,42^. On the basis of these results, we can conclude that the perovskite grains can be effectively passivated and stabilized by both Cd^2+^ and Ac^-^ groups, resulting from the strong interaction between the PMs and PbI_6_ octahedrons.

To investigate the extent of passivating effects of CdAc_2_ on perovskite films, time-resolved photoluminescence (TRPL) and photoluminescence quantum efficiency (PLQE) measurements were carried out and the corresponding results are presented in Supplementary Fig. 7. By fitting the TRPL spectra with a biexponential function, two types of carrier decay time constants, i. e. the lifetimes of the fast and slow decay components, and average carrier lifetimes (*τ*_av_) can be obtained. Radiative and nonradiative recombination rates (*k*_rad_ and *k*_non-rad_, respectively) of the samples can be calculated by combining the obtained *τ*_av_ and PLQE values. Supplementary Table 1 lists the detailed paraments. For the perovskite films with CdAc_2_ passivation, there exhibited a long *τ*_av_ of 2.1 μs and a high PLQE of 79% at an excitation intensity of 20 mW·cm^-2^, as well as an increased radiation recombination composition. Although passivation of halogen vacancies (i. e. V_I_) has less impact on lifetime than that of deep-level defects such as V_Pb_, the anchoring of halogen vacancy defects by CdAc_2_ makes it more difficult to convert to other deep-level defects, thereby increasing the carrier lifetime. Therefore, we can conclude that the quality of perovskite films was significantly improved with the aid of the synergistic passivation effects of Cd^2+^ and Ac^−^.

The density of states (DOS) of the perovskites with V_Pb_ and V_I_ defects was calculated using DFT to explain the synergistic effects of the CdAc_2_. Briefly, the Cd^2+^ cations can effectively passivate the defects caused by V_Pb_, which introduced a defect level (0.23 eV above the valence band maximum) and serves as hole traps (Fig. 1e)^43^. Meanwhile, the Ac^-^ anions suppress V_I_ and combine with the unsaturated lead on the grains’ surface, where V_I_ introduced another defect level (0.16 eV below the conduction band minimum) in the bandgap and served as electron traps (Fig. 1e). Therefore, CdAc_2_ successfully formed cation-anion passivation effects on perovskite films (Supplementary Figs. 8 and 9). Space-charge-limited current (SCLC) measurements were then used to estimate the trap densities of perovskite films with and without CdAc_2_ (Supplementary Fig. 10). Since the near-infrared light emitters can only be deposited on zinc oxide (ZnO) substrates, we fabricated single-electron devices to estimate the trap density with the structure of indium tin oxide (ITO)/ZnO/poly-ethylenimine ethoxylated (PEIE)/Perovskite/1,3,5-tris(1-phenyl-1H-benzimidazole-2-yl)benzene (TPBi)/LiF/Al. For the device with CdAc_2_ incorporation, the density of electron traps decreased from around 9.5 × 10^17^ cm^−3^ to 3.6 × 10^17^ cm^−3^, which is about one-third of the control one. Such results verified the passivation effects of CdAc_2_ on perovskite films.

KPFM measurements were then conducted to verify the effect of CdAc_2_ on the electrical properties of the perovskite films. As shown in Fig. 1f, g, the surface potential of the CdAc_2_-added film was about 200 mV higher than that of the control sample. This result can be confirmed by the following ultraviolet photoelectron spectroscopy (UPS) measurements, which were employed to get an insight into the possible influences of CdAc_2_ on the energy band structure of the perovskite films. The results shown in Supplementary Figs. 11 and 12 indicate that the Fermi energy (*E*_f_) of the CdAc_2_-added perovskite film raised from −4.94 eV of the control one to −4.72 eV. Accordingly, the conduction band minimum (*E*_CBM_) also shifted from −4.20 eV to −4.05 eV, which facilitated the injection of electron carriers from ZnO to perovskite emitters. The KPFM results of the sample with CdAc_2_ post-treatment (Supplementary Fig. 13) also demonstrated the effect of ultra-thin CdAc_2_ on the conductivity of perovskite layers. The schematic diagram of the energy level change of perovskite is given in Supplementary Fig. 14, and the uplift Fermi level and conduction band imply a smoother electron injection from ZnO into the perovskite emission layer.

### Low-dimensional materials in 3D FAPbI_3_ light emitters

**Fig. 2:**
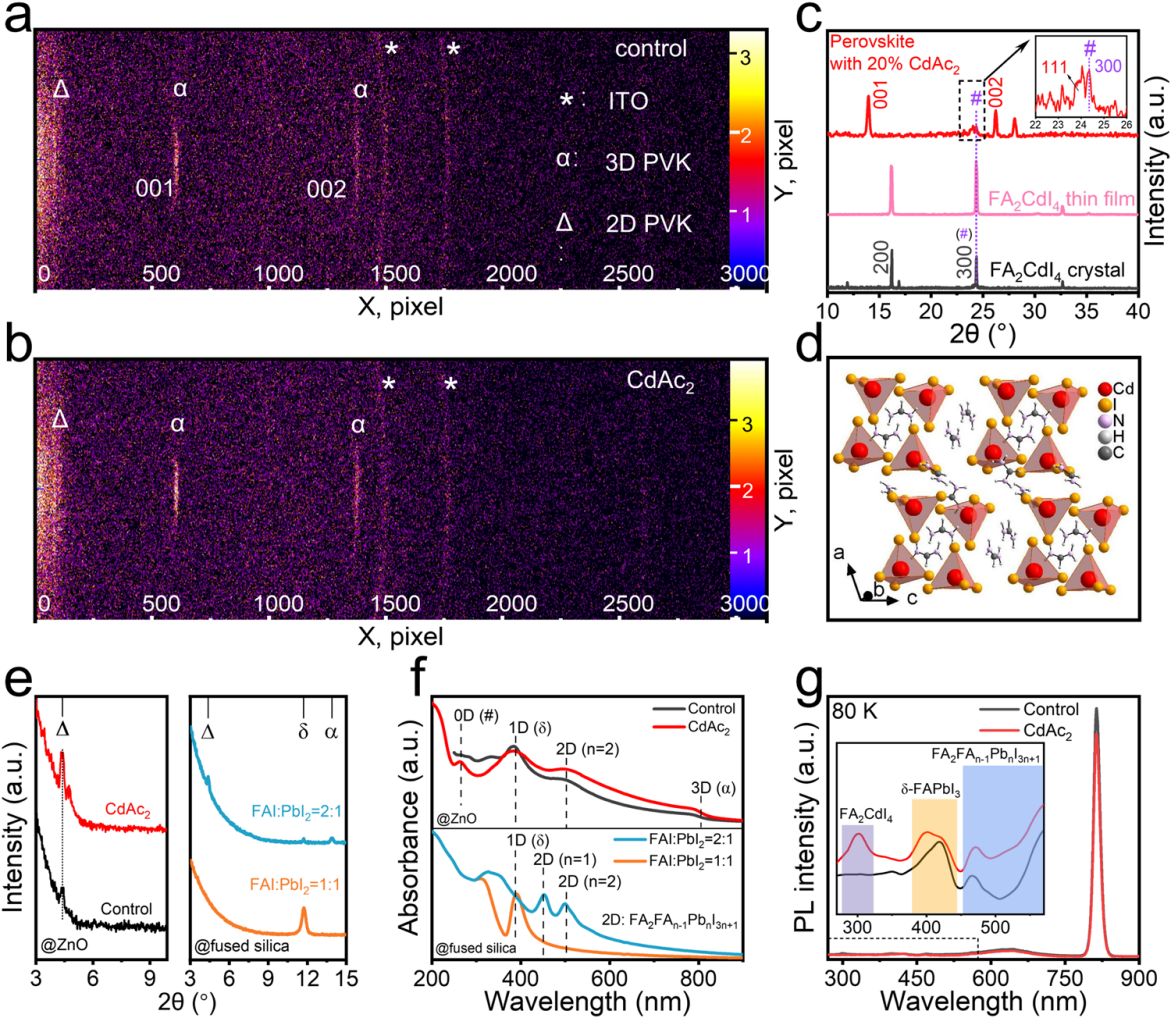
stepped-dimensional materials in FAPbI_3_ perovskites films. 2D-XRD patterns of the perovskite films **a** without and **b** with CdAc_2_ additive. **c** 1D-XRD patterns of the FA_2_CdI_4_ crystal, FA_2_CdI_4_ thin film, and perovskite film with 20% CdAc_2_. **d** Crystal structure of 0D FA_2_CdI_4_ perovskitoid. **e** The left plot:1D-XRD patterns of the films without and with CdAc_2_ deposited on ZnO/fused silica substrates. The right plot: 1D-XRD patterns of the films with the different molar ratios of FAI and PbI_2_ deposited on fused silica substrates. **f** The up plot: UV–vis absorption spectra of the films without and with CdAc_2_ deposited on ZnO/fused silica substrates. The down plot: UV–vis absorption spectra of the films with the different molar ratios of FAI and PbI_2_ deposited on fused silica substrates. All the films on fused silica substrates were free of 5AVA. **g** Low-temperature steady-state photoluminescence spectra of the perovskite films with and without CdAc_2_ coated on fused silica substrates. The marks of *, #, α, Δ and δ indicate the presence of ITO, 0D FA_2_CdI_4_ perovskitoid, 3D α-phase FAPbI_3_ perovskite, 2D FA_2_FA_n−1_Pb_n_I_3n+1_ perovskites, and δ-phase FAPbI_3_, respectively.

Figure 2a, b presents 2D X-ray diffraction (2D-XRD) maps of the perovskite thin films without and with CdAc_2_ additive, respectively. As compared with the neat CdAc_2_-free film, the shorter arc length in the longitudinal direction and the narrower width in the transverse direction, as well as the slightly brighter signal were observed from the CdAc_2_-added perovskite film, indicating the validity effect of CdAc_2_ in improving the crystal quality of perovskite grains. Two strong diffraction peaks appeared at 13.89° and 28.00° without any shift, which corresponds to the (001) and (002) crystal planes of α-phase FAPbI_3_ (Supplementary Fig. 15), respectively. The above results suggest that Ac^−^ and Cd^2+^ ions were not able to enter the perovskite lattice, but can obviously influent the film crystallinity. When the addition ratio of CdAc_2_ increased to 20%, two additional characteristic peaks appeared at 24.18° and 24.38° (marked as #), shown in Fig. 2c. The former belongs to a (111) plane of FAPbI_3_ and the latter does not belong to FAPbI_3_ perovskites.

Given the presence of excess FAI in the precursor solution, we speculated that the peak at 24.38° corresponds to a new material of 0D FA_2_CdI_4_ perovskitoid (Supplementary Fig. 16a–d, Supplementary Data 1) that only arises from excess FAI and Cd^2+^ sources in the precursors. The same structural features can be observed in stoichiometric FA_2_CdI_4_ crystals and polycrystalline films. Single-crystal X-ray crystallography analyses were used to confirm the crystal structure of the 0D FA_2_CdI_4_ and the relevant results were given in Supplementary Table 2. The CdI_4_ tetrahedron consisted of a central cadmium atom and four surrounding iodine atoms. Moreover, there are no shared atoms among these tetrahedrons in the crystal, because the formamidinium cation completely separates the individual CdI_4_ tetrahedra, finally forming a 0D FA_2_CdI_4_ perovskitoid. The 0D structural features of the crystal are presented in Fig. 2d. To further verify the universality of our approach, we also introduced several other cadmium salts such as CdBr_2_ and CdCl_2_ as additives and observed the same diffraction peaks at 24.38° for the perovskite films added with these two cadmium salts (Supplementary Fig. 16e). This further proves that this type of 0D material was produced with the addition of Cd^2+^. The slightly blue-shift emission peaks result from the changed bandgap by the introduction of Br or Cl halogen (Supplementary Fig. 16f).

Apart from 0D FA_2_CdI_4_, we also found some other low-dimensional impurity phases in our FAPbI_3_ light-emission layers. FAPbI_3_ films coated on ZnO-based substrates with low-temperature annealing (~105 °C) typically contain multiple components rather than a single α-phase, as demonstrated in previous reports^31,44^. Low-temperature annealing (<150 °C) is a key reason for the formation of the δ-phase, which can be suppressed by the deprotonation of ZnO but does not disappear completely in the films. Moreover, regardless of whether there was CdAc_2_ in the films, we observed an extra diffraction peak at 4.37°, which can be only assigned to 2D *n* = 1 FA_2_PbI_4_ perovskites (Fig. 2e, the left plot)^45,46^. The formation of two-dimensional (2D) FA_2_FA_n−1_Pb_n_I_3n+1_ perovskites is highly related to the content of FAI in the precursor solution^45,46^. The 2D FA_2_FA_n−1_Pb_n_I_3n+1_ perovskites include FA as both A′-site and A-site cations and are different from the traditional 2D perovskites with a formula of (A′)_2_(A)_n−1_B_n_X_3n+1_ requiring one bulk organic molecule as an A′-site cation and the other small molecule as an A-site cation^46^. We also deposited perovskite films on pure fused silica substrates to take a closer look at the composition of the films. When the precursors were following a stoichiometric ratio (105 °C annealing), the film presented a single δ-phase. When the FAI was super-excessive, complex phases composed of 1D δ-phase FAPbI_3_, 2D FA_2_FA_n−1_Pb_n_I_3n+1_ perovskites, and 3D α-phase FAPbI_3_ perovskites were present in the films (Fig. 2e, the right plot). The addition of excess FAI and ZnO can promote the formation of α-phase FAPbI_3_ but inevitably produce low-dimensional phases. Under the premise of ZnO, δ-phase perovskites were greatly suppressed, and α-phase FAPbI_3_ perovskites were formed when the FAI was sufficient^31^. However, the deprotonation reaction of ZnO can greatly inhibit the formation of the δ phase but not completely eliminate it, which is consistent with the obvious exciton absorption peaks in Fig. 2f. The exciton absorption signal at around 500 nm indicates the existence of other phases besides the δ phase. Perovskites with different ratios of FAI and PbI_2_ on fused silica substrates clearly showed the presence of low-dimensional materials (Supplementary Figure 17). When the ratio of FAI to PbI_2_ in the precursor was 1:1, the thin film exhibited strong exciton absorption at around 400 nm, which is related to 1D δ-phase FAPbI_3_ perovskite^46,47^. And when the ratio became 2:1, the other two exciton absorption peaks appeared at around 460 nm and 500 nm, which correspond to *n* = 1 and 2 phases of 2D FA_2_FA_n−1_Pb_n_I_3n+1_, respectively^46^. The perovskite films deposited on ZnO/fused silica substrates still exhibited both 1D and 2D exciton absorption characteristics with or without the addition of CdAc_2_ (Fig. 2f). But the difference is that the perovskites with CdAc_2_ additive showed an additional exciton absorption at around 280-290 nm, which is related to the 0D FA_2_CdI_4_ perovskitoid. Low-temperature steady-state photoluminescence spectra were used to demonstrate that δ-phase FAPbI_3_ as well as 2D FA_2_FA_n−1_Pb_n_I_3n+1_ did exist in perovskite films (Fig. 2g). Briefly, the PL spectra exhibited dominant emission peaks at 814 nm for both films with or without CdAc_2_. Additionally, we found obvious emissions from the δ-phase at around 400 nm and *n* = 1 FA_2_FA_n−1_Pb_n_I_3n+1_ phase at around 470 nm in our as-fabricated perovskite films. Other broad signals rising from 500 nm were the excitation of the 2D FA_2_FA_n-1_Pb_n_I_3n+1_ (*n* > 1) phases. The signal at around 300 nm in the CdAc_2_-added perovskite film could only be attributed to the excited states of the 0D FA_2_CdI_4_ perovskitoid, verifying that the addition of CdAc_2_ did lead to the formation of the 0D FA_2_CdI_4_ perovskitoid. These findings prove that it is inevitable to form δ-phase perovskites under annealing at 105 °C, and also form 2D FA_2_FA_n-1_Pb_n_I_3n+1_ perovskite phases in the case of excess FAI. Although the deprotonation reaction of ZnO can inhibit the formation of these two phases, it cannot completely disappear. Moreover, both components are excitable in perovskite films, implying that there may be a complex energy transfer pathway in the films instead of just the excitation of 3D α-FAPbI_3_ perovskite materials.

### Energy transfer and carrier transport among low-dimensional materials and 3D FAPbI_3_

**Fig. 3:**
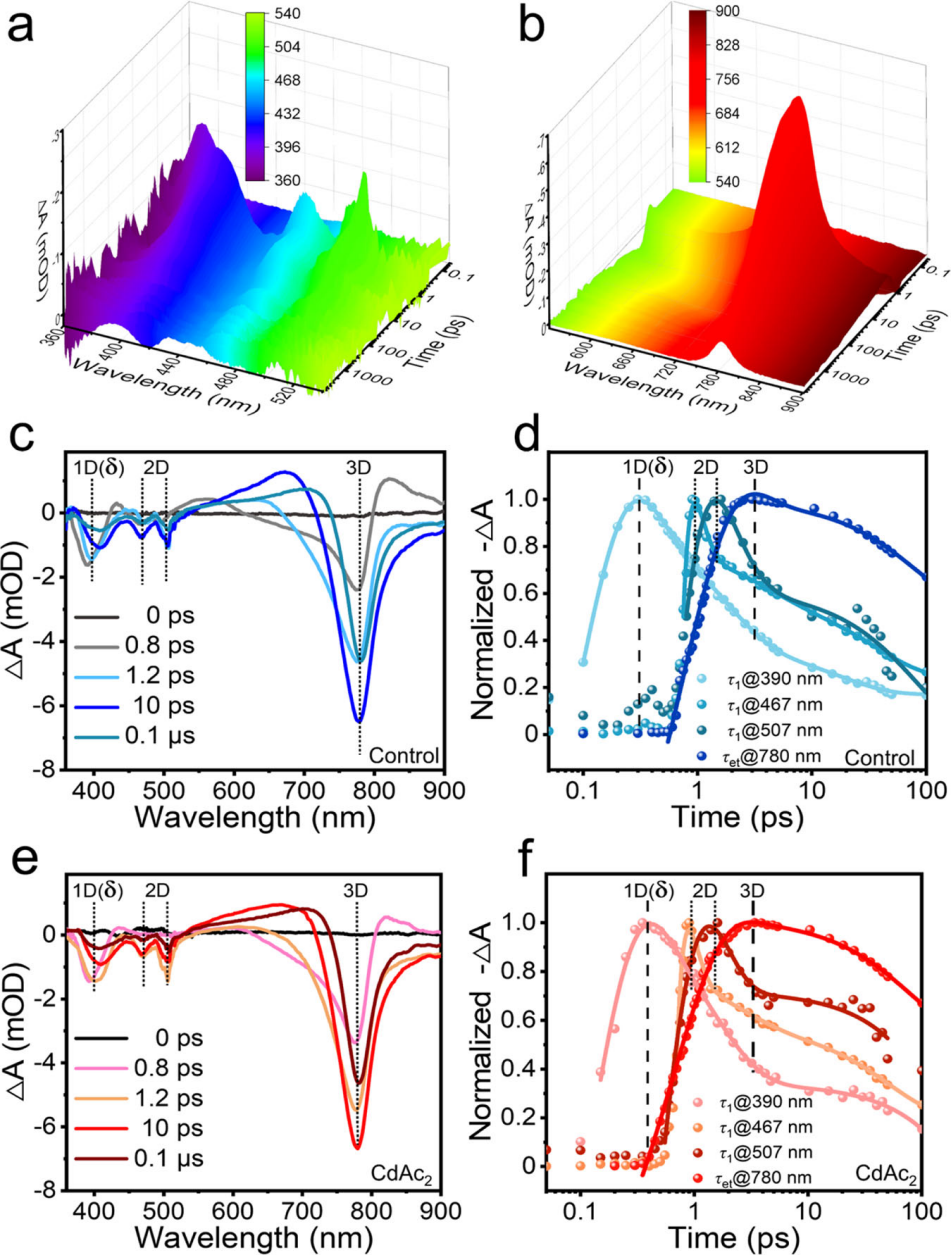
Energy transfer and recombination dynamics for perovskite films and devices. The original TA spectra located at **a** 360–540 nm and **b** 540–900 nm for a pristine FAPbI_3_ perovskite film. TA spectra at selected timescales of the FAPbI_3_ perovskite films **c** without and **e** with CdAc_2_ additive. TA spectra at different wavelengths as a function of delay times of the FAPbI_3_ films **d** without and **f** with CdAc_2_ additive.

To study the energy transfer and recombination dynamics of photogenerated carriers in perovskite films, transient absorption (TA) measurements were conducted. As confirmed by TA spectra, we can also find four distinct ground-state-bleach (GSB) peaks appeared near 390, 467, 507, and 780 nm in the pristine perovskite films (Fig. 3a, b), which are related to the above-mentioned 1D δ-FAPbI_3_, 2D FA_2_PbI_4_ (*n* = 1), 2D FA_3_Pb_2_I_7_ (*n* = 2), 3D α-FAPbI_3_ (GSB_α_)^48^, respectively. We did not observe the signal of 0D 4.17 eV-bandgap FA_2_CdI_4_ because of the limitations of our instrument where a 365 nm laser was used. Clearly that the bleaching signal initially appeared at around 390 nm and soon two signals emerged at 467 nm and 507 nm (Fig. 3c), indicating the carrier transport from 1D δ-FAPbI_3_ to 2D FA_2_PbI_4_ (*n* = 1) and then 2D FA_3_Pb_2_I_7_ (*n* = 2). And when these three signals began to decay, the GSB_α_ continued to increase, indicating the arrival of the carriers in 3D α-FAPbI_3_. The decay kinetics of the four GSB peaks in the pristine film exhibited in Fig. 3d provides a more distinct characterization of this process. The ultrafast decay for the bleaching at 390, 467, and 507 nm is attributed to the energy transfer process according to the literature^19,21^. And this process contains three time constants of *τ*_1,1_ (242fs), *τ*_1,2_ (173fs) and *τ*_1,3_ (647fs), respectively. The slow decay for the GSB_α_ at 780 nm is related to the charge trapping as reported^25,49^. And the fitted rising time (*τ*_et_) for GSB_α_ is 952fs, which is in good agreement with the sum of three fast decay time constants, coincidentally. For comparison, the decay kinetics characterizations of the CdAc_2_-added film suggest that the time constants (*τ*_1,1_, *τ*_1,2_, *τ*_1,3_) became much shorter than that in the pristine film (Fig. 3e, f, Supplementary Table 3) and the fitted rising time reduced by about one fifth to 769 fs, confirming the positive effects of CdAc_2_ in carrier transport. All the longest delays occurred at 780 nm, the narrowest bandgap, indicating that the excited carriers flowed into the emitting domains along the direction of the stepped-dimensional materials with decreasing bandgaps, just like an energy transfer funnel in conventional quasi-2D green perovskite LEDs^10^.

We considered that these enhanced energy transfer effects came not only from the effective defect passivation of CdAc_2_, but also from the energy transfer ladder of 0D FA_2_CdI_4_. Specifically, the *E*_CBM_ of the FA_2_CdI_4_ phase is located in the middle of ZnO and Pb-based low-dimensional phases (Supplementary Fig. 18), thus acting as a ladder for energy transfer to reduce the potential barrier for carrier injection. The energy level of 1D δ-phase FAPbI_3_ was also given in Supplementary Fig. 19, the flat conduction band and slightly lower Fermi level facilitate electron carrier injection compared to 0D FA_2_CdI_4_. Given the proper energy band levels inserted between ZnO and 3D FAPbI_3_ perovskites, the decay kinetics of the GSB peaks matched very well with the energy funnel model. The whole energy transfer process could finish just in the order of femtoseconds with minimized energy losses and would ultimately bring out the high electroluminescent performance of PeLEDs. We estimated the proportions of different dimensional components in the perovskite films by integrating the TA spectra and the XRD patterns of the perovskite films. The results in Supplementary Fig. 20 showed that the ratio of multidimensional perovskites in our optimized CdAc_2_-added perovskite film was around 3/10/10/100 (0D/1D/2D/3D).

### Device performance of PeLEDs and self-driven detection system

**Fig. 4:**
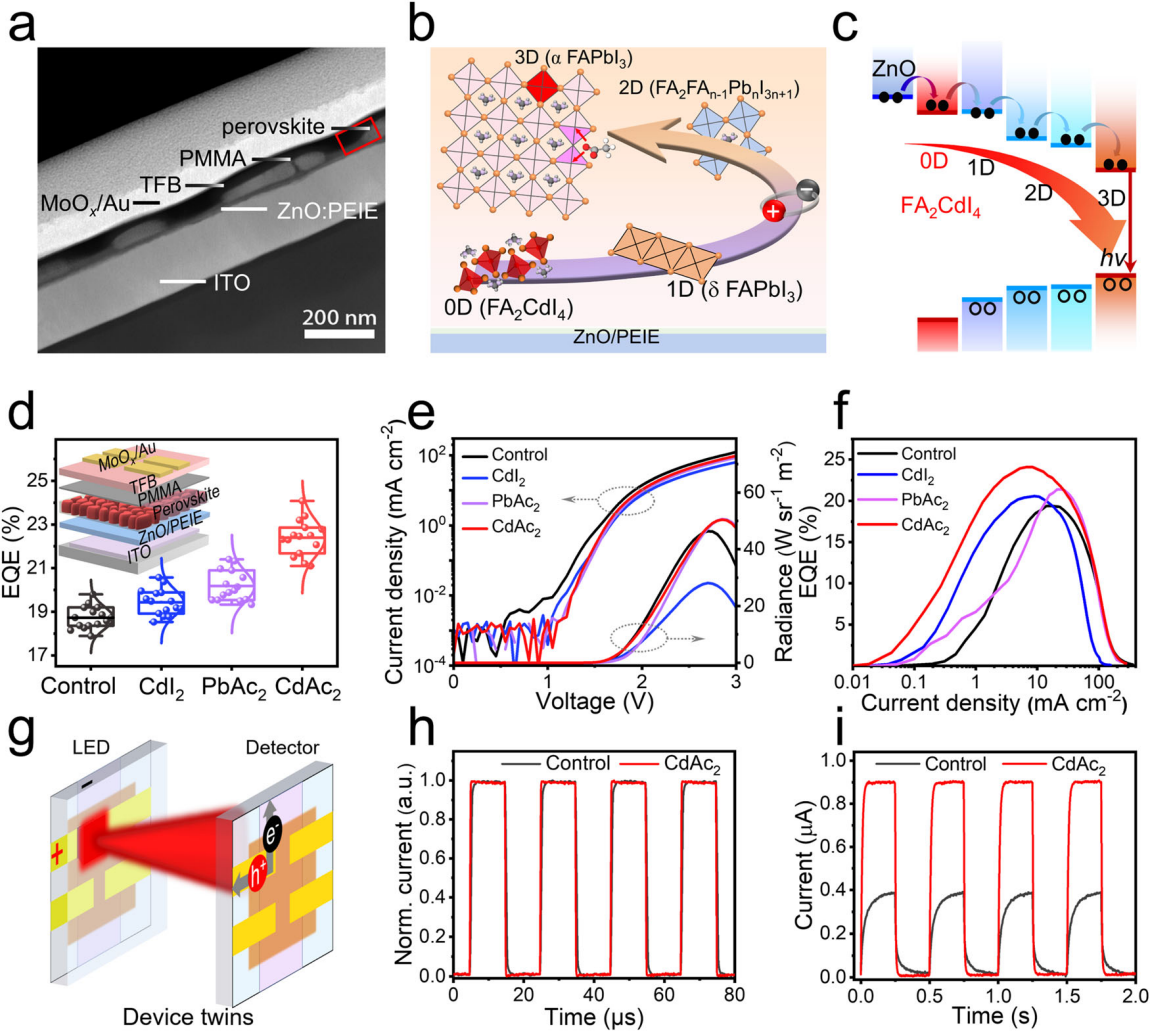
Schematic diagram of energy transfer mechanism and device performance. **a** HAADF-STEM image of a representative device. **b** Schematic diagram of the energy transfer process in perovskite light-emitting layers. **c** Schematic diagram of the excited carrier transfer in an enhanced energy funnel structure. **d** Statistical distribution of the peak EQE for control and PMs-added devices. Insert: device architecture schematic of as-fabricated FAPbI_3_ PeLEDs. **e** *J*–*V*–*R* and **f** EQE-*J* plots of devices with different PMs. **g** Schematic diagram of a self-driven detection system composed of device twins. Device characteristics of **h** electrical response and **i** self-driven detection of the optical response.

Encouraged by the significantly improved properties of the perovskite films, we then fabricated and examined the PeLEDs employing a classic structure of ITO/PEIE modified ZnO (ZnO: PEIE, ∼30 nm)/perovskite (∼50 nm)/polymethyl methacrylate (PMMA, ∼10 nm)/poly-(9,9-dioctyl-fluorene-co-N-(4-butylphenyl) diphenylamine) (TFB, 40 nm)/molybdenum oxide (MoO_*x*_, 6.5 nm)/gold (Au, 60 nm) in the LQ-100 system (Fig. 4a, Supplementary Fig. 21). And the PMMA was employed to clad the grains with no changes in the structure of the light out-coupling (Supplementary Fig. 22), thus artificially suppressing the ion migration as the literature suggested^8,50^. This discontinuous structure would not lead to leakage current because of the poorly conductive 5AVA and the insulating PMMA (Supplementary Fig. 23). The selected-area element mapping of the CdAc_2_-added device demonstrated the presence of another layer of Cd-I materials in the perovskite layer, which could be only related to 0D FA_2_CdI_4_ perovskitoid (Supplementary Fig. 24). Fig. 4b, c present an enhanced energy transfer mechanism in our CdAc_2_-added and FAI-rich FAPbI_3_ perovskite devices, starting from 0D FA_2_CdI_4_ to 1D δ-phase FAPbI_3_, 2D FA_2_FA_n-1_Pb_n_I_3n+1_ (*n* = 1, 2), and 3D FAPbI_3_ (Supplementary Fig. 25).

CdI_2_ (with Cd^2+^ cation alone) or PbAc_2_ (with Ac^-^ anion alone)-added perovskite devices were compared with CdAc_2_-added devices to verify the dual role of defects passivation and the 0D material. The electroluminescence (EL) spectra of all the devices displayed in Supplementary Fig. 26 demonstrated the same emission peak at 804 nm with FWHM of around 46 nm. While the CdI_2_ or PbAc_2_-based devices exhibited improved EQE values compared with the control ones, they were significantly behind the devices with the addition of multifunctional CdAc_2_ (Fig. 4d). And Fig. 4e gives current density-voltage (*J-V*) curves and their corresponding radiance plots of the best-performing devices with and without PMs. We can see that although the trend of *J* looks almost the same for all the devices, the radiances of PMs-passivated devices showed a little difference. This can be attributed to the presence of excess non-perovskite organics in the films after introducing the PMs. The best-performing device with CdAc_2_ passivation showed a peak radiance of 51.8 W sr^−1^ m^−2^. Figure 4f offers the corresponding EQE spectra of the devices. The CdAc_2_-passivated device reached a record efficiency of 24.1% at a relatively low current density of 7.2 mA cm^−2^, while the control one demonstrated a peak EQE of 19.6% at a current density of 22 mA cm^−2^. Such encouraging improvement is attributed to the defect passivation and effective carrier injection induced by the stepped-dimensional materials. Supplementary Fig. 26 provides more plentiful details about the light-emission performance of the devices with different concentrations of PMs, and the corresponding parameters are summarized in Supplementary Table 4.

Since temperature and FAI content are important factors for the formation of 1D and 2D phases, we changed the preparation process to fabricate PeLEDs with different phase combinations (Supplementary Fig. 27). The best EQE and operational stability in the case of a multidimensional combination of 0D, 1D, 2D, and 3D showed the the validity of CdAc_2_ additive and the superiority of the energy transfer mode from 0D to 1D, 2D, and 3D (Supplementary Table 5). To determine the veracity of the effect of 0D FA_2_CdI_4_ perovskitoid on the performance of devices, we also coated a FA_2_CdI_4_ insertion layer before deposition of the PbAc_2_-added FAPbI_3_ perovskite layer. The results presented in Supplementary Fig. 28 illustrate that the FA_2_CdI_4_ insertion layer combined with the passivation of Ac^-^ ions, is also able to achieve an enhanced EQE of 23.3%. Although slightly lower than 24.1%, it still shows the improvement of device performance by the 0D FA_2_CdI_4_ perovskitoid, as compared with the initial devices without any additive. The devices’ performance with different additives is counted in Supplementary Figure 29, and under the multifunctional CdAc_2_ additive, the efficiency is finally pushed to a record value of 24.1%. And with the aid of a refractive index matching the PDMS hemisphere for enhanced out-coupling efficiency, the best-performing PeLEDs successfully extracted the light trapped in the waveguide mode and further pushed the EQE to 29% (Supplementary Fig. 30).

For comparison, the performance of the devices reported in this work and others is listed in Supplementary Table 6. Notably, our devices exhibited high efficiencies at low currents, resulting from effective passivation and efficient carrier injection with minimized energy losses. Such properties should be more energy-efficient compared with organic light-emitting diodes (OLEDs) and quantum light-emitting diodes (QLEDs)^5^. In addition, a constant current density of 10 mA cm^−2^ was applied to three devices to test their operational stabilities, one without any PMs, one with a CdAc_2_ additive, and one with PbAc_2_ and a FA_2_CdI_4_ inserting layer (IL). The operation lifetime (*T*_50_, time to half of the initial radiance) of the encapsulated devices increased from 54.2 to 110.7, and 174.3 h for the control one, the CdAc_2_-added device, and the device with PbAc_2_ and a FA_2_CdI_4_ inserting layer, respectively (Supplementary Fig. 31). The improved device operational stability can be attributed to the suppression of ion migration, which is reflected in the increased ion-migration activation energy (*E*_a_) and the formation energy of mobile ions (*E*_d_) of perovskite films with defects anchoring by CdAc_2_ additive (Supplementary Fig. 32). Besides, the inhibition of the sustained deprotonation reaction of ZnO and FAI by inserting a FA_2_CdI_4_ layer is also one of the key factors to improve the stability^35,42,51,52^.

Beyond LEDs, our high-performance devices could also be assembled into a NIR-communication system with self-driving detection functions. The NIR- detection system was composed of device twins, in which one device served as a light source to provide a light signal at a square-wave voltage with a fixed frequency, and the other one acted as a detector to respond to the light signal emitted by the former device (Fig. 4g). Supplementary Figs. 33 and 34 gives a digital photograph of the device twins and a schematic diagram about how to measure the response of such a spectral response system. The device twins were tested after encapsulation in ambient air to avoid water and oxygen corrosion. We tested the electrical response of the as-fabricated devices with a digital oscilloscope and found that the devices could take about a few hundred nanoseconds to generate a balanced electrical signal against bias voltages (Fig. 4h, Supplementary Fig. 35). Thanks to the driving voltage, the electrical response in the order of hundreds of nanoseconds indicated that the proper transport layers and small charge barrier, as well as small parasitic capacitances, are what really counts^53^. Fewer electrons captured in the CdAc_2_-added device resulted in a faster electrical response than that of pristine. In comparison with previous reports^53^, our devices exhibited almost negligible overshoot currents. We speculated that it may be due to the introduction of insulative PMMA to block ion movement channels. On this basis, PeLEDs were extended to construct a spectral response system. Figure 4i illustrates the temporal light-response curves of the devices measured at a zero voltage. The light-response speed of the devices could be assessed by analyzing the rising and falling edges in a single response cycle (Supplementary Fig. 36). By contrast, the rise time reduced from 99.5 ms for the control one to 22.1 ms for the CdAc_2_-added device, and the fall time reduced from 77.9 to 8.3 ms, revealing an important role of CdAc_2_ in FAPbI_3_-based perovskites. The NIR detection performance parameters of the devices reported in this work and others is listed in Supplementary Table 7. The far-ahead electrical response time and comparable light-response time to conventional detection devices demonstrated the superior role of CdAc_2_ played on NIR PeLED performance.

To sum up, we have achieved high-efficiency near-infrared FAPbI_3_-based PeLEDs with minimized energy losses by introducing multifunctional additives of cadmium salts, i.e. CdI_2_, CdBr_2_, CdCl_2_, and CdAc_2_. On one hand, the representative CdAc_2_ devoted strong synergistic passivation effects through both anions and cations, thus reducing the nonradiative recombination induced by the defects on the grain surface. On the other hand, the addition of Cd^2+^ ions resulted in a 0D FA_2_CdI_4_ phase with a well-aligned conduction band, serving as an energy transfer ladder between ZnO electron injection layer and perovskite emitter and significantly reducing the energy losses during carrier injection. Moreover, at the annealing temperature of 105 °C, we have found that the precursors containing excess FAI can be inevitably partially converted into 1D non-perovskite δ-FAPbI_3_ and 2D FA_2_FA_n-1_PbI_3n+1_ (*n* = 1, 2) impurity phases, which are not harmful but beneficial for the energy transfer and carrier injection by combining the 0D FA_2_CdI_4_. Therefore, the resultant near-infrared PeLEDs loading the optimal and multifunctional CdAc_2_ additive and stepped-dimensional materials realized a record EQE of 24.1% along with enhanced operational stability. In addition to emitting light, our optimized devices could also enable a promising NIR-communication system composed of device twins. In such a system, one is for light emitting and the other is for self-driven detection because of their good light-response and electrical response properties, which greatly extends the application of PeLEDs to the field of infrared light communication. These results elucidate a fascinating mechanism of carrier transport and energy transfer with formamidinium-based stepped-dimensional perovskite analogs and suggest an effective strategy using multifunctional cadmium salt additives for improving defect passivation, carrier transport, and energy transfer. This work provides a feasible route to improve the near-infrared PeLEDs’ performance as well as expand their applications.

## Methods

### Precursor preparation

The formamidinium lead iodide (FAPbI_3_) perovskite precursors consisted of formamidinium hydroiodide (FAI, Advanced Election Technology), lead iodide (PbI_2_, Alfa Aesar), 5-aminovaleric acid (5AVA, Aladdin), and passivation molecules [PMs: cadmium iodide (CdI_2_), or lead acetate (PbAc_2_), or cadmium acetate (CdAc_2_), Sigma-Aldrich] with a molar ratio of FAI: PbI_2_: 5AVA: PMs = 2:1:0.85:*x* (*x* = 0~0.2), which were dissolved in N, N-dimethylformamide (DMF). The concentration of Pb^2+^ should be kept at 0.075 M. The precursors were stirred for 12 h at 55 °C in a nitrogen-filled glovebox before use. The zinc oxide (ZnO) nanocrystals were synthesized according to the literature^1,31^.

### Single-crystal growth details

FA_2_CdI_4_ crystals were grown through slow evaporation of their stoichiometric (2:1 molar ratio of FAI to CdI_2_) solutions. The initial precursors were kept at 1 M with FA_2_CdI_4_ methanol solutions. Single crystals of FA_2_CdCl_4_ with about 5 mm in size were grown at room temperature in a quiet environment.

### Device fabrication

Indium tin oxide (ITO) substrates were sonicated in acetone, isopropanol, and ethanol for 10 min in turn and then treated by UV-OZONE to facilitate the spin coating of ZnO electron injection layers. The clean substrates were quickly covered with ZnO at 4000 rpm for 45 s and then an ultra-thin layer of poly-ethylenimine ethoxylated (PEIE) was spin-coated on top of ZnO at 5000 rpm for 45 s. The ZnO-PEIE substrates were then transferred into a nitrogen-filled glovebox and annealed at 90 °C for 10 min. For the additional ultra-thin FA_2_CdI_4_ layers, the FA_2_CdI_4_ precursors with a stoichiometric molar ratio (0.12 mg/ml) were spin-coated on the ZnO/PEIE substrates and annealed at 100 °C for 5 min (about 5 nm). After that, the perovskite layers were spin-coated at 4000 rpm for 45 s and annealed at 100 °C for 30 min on a pre-heated hotplate. In the following, polymethyl methacrylate (PMMA) (10 mg ml^−1^ in chlorobenzene) layers were spin-coated onto the perovskites at 6000 rpm for 60 s. Whereafter, Poly-[(9,9-dioctylfluorenyl-2,7-diyl)-co-(4,4′-(N-(pbutylphenyl)) diphenylamine)] (TFB, 13 mg ml^-1^ in chlorobenzene) layers were spin-coated at 3000 rpm for 35 s. Finally, 6.5 nm of molybdenum oxide (MoO_*x*_) and 60 nm of Au were deposited as the electrode by thermal evaporation at a pressure of 2.4 × 10^−4^ Pa. The active area of the devices was 4 mm^2^.

### Device performance measurements

The device performance of perovskites light-emitting diodes (PeLEDs) was recorded on an external quantum efficiency (EQE) system (Enli Technology Co. Ltd) in a nitrogen-filled glove box at room temperature. This system includes an integrating sphere, a source meter (Keithley 2901), and a spectrometer. For the EQE test of the devices, the basis voltages started from 0 V and increased with a step of 0.05 V, and at each voltage step, it lasted 500 ms for stabilization of measurements. For the operational stability test of the devices, a steady and constant current is fed to the device through the probe at the tip of the integrating sphere. Here we just collect the absolute spectral integrating value of the electroluminescence at different times through the integrating sphere, e.g. collected every 10 min, thus directly calculating the EQE of the device. Stability data can be obtained by comparing the EQE results at different times with the original EQE.

### Space-charge-limited current (SCLC) measurements

SCLC measurements were conducted to estimate the trap density of perovskite films.

### Photoluminescence (PL) and photoluminescence quantum efficiency (PLQE) measurements

PL measurements were characterized by a system with an integrating sphere and an excitation wavelength of 365 nm. The fixed light intensity of 100 mW cm^−2^ and a LED source with variable intensity from 0.01 to 108 mW cm^−2^ were used for the PL and PLQE measurements, respectively. PL measurements at a Kelvin temperature of 80 K were also carried out by a laser with a 260 nm wavelength and the results are shown in Fig. 2g.

### Time-resolved photoluminescence (TRPL) measurements

TRPL measurements were obtained with a Delta Flex fluorescence spectrum spectroscopy (HORIBA). Excitation intensity for the TRPL measurements was 0.03 μJ cm^-2^, and the excitation wavelength was 481 nm. The TRPL spectra were fitted by a biexponential function of y = A_1_*exp(−x/*τ*_1_) + A_2_*(−x/*τ*_2_), where the A_1_ and A_2_ are the normalized pre-exponential factors, and the *τ*_1_ and *τ*_2_ are the lifetimes of the fast and slow decay component, respectively. The average carrier lifetime (*τ*_av_) can be calculated by the following equation:

1 $${{{{{{\boldsymbol{\tau }}}}}}}_{{av}}=\frac{{A}_{1}{\tau }_{1}^{2}+{A}_{2}{\tau }_{2}^{2}}{{{A}_{1}\tau }_{1}+{{A}_{2}\tau }_{2}}$$

### Ultraviolet-visible (UV–Vis) absorption measurements

Absorbance spectra of perovskite films were measured by a UV-vis spectrophotometer (SHIMADZU mini 1280). Thin film samples without special instructions were coated on quartz substrates. The Urbach energy (*E*_u_) of the samples was fitted by the equation of ${{{{{\rm{\alpha }}}}}}={{{{{{{\rm{\alpha }}}}}}}_{0}}^{hv/{E}_{u}}$, where the α is the wavelength-dependent absorption coefficient, α_0_ is a constant related to the absorption, and *hv* is the photon energy. The *E*_u_ can be calculated by the following transformed equation:

2 $${{{{{\rm{Ln}}}}}}\left(\alpha \right)=\frac{1}{{E}_{u}}{{\cdot }}\left({hv}\right)-{{{{{\rm{Ln}}}}}}{{{{{{\rm{\alpha }}}}}}}_{0}$$

### Scanning electron microscopy (SEM) measurements

Low-magnified film morphology was obtained by a JSM-IT300 scanning electron microscope (SEM). And cross-sectional structures of devices were collected with a Zeiss SIGMA field-emission scanning electron microscope (FE-SEM).

### Film X-ray diffraction (XRD) measurements

Crystal structures of perovskites were examined by a Rigaku smartlab X-ray diffractometer (XRD) with Cu Kα radiation under operating conditions of 40 kV and 44 mA. All samples for XRD testing were prepared on extremely thin ZnO substrates.

### High-angle annular dark-field scanning transmission electron microscopy (HAADF-STEM) measurements

The distribution of elements was measured by a high-angle annular dark-field scanning transmission electron microscope (HAADF-STEM) (FEI Themis Z).

### X-ray photoelectron spectroscopy (XPS) and ultraviolet photoelectron spectroscopy (UPS) measurements

XPS and UPS spectra were performed using an XPS/UPS system (ESCLAB 250Xi, Thermo Scientific). The XPS used a monochromatic Mg Kα radiation as the excitation source, and UPS used a He Iα radiation. The work function (*W*_f_) of samples can be calculated by the equation of *E*_f_ = 21.22 eV– *E*_cutoff_, where the *E*_f_ is the Fermi level and the *E*_cutoff_ is the cutoff of the UPS spectrum in the high binding energy range. The difference between the valence band maximum (*E*_VBM_) and *E*_f_ was extracted from the cutoff of UPS spectra in the low binding energy range, and the conduction band minimum (*E*_CBM_) can be calculated via the equation of *E*_CBM_ = *E*_VBM_ + *E*_g_, where the *E*_g_ is the optical bandgap.

### Atomic force microscopy (AFM) and Kelvin probe force microscopy (KPFM) measurements

AFM height images and KPFM potential distribution images were attained in the ambient atmosphere by a Bruker Dimension Icon XR AFM. In the KPFM measurements, the voltages were applied to the samples and the contact electrical potential difference (CPD) was consistent with the work function of the samples. Thus, a higher CPD value represented a higher work function, which means a deeper Fermi level.

### Transient absorption (TA) measurements

TA spectra were measured by an ultrafast transient absorption spectrometer (HARPIA, Light Conversion). In this system, the output of a femtosecond laser (repetition rate of 40 kHz, 1030 nm central wavelength, and pulse duration of ~ 120 fs, PHAROS, Light Conversion) was split into two parts: One went through the optical parametric amplification (ORPHEUS twins, Light Conversion) and then became a pump pulse to excite perovskite thin films; the other went through the delay stage and produced white light via sapphire and then acted as the probe beam. The pump and probe light focused on the samples with a spot size of about 64 µm. Owing to the small angle between these two beams, the pump pulse was blocked by a pinhole, and the probe was therefore collected with a spectrometer.

### Ion-migration measurements

The conductivity (${{{{{\rm{\sigma }}}}}}$) of ion migration depends on the energy barrier at a low-temperature range (i.e. activation energy, *E*_a_) and the formation energy of movable ions (*E*_d_) at a high-temperature range. There is the following relationship according to the Arrhenius formula^54^:

3 $$\,{{{{{\rm{\sigma }}}}}}{{{{{\rm{\propto }}}}}}{{{{{\rm{exp }}}}}}\left(-\frac{{E}_{{{{{\rm{a}}}}}}}{{k}_{{{{{\rm{B}}}}}}T}\right){{{{{\rm{exp }}}}}}(-\frac{{E}_{{{{{\rm{d}}}}}}}{{2k}_{{{{{\rm{B}}}}}}T})$$

where *k*_B_*T* is the thermal activation energy, *k*_B_ and *T* are the Boltzmann constant and temperature, respectively. Therefore, the activation energy *E*_a_ (the low-temperature range of neglected thermal excitation: intrinsically movable ions) and the sum of the activation energy and the half-value of the formation energy (*E*_a_′ = *E*_a_ + *E*_d_/2 at the high-temperature range: thermal-excited region) can be expressed by the slope in the Arrhenius plot of Ln*σ*-1/*k*_*B*_*T*.

### Photoelectric detection measurements

The measurements of electrical response were carried out using an arbitrary waveform generator (KEYSIGHT, 33600 A). The currents that varied with the waveform output were detected by a digital storage oscilloscope (KEYSIGHT DXOX6002A). For the light-emitting and self-driven detection system, a Keysight B2912A was used to supply constant currents to the light-emitting devices and the same digital storage oscilloscope was used to detect the photocurrents of devices on the opposite side.

### DFT calculations

All first-principle calculations were performed with the Vienna Ab-initio Simulation Package (VASP). Electronuclei interactions were described by the projector-augmented wave Pseudopotentials. The Perdew–Burke–Ernzerhof exchange-correlation functional was investigated in all calculations. The Kohn–Sham wave functions were expanded in plane waves up to 400 eV. A K-point mesh of 3 × 3 × 1 for 2 × 2 × 1 slab supercell of (001) surface was used. The slab models were built with a vacuum region of more than 15 Å in the Z-direction in conjunction with the dipole correction to avoid the fictitious interaction with its periodic images. The convergence threshold of energy in the self-consistent step was set as 10^−5^ eV with a Gaussian smearing of 0.01 eV Å^−1^. Moreover, the force threshold of geometry optimation was 0.02 eV Å^−1^ for each dimension of all atoms in a supercell. We did not involve the SOC approximation in the simulation process because of the computational configuration limitations. And the numerical calculations in this paper were completed in the supercomputing system in the Supercomputing Center of Wuhan University.

### Accession codes

CCDC (2219290) contain the supplementary crystallographic data of FA_2_CdI_4_ perovskitoid for this paper. These data can be obtained free of charge via www.ccdc.cam.ac.uk/data_request/cif.

## Supplementary information

Supplementary Information

Description of Additional Supplementary Files

Supplementary Data 1

## Data Availability

The authors declare that the main data supporting the results in this study are available within the article and supplementary files, which is also available from the corresponding authors upon request. Source data are provided with this article. Source data are provided with this paper.

## Source data

Source Data

## References

[1] Cao Y (2018). Perovskite light-emitting diodes based on spontaneously formed submicrometre-scale structures. Nature.

[2] Stranks SD, Snaith HJ (2015). Metal-halide perovskites for photovoltaic and light-emitting devices. Nat. Nanotechnol..

[3] Fakharuddin A (2022). Perovskite light-emitting diodes. Nat. Electron..

[4] Liu XK (2021). Metal halide perovskites for light-emitting diodes. Nat. Mater..

[5] Vasilopoulou M (2021). Advances in solution-processed near-infrared light-emitting diodes. Nat. Photonics.

[6] Chu Z (2021). Perovskite light-emitting diodes with external quantum efficiency exceeding 22% via small-molecule passivation. Adv. Mater..

[7] Fang, Z. et al. Dual passivation of perovskite defects for light-emitting diodes with external quantum efficiency exceeding 20%. *Adv. Funct. Mater.* **30**, 1909754 (2020).

[8] Jia Y (2021). Excess ion-induced efficiency roll-off in high-efficiency perovskite light-emitting diodes. ACS Appl Mater. Interfaces.

[9] Lin K (2018). Perovskite light-emitting diodes with external quantum efficiency exceeding 20 per cent. Nature.

[10] Liu Z (2021). Perovskite light-emitting diodes with EQE exceeding 28% through a synergetic dual-additive strategy for defect passivation and nanostructure regulation. Adv. Mater..

[11] Ma D (2021). Distribution control enables efficient reduced-dimensional perovskite LEDs. Nature.

[12] Shen Y (2019). High-efficiency perovskite light-emitting diodes with synergetic outcoupling enhancement. Adv. Mater..

[13] Xu W (2019). Rational molecular passivation for high-performance perovskite light-emitting diodes. Nat. Photonics.

[14] Guo B (2022). Ultrastable near-infrared perovskite light-emitting diodes. Nat. Photonics.

[15] Jin H (2020). It’s a trap! On the nature of localised states and charge trapping in lead halide perovskites. Mater. Horiz..

[16] Lee J-W (2017). A bifunctional lewis base additive for microscopic homogeneity in perovskite solar cells. Chem.

[17] Wu Y (2019). Liquid metal acetate assisted preparation of high-efficiency and stable inverted perovskite solar cells. J. Mater. Chem. A.

[18] Wang S (2020). Lewis acid/base approach for efficacious defect passivation in perovskite solar cells. J. Mater. Chem. A.

[19] Kong L (2021). Smoothing the energy transfer pathway in quasi-2D perovskite films using methanesulfonate leads to highly efficient light-emitting devices. Nat. Commun..

[20] Kim D, Ahmadi M (2021). Elucidating the spatial dynamics of charge carriers in quasi-two-dimensional perovskites. ACS Appl Mater. Interfaces.

[21] Sun C (2021). High-performance large-area quasi-2D perovskite light-emitting diodes. Nat. Commun..

[22] Zhao B (2020). Efficient light-emitting diodes from mixed-dimensional perovskites on a fluoride interface. Nat. Electron..

[23] Jia YH (2019). Role of Excess FAI in formation of high‐efficiency FAPbI_3_‐based light‐emitting diodes. Adv. Funct. Mater..

[24] Guo Y (2020). Degradation mechanism of perovskite light‐emitting diodes: an in situ investigation via electroabsorption spectroscopy and device modelling. Adv. Funct. Mater..

[25] Wang N (2016). Perovskite light-emitting diodes based on solution-processed self-organized multiple quantum wells. Nat. Photonics.

[26] Navas J (2015). New insights into organic-inorganic hybrid perovskite CH_3_NH_3_PbI_3_ nanoparticles. An experimental and theoretical study of doping in Pb^2+^ sites with Sn^2+^, Sr^2+^, Cd^2+^ and Ca^2+^. Nanoscale.

[27] Ilyas BM, Elias BH (2017). A theoretical study of perovskite CsXCl_3_ (X=Pb, Cd) within first principles calculations. Phys. B Condens. Matter.

[28] Roccanova R (2017). Synthesis, crystal and electronic structures, and optical properties of (CH_3_NH_3_)_2_CdX_4_ (X = Cl, Br, I). Inorg. Chem..

[29] Yuan S (2019). Simultaneous cesium and acetate coalloying improves efficiency and stability of FA_0.85_MA_0.15_PbI_3_ perovskite solar cell with an efficiency of 21.95%. Solar RRL..

[30] Kim BJ, Boschloo G (2021). Beneficial effects of cesium acetate in the sequential deposition method for perovskite solar cells. Nanoscale.

[31] Yuan Z (2019). Unveiling the synergistic effect of precursor stoichiometry and interfacial reactions for perovskite light-emitting diodes. Nat. Commun..

[32] Yuan M (2016). Perovskite energy funnels for efficient light-emitting diodes. Nat. Nanotechnol..

[33] Stoumpos CC (2016). Ruddlesden–popper hybrid lead iodide perovskite 2D homologous semiconductors. Chem. Mater..

[34] Mao L, Stoumpos CC, Kanatzidis MG (2019). Two-dimensional hybrid halide perovskites: principles and promises. J. Am. Chem. Soc..

[35] Teng P (2021). Degradation and self-repairing in perovskite light-emitting diodes. Matter.

[36] Liu N, Yam C (2018). First-principles study of intrinsic defects in formamidinium lead triiodide perovskite solar cell absorbers. Phys. Chem. Chem. Phys..

[37] He J, Long R (2018). Lead vacancy can explain the suppressed nonradiative electron-hole recombination in FAPbI_3_ perovskite under iodine-rich conditions: a time-domain ab initio study. J. Phys. Chem. Lett..

[38] Ma D (2021). An effective strategy of combining surface passivation and secondary grain growth for highly efficient and stable perovskite solar cells. Small.

[39] Watthage SC (2017). Enhanced grain size, photoluminescence, and photoconversion efficiency with cadmium addition during the two-step growth of CH_3_NH_3_PbI_3_. ACS Appl Mater. Interfaces.

[40] Tu B (2019). Novel molecular doping mechanism for n-doping of SnO_2_ via triphenylphosphine oxide and its effect on perovskite solar cells. Adv. Mater..

[41] Li F (2020). Regulating surface termination for efficient inverted perovskite solar cells with greater than 23% efficiency. J. Am. Chem. Soc..

[42] Miao Y (2019). Stable and bright formamidinium-based perovskite light-emitting diodes with high energy conversion efficiency. Nat. Commun..

[43] Keeble DJ (2021). Identification of lead vacancy defects in lead halide perovskites. Nat. Commun..

[44] Zhu L (2021). Unveiling the additive-assisted oriented growth of perovskite crystallite for high performance light-emitting diodes. Nat. Commun..

[45] Lu J (2022). Formamidinium-based Ruddlesden–Popper perovskite films fabricated via two-step sequential deposition: quantum well formation, physical properties and film-based solar cells. Energy Environ. Sci..

[46] Rosales BA (2020). Reversible multicolor chromism in layered formamidinium metal halide perovskites. Nat. Commun..

[47] Li Z (2015). Stabilizin perovskite structures by tuning tolerance factor: formation of formamidinium and cesium lead iodide solid-state alloys. Chem. Mater..

[48] Ma F (2017). Stable alpha/delta phase junction of formamidinium lead iodide perovskites for enhanced near-infrared emission. Chem. Sci..

[49] He T, Jiang Y, Xing X, Yuan M (2020). Structured perovskite light absorbers for efficient and stable photovoltaics. Adv. Mater..

[50] Ava T (2021). Role of PMMA to make MAPbI_3_ grain boundary heat-resistant. Appl. Surf. Sci..

[51] Ye F, Shan Q, Zeng H, Choy WCH (2021). Operational and spectral stability of perovskite light-emitting diodes. ACS Energy Lett..

[52] Kim H (2020). Proton-transfer-induced 3D/2D hybrid perovskites suppress ion migration and reduce luminance overshoot. Nat. Commun..

[53] Kumawat NK, Tress W, Gao F (2021). Mobile ions determine the luminescence yield of perovskite light-emitting diodes under pulsed operation. Nat. Commun..

[54] Yuan Y (2015). Photovoltaic Switching Mechanism in Lateral Structure Hybrid Perovskite Solar Cells. Adv. Energy Mater..

