# Supplementary material for: Synergistic passivation and stepped-dimensional perovskite analogs enable high-efficiency near-infrared light-emitting diodes
Source: Nat Commun. 2022 Dec 2;13:7425. doi: 10.1038/s41467-022-35218-0 (PMC9718757; doi:10.1038/s41467-022-35218-0)
Supplement: Supplementary file 1 — Supplementary Information [file 41467_2022_35218_MOESM1_ESM.pdf]

Supplementary Information

Synergistic passivation and stepped-dimensional perovskite analogs enable high-efficiency near-infrared light-emitting diodes

Yongjie Liu[1,#], Chen Tao[1,#], Yu Cao[2,3], Liangyan Chen[4], Shuxin Wang[1], Pei Li[1], Cheng Wang[1], Chenwei Liu[1], Feihong Ye[1], Shengyong Hu[5], Meng Xiao[1], Zheng Gao[1], Pengbing Gui[1], Fang Yao[1], Kailian Dong[1], Jiashuai Li[1], Xuzhi Hu[1], Hengjiang Cong[6], Shuangfeng Jia[1], Ti Wang[1], Jianbo Wang[1], Gang Li[7], Wei Huang[2,3], Weijun Ke[1,✉], Jianpu Wang[2,✉], Guojia Fang[1,✉]

[1]Key Lab of Artificial Micro- and Nano-Structures of Ministry of Education of China, School of Physics and Technology, Wuhan University, Wuhan, China

[2]Key Laboratory of Flexible Electronics (KLOFE) and Institute of Advanced Materials (IAM), Jiangsu National Synergetic Innovation Center for Advanced Materials (SICAM), Nanjing Tech University (Nanjing Tech), Nanjing, China

[3]Institute of Flexible Electronics, Northwestern Polytechnical University (NPU), Xi'an, China

[4]School of Electrical and Electronic Engineering, Wuhan Polytechnic University, Wuhan, China

[5]National Laboratory of Solid-State Microstructures, School of Physics, Nanjing University, Nanjing, China

[6]College of Chemistry & Molecular Sciences, Wuhan University, Wuhan, China

[7]Department of Electronic and Information Engineering, The Hong Kong Polytechnic University, Hong Kong, China

Email: weijun.ke@whu.edu.cn; iamjpwang@njtech.edu.cn; gjfang@whu.edu.cn

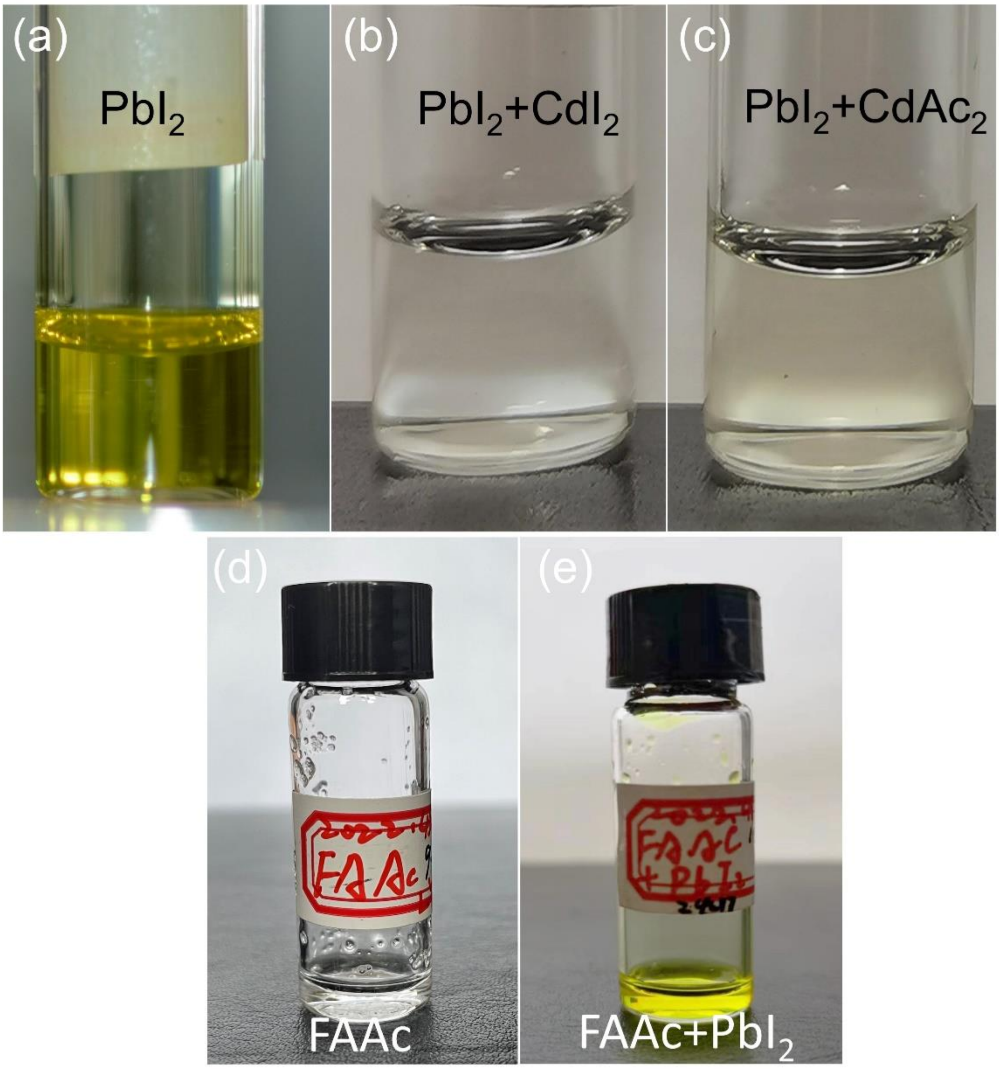

Supplementary Figure 1. The evidence for the interaction between Cd^{2+} and PbI_2, Pb^{2+} and Ac^-. The digital photos show the solutions of **(a)** PbI_2 dissolved in DMF, and the PbI_2 solutions added with the same molar amount of **(b)** CdI_2 and **(c)** $CdAc_2$. **(d)** FAAc dissolved in DMF, and **(e)** the solution with the addition of the same molar amount of PbI_2.

The changes in PbI_2 solution color indicated the interaction between cadmium and iodine was stronger than that between lead and iodine. The addition of PbI_2 to FAAc also promoted the dissolution of FAAc. The corresponding dried powders were used for XPS and FTIR measurements to demonstrate the coordination bonding between Pb, I, Cd, and Ac. The corresponding results are given in **Supplementary Figure 4-5**.

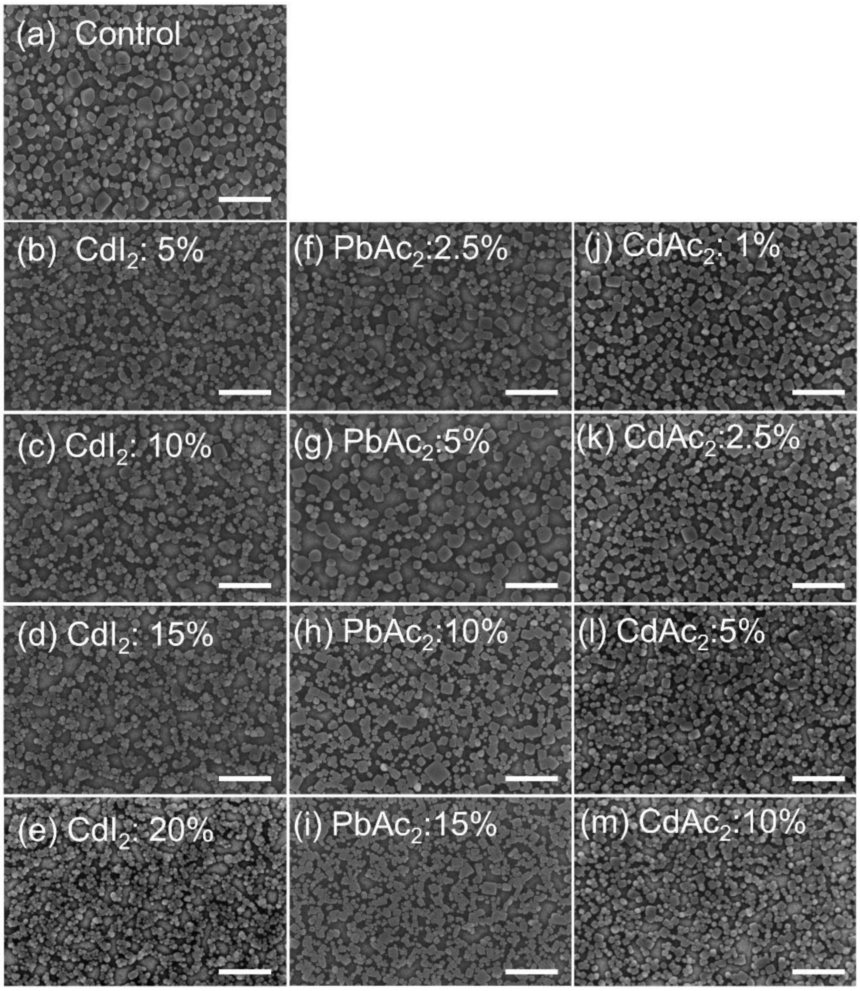

Supplementary Figure 2. SEM images of the $FAPbI_3$ perovskite films with different amounts of PMs. **(a)** A perovskite film without any PMs. The perovskite films with **(b)** 5%, **(c)** 10%, **(d)** 15%, and **(e)** 20% molar ratio of CdI_2. Perovskite films with **(f)** 2.5%, **(g)** 5%, **(h)** 10%, and **(i)** 15% molar ratio of $PbAc_2$. Perovskite films with **(f)** 1%, **(g)** 2.5%, **(h)** 5%, and **(i)** 10% molar ratio of $CdAc_2$. The scale bars for all images are 1 μm.

All the films were self-assembled and formed on ITO/ZnO: PEIE substrates. The amount of the additives was relative to the fixed 0.075 M Pb^{2+} concentration. The perovskite films with CdI_2 additive (**Supplementary Figure 2a-d**) showed increasingly smaller and more and more uniform crystallization as the content of CdI_2 increased, which would improve the repeatability of resultant devices. We speculate that the Cd^{2+} could provide more nucleation sites in the initial growth stage of perovskites and promote the formation of more crystal grains, thus leading to the smaller grains and uniform distribution. The addition of $PbAc_2$ was then used to investigate the effects of Ac^{2-} on the perovskite films alone (**Supplementary Figure 2e-h**). Compared with Cd^{2+}, the effects of Ac^- on perovskite grains were slightly weak but relatively distinct. The addition of Ac^- would result in the grains more cubic in characteristic. $CdAc_2$ was then introduced in the precursors to see the possible synergetic effects of Cd^{2+} and Ac^{2-}. As shown in **Supplementary Figure** 2i-l, the morphology changes of perovskite films with $CdAc_2$ were not as great as that with CdI_2 and $PbAc_2$, while they still showed more square-shaped crystals and displayed a dense tendency as the content of $CdAc_2$ increased.

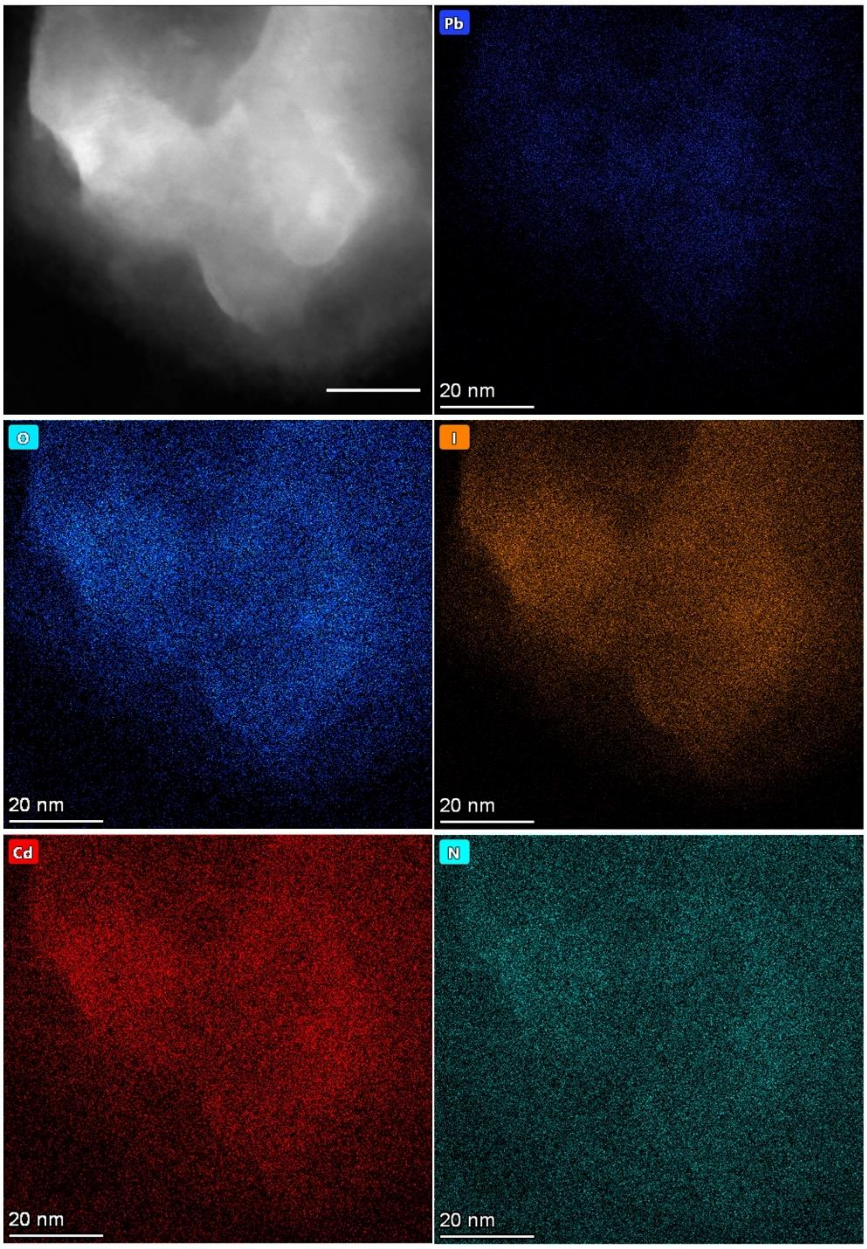

Supplementary Figure 3. HAADF-STEM and EDS mapping images of individual $FAPbI_3$ grains. The scale bars for all images are 20 nm.

Note that this sample was the $CdAc_2$-added $FAPbI_3$ perovskite grain without the addition of 5AVA. It can be seen that the oxygen (which could reflect the distribution of Ac^-) and cadmium was uniformly distributed on the surface of the grains.

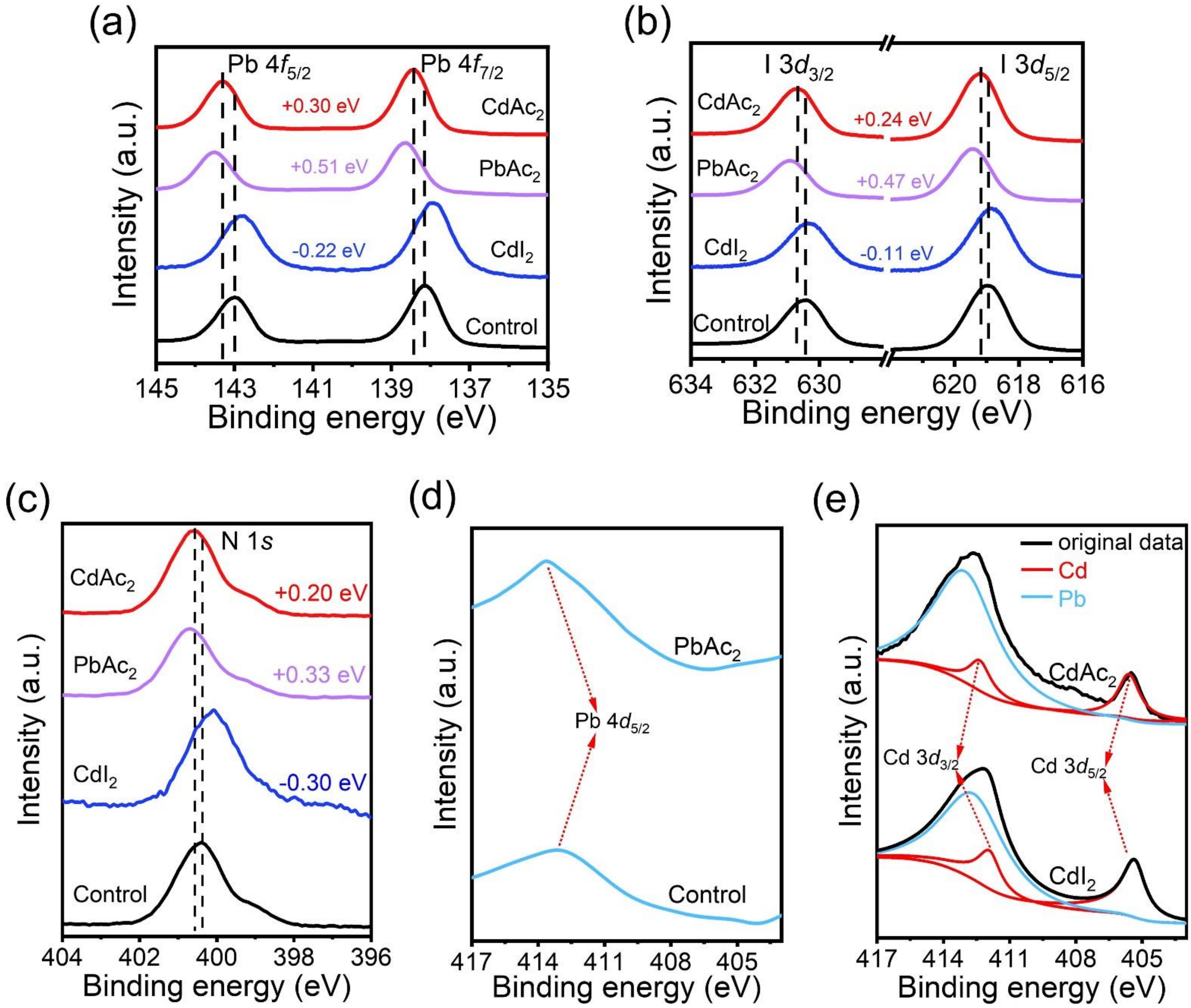

Supplementary Figure 4. XPS core level spectra of the films with different PMs. The **(a)** Pb 4*f*, **(b)** I 3*d*, **(c)** N 1*s*, **(d)** Pb $4d_{5/2}$, and **(e)** Cd 3*d* core levels' XPS spectra of the $FAPbI_3$ films with different PMs.

All the samples for XPS measurements were deposited on ZnO: PEIE/glass substrates and all XPS data were corrected with the binding energy of absorbed carbon at 284.8 eV. The change in binding energy implies a change in the density of the electron cloud outside the nucleus[1], which is related to the interaction among the elements. The opposite change direction of binding energy mainly stemmed from the different electron attraction ability of oxygen in Ac^- and Cd^{2+}, when $PbAc_2$ and CdI_2 were added separately.

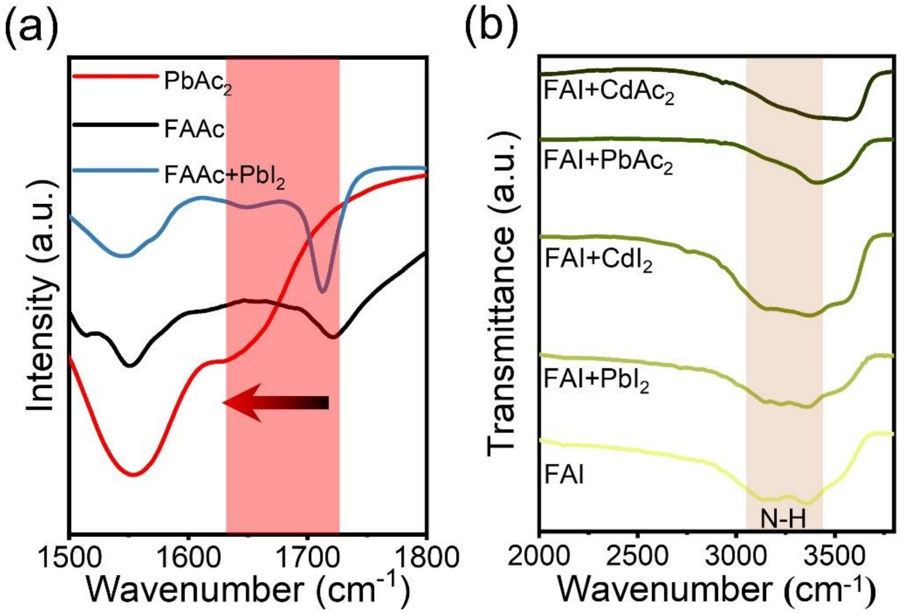

Supplementary Figure 5. FTIR spectroscopy results. (a) FTIR spectra of $PbAc_2$, FAAc powders, and the mixtures of FAAc with PbI_2. **(e)** FTIR spectra of $PbAc_2$, $CdAc_2$, CdI_2, and PbI_2 powders and their respective mixtures with FAI. Note that the powders for FTIR were obtained by drying the precursor solutions in DMF.

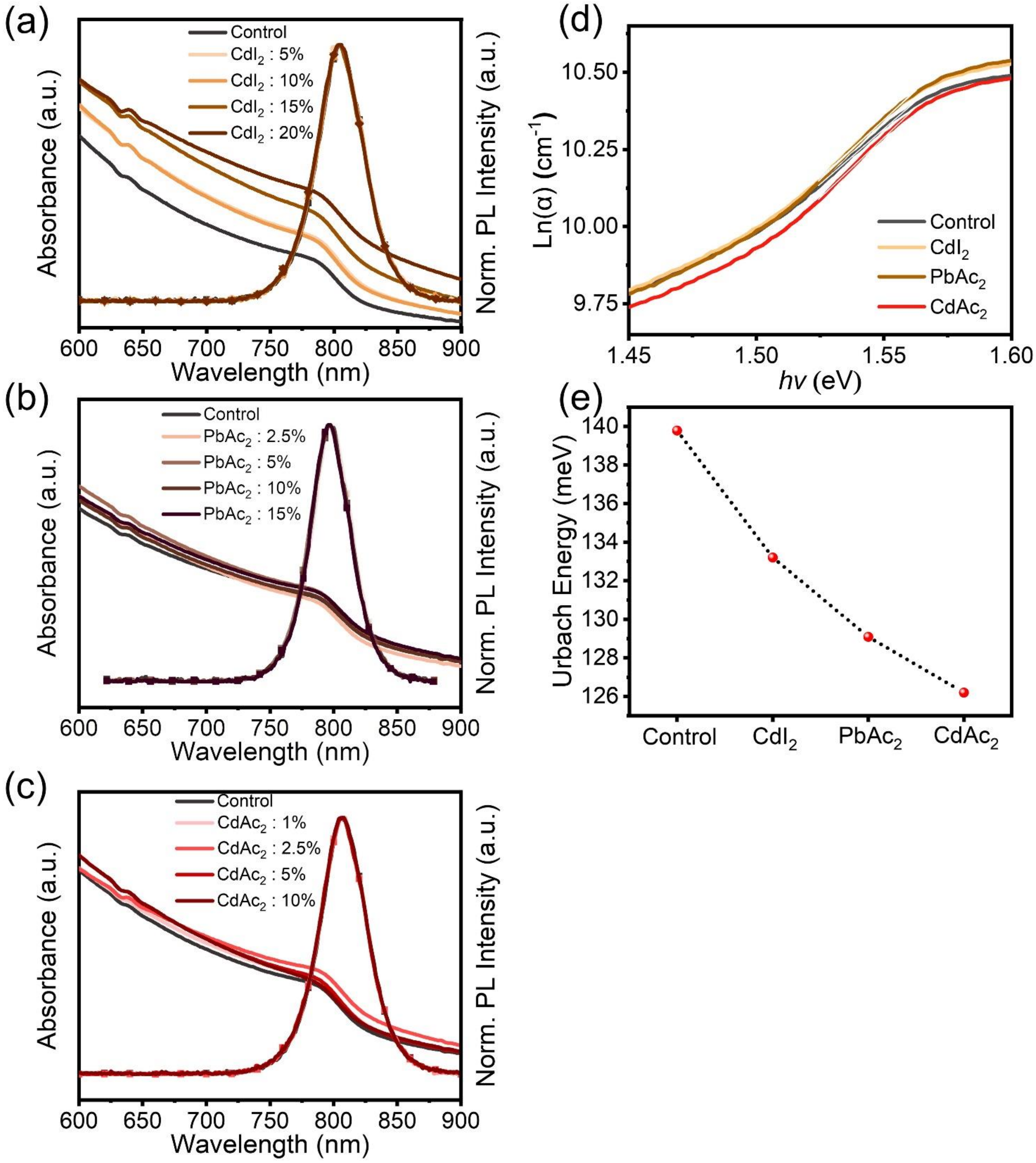

Supplementary Figure 6. Spectral characterization of absorption and normalized PL of different perovskite films. Absorption and PL spectra of $FAPbI_3$ perovskite films with different amounts of **(a)** CdI_2, **(b)** $PbAc_2$, and **(c)** $CdAc_2$; **(d)** Ln(α)-$h\nu$ plots and **(e)** the corresponding Urbach energies of perovskite films with different additives (with their optimal concentrations, i.e. CdI_2: 10%, $PbAc_2$: 5%, and $CdAc_2$: 2.5%).

These results demonstrate that the optical energy-bandgap and PL peak position of perovskite films did not change with the additives, while the Urbach energy of the films decreased with the addition of PMs, indicating reduced band-edge defects and increased crystal quality.

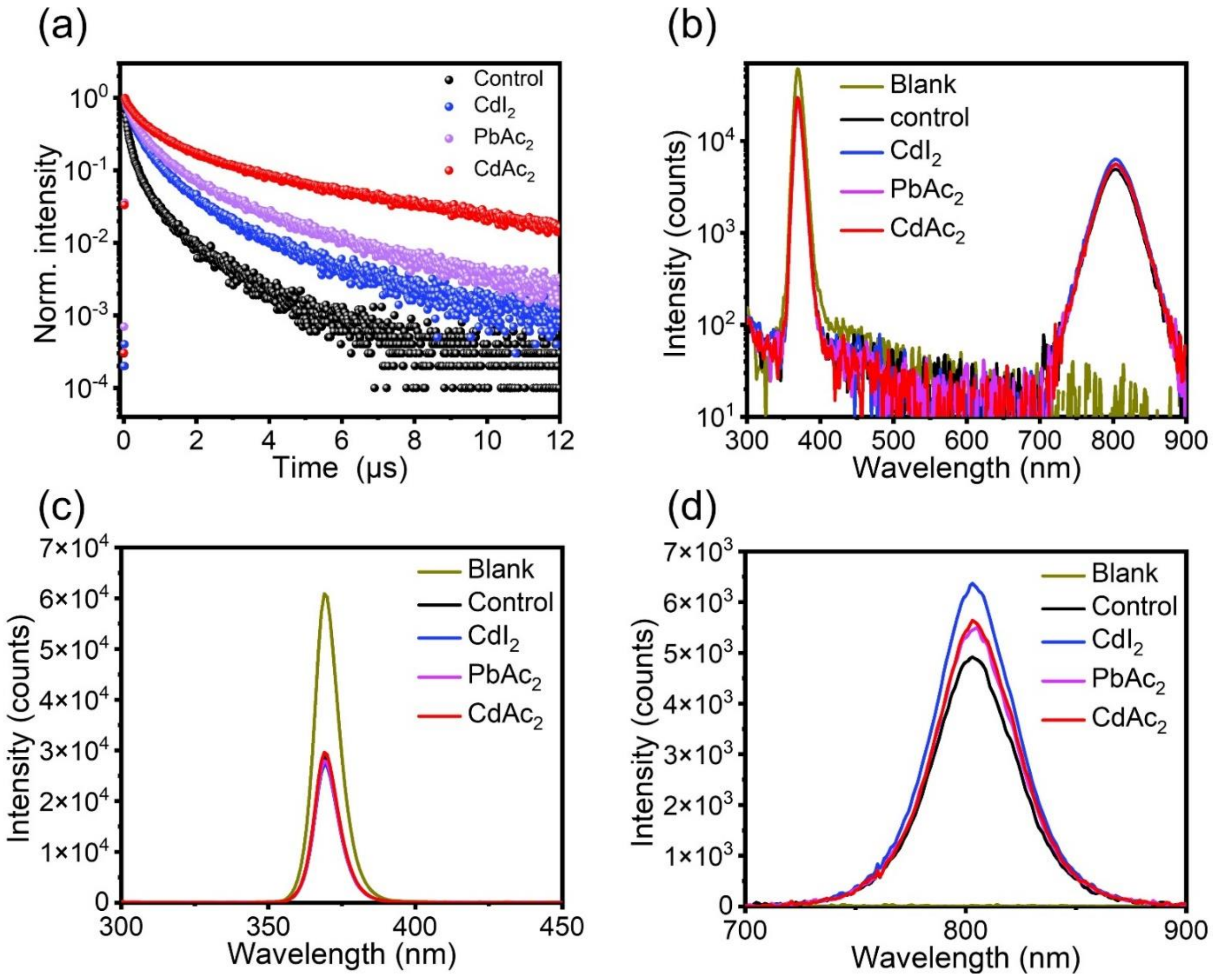

Supplementary Figure 7. Optical characterization of perovskite films. (a) TRPL spectra of perovskite films with different PMs. **(b)** PLQE spectra of perovskite films with different PMs. **(c)** Reflected and blank, and **(d)** emission spectra of perovskite films with different PMs.

After fitting the TRPL spectra with a biexponential function, we can see that the average carrier lifetime (τ_{av}) in perovskite films increased from 0.5 μs for the control film to 2.1 μs for the one with $CdAc_2$ passivation. Moreover, the PLQE of the films with different PMs was improved by about 10% compared to the pristine film at 20 $mW \cdot cm^{-2}$. **Supplementary Table 1** gives more detailed integration results. Radiative and nonradiative recombination rates (k_{rad} and $k_{non\text{-}rad}$, respectively) of the samples can be further calculated by the following equations:

$$\frac{1}{\tau_{av}} = k_{rad} + k_{non-rad} \tag{1}$$

$$\text{PLQY} = \frac{k_{rad}}{k_{rad}+k_{non-rad}} \tag{2}$$

The continuous decrease in the absolute values of the k_{rad} and $k_{non\text{-}rad}$ is mainly due to the promotion of the carrier lifetime by an order of magnitude. However, the ratio of k_{rad} to $k_{non\text{-}rad}$ is gradually increased, which means more excited carriers in $CdAc_2$ passivated films tend to be radiative recombined (**Supplementary Table 1**). In a conclusion, it is obvious that with the synergistic passivation effects of Cd^{2+} and Ac^-, the quality of perovskite films was improved significantly.

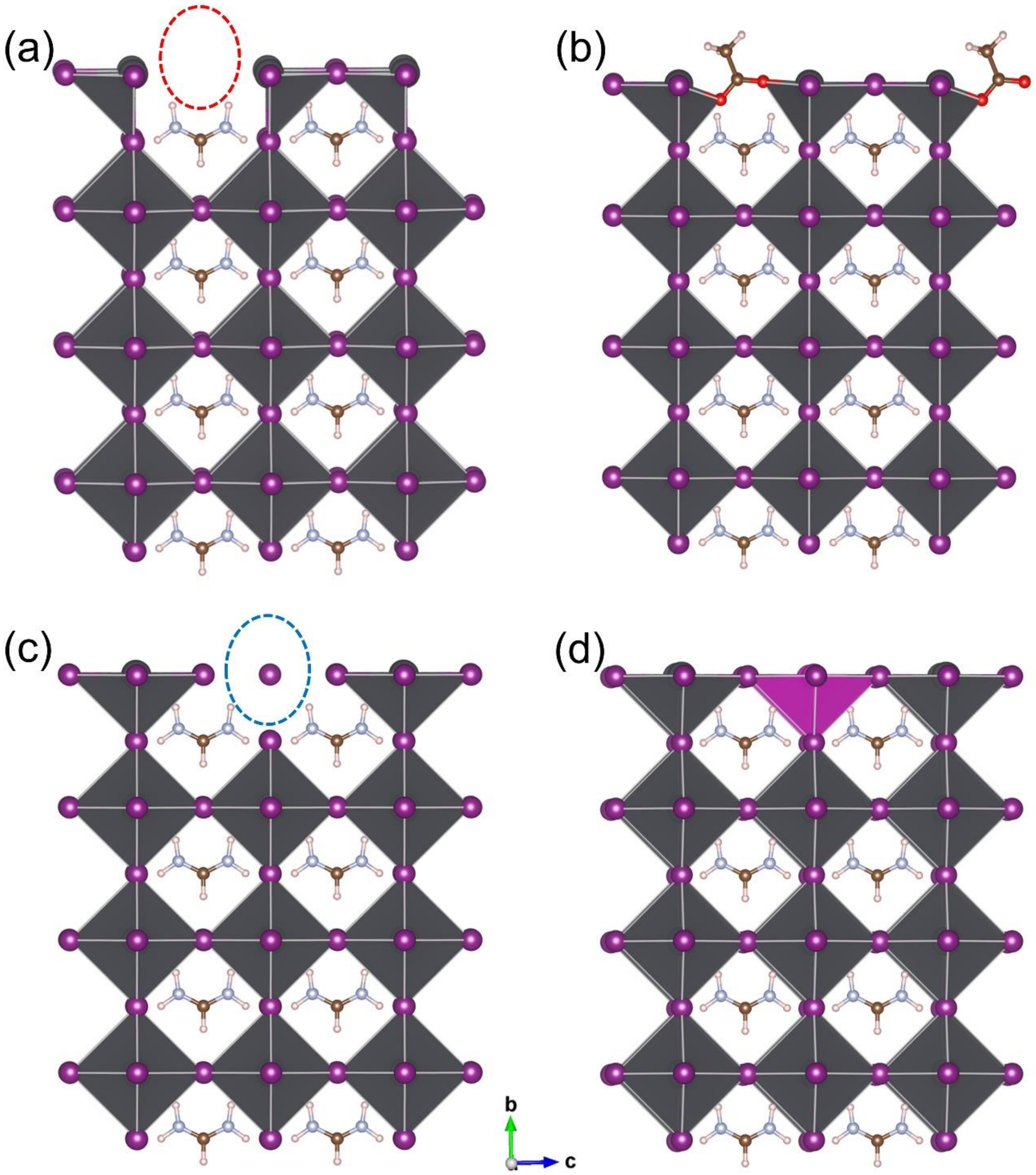

Supplementary Figure 8. The model for the combination of Cd^{2+}, Ac^{-}, and perovskite grains. (a) Schematic of the crystals with iodine vacancies (V_I) on the surface; **(b)** Ac^{-} molecules compensate V_I and coordinate with perovskite grains; **(c)** Schematic of the crystals with lead vacancies (V_{Pb}) on the surface; **(d)** Cd fill the V_{Pb} and bind to the surrounding iodine. The red circle represents the V_I, and the blue circle represents the V_{Pb}.

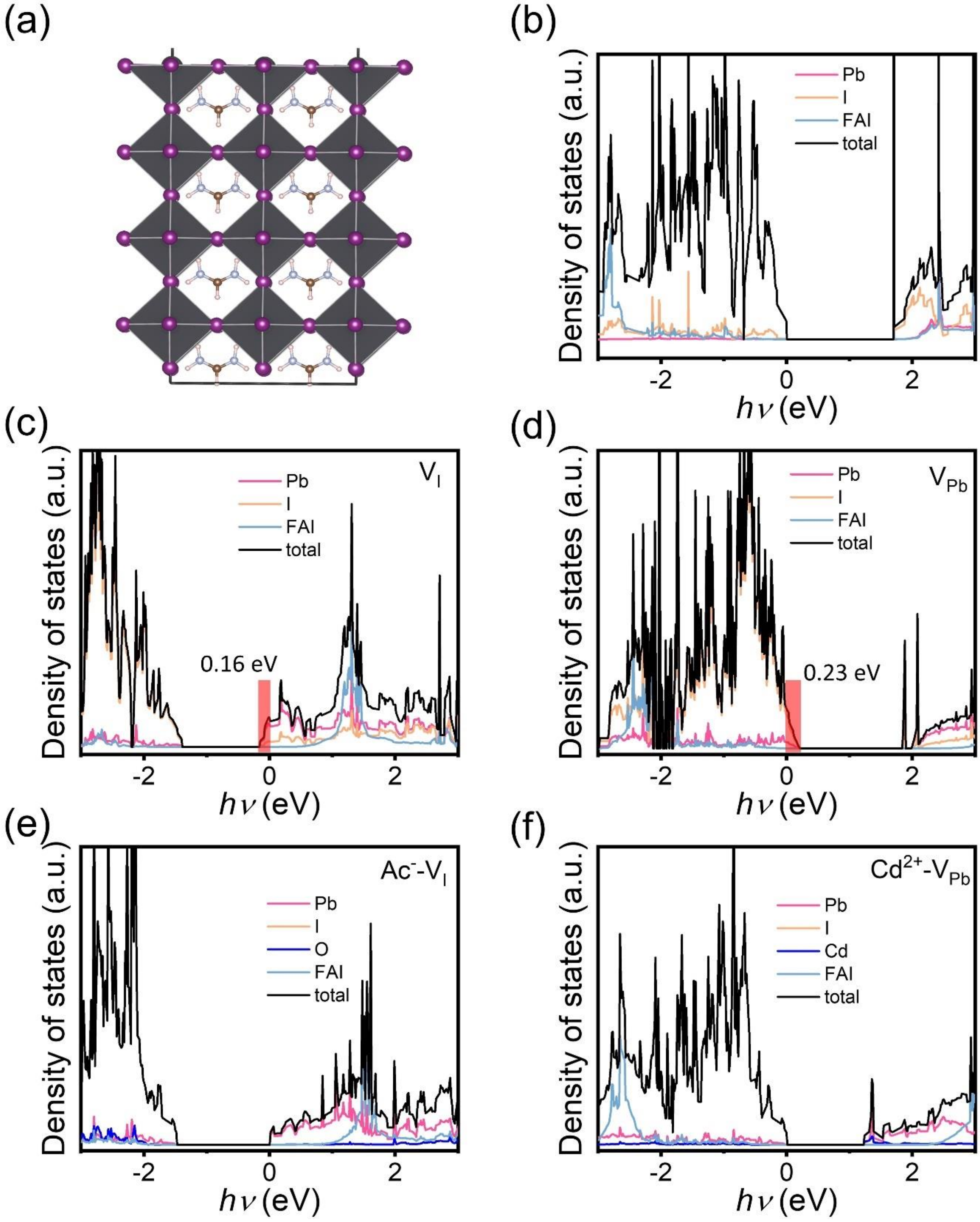

Supplementary Figure 9. Density of states (DOS) calculations. (a) Perfect crystal structure and **(b)** DOS of $FAPbI_3$ 100 slabs without any defects; DOS of $FAPbI_3$ 100 slabs with **(c)** V_I and **(d)** V_{Pb} on the grain surface. DOS of $FAPbI_3$ 100 slabs, when **(e)**Ac^- passivated the V_I and **(f)** Cd^{2+} interacted with V_{Pb}.

We calculated the two typical defects on the surface edges of $FAPbI_3$ crystals, including surface V_{Pb} and V_I. The calculations suggest that surface V_I can result in a series of band-edge states under the conduction band minimum (E_{CBM}), while V_{Pb} can lead to a series of band-edge states upon the valence band maximum (E_{VBM}).

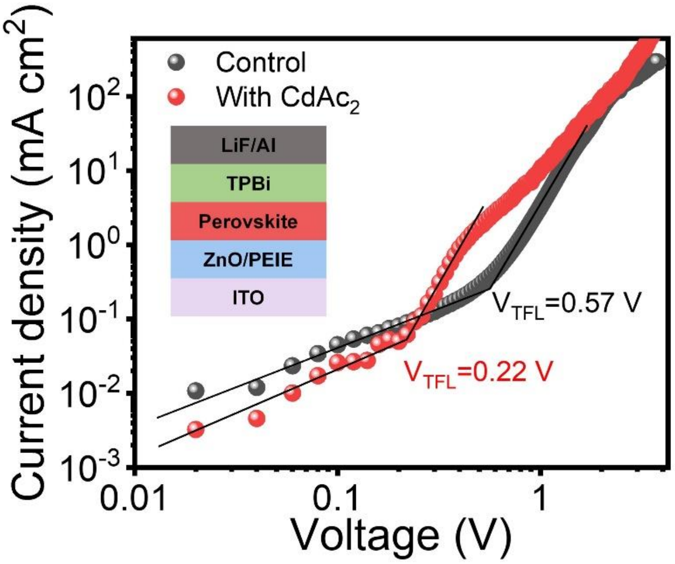

Supplementary Figure 10. SCLC measurements of electron-only devices. For the device with $CdAc_2$ incorporation, the trap-filled limited voltage (V_{TFL}) decreased from 0.57 to 0.22 V, and the density of electron traps decreased from around 9.5×10^{17} cm^{-3} to 3.6×10^{17} cm^{-3}, which is about one-third of the control one.

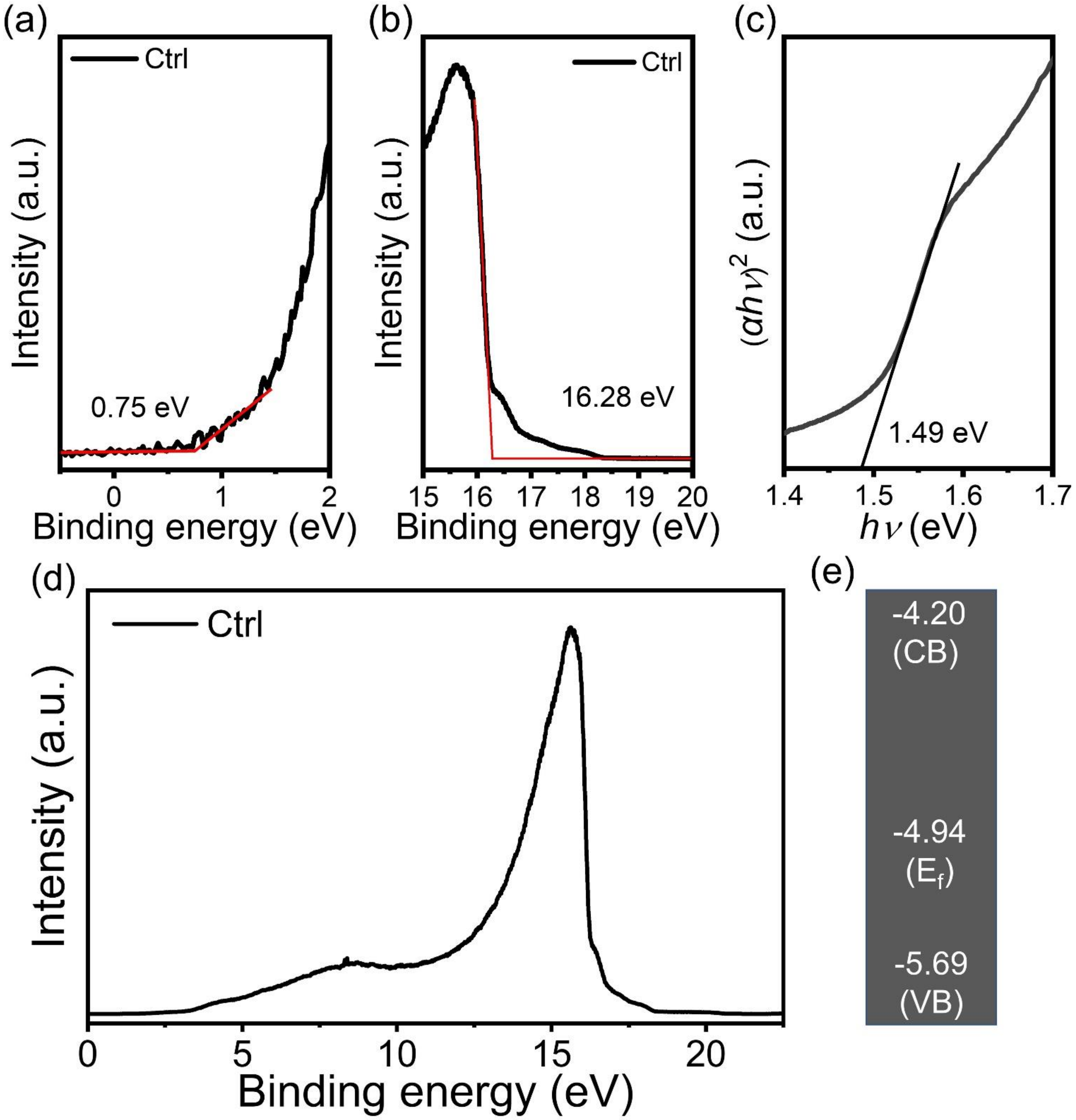

Supplementary Figure 11. Energy levels of original perovskite film. (a) The secondary photoelectron cut-off, **(b)** the ionization edge, **(c)** the Tauc plot, **(d)** UPS spectrum, and the **(e)** calculated energy level positions of an original perovskite film without any PMs. Note that the perovskite film was deposited on an ultra-thin ZnO substrate.

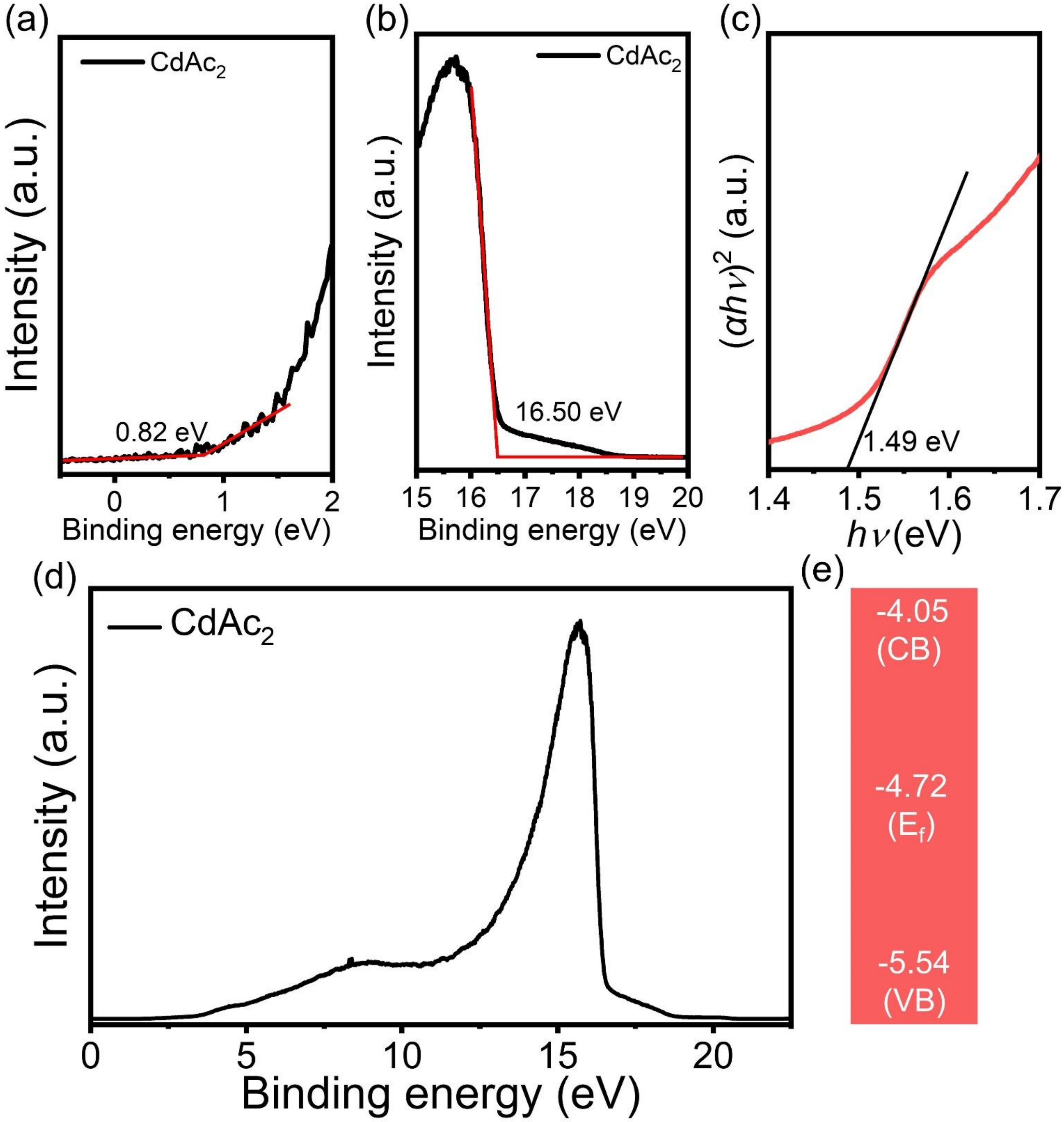

Supplementary Figure 12. Energy levels of perovskite film with $CdAc_2$ additive. (a) The secondary photoelectron cut-off, **(b)** the ionization edge, **(c)** the Tauc plot, **(d)** UPS spectrum, and the **(e)** the calculated energy level positions of a perovskite film with $CdAc_2$ additive. Note that the perovskite film was deposited on an ultra-thin ZnO substrate.

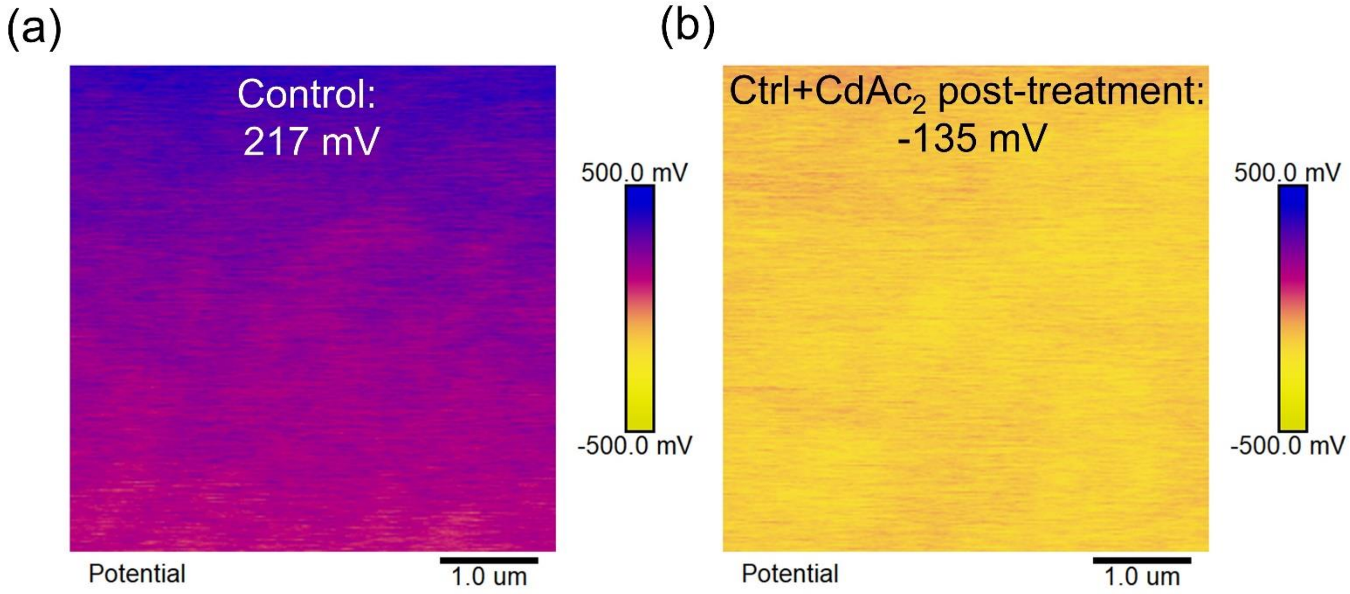

Supplementary Figure 13. KPFM measurements of perovskite films. (a) The result of a perovskite film without any PMs. **(b)** The result of a perovskite film without any PMs and with additional $CdAc_2$ post-treatment.

In the KPFM measurements, voltages (V) were applied to the samples, so the contact potential difference (CPD) was consistent with the work function of the samples. The CPD between the films and the tip could be calculated using the following formula: $V_{CPD} = W_{film}/e - W_{Tip}/e$, $E_f(\text{film}) = -W_{film}$, where W_{tip} and W_{film} are the work function of the tip and the as-obtained film, and $E_f(\text{film})$ is the Fermi level of the as-obtained film, respectively. When the V_{CPD} as well as W_{CPD} decreased, the E_f of the film would uplift.

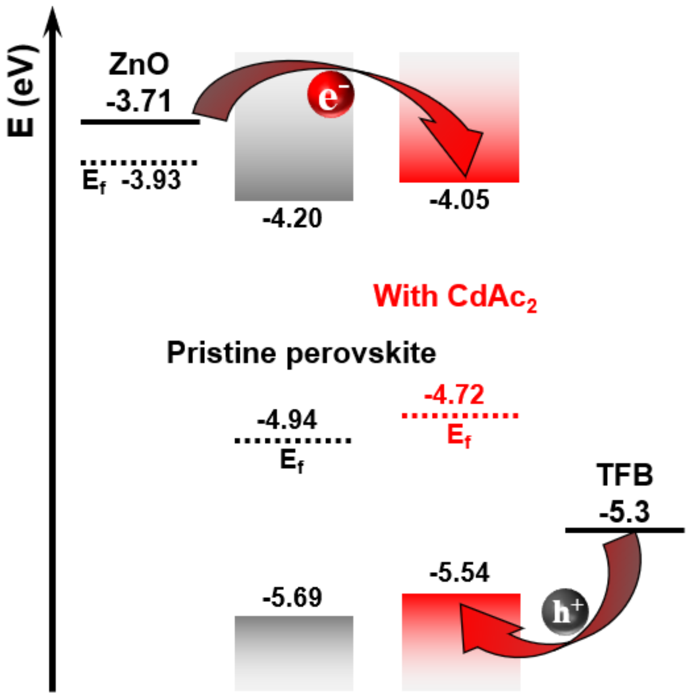

Supplementary Figure 14. Band structure of the devices with and without CdAc$_2$ additive. The Fermi level liftup from -4.19 to -4.72 eV, and the conduction band and valence band were raised by 0.15 eV, which greatly facilitated the injection of electrons and holes.

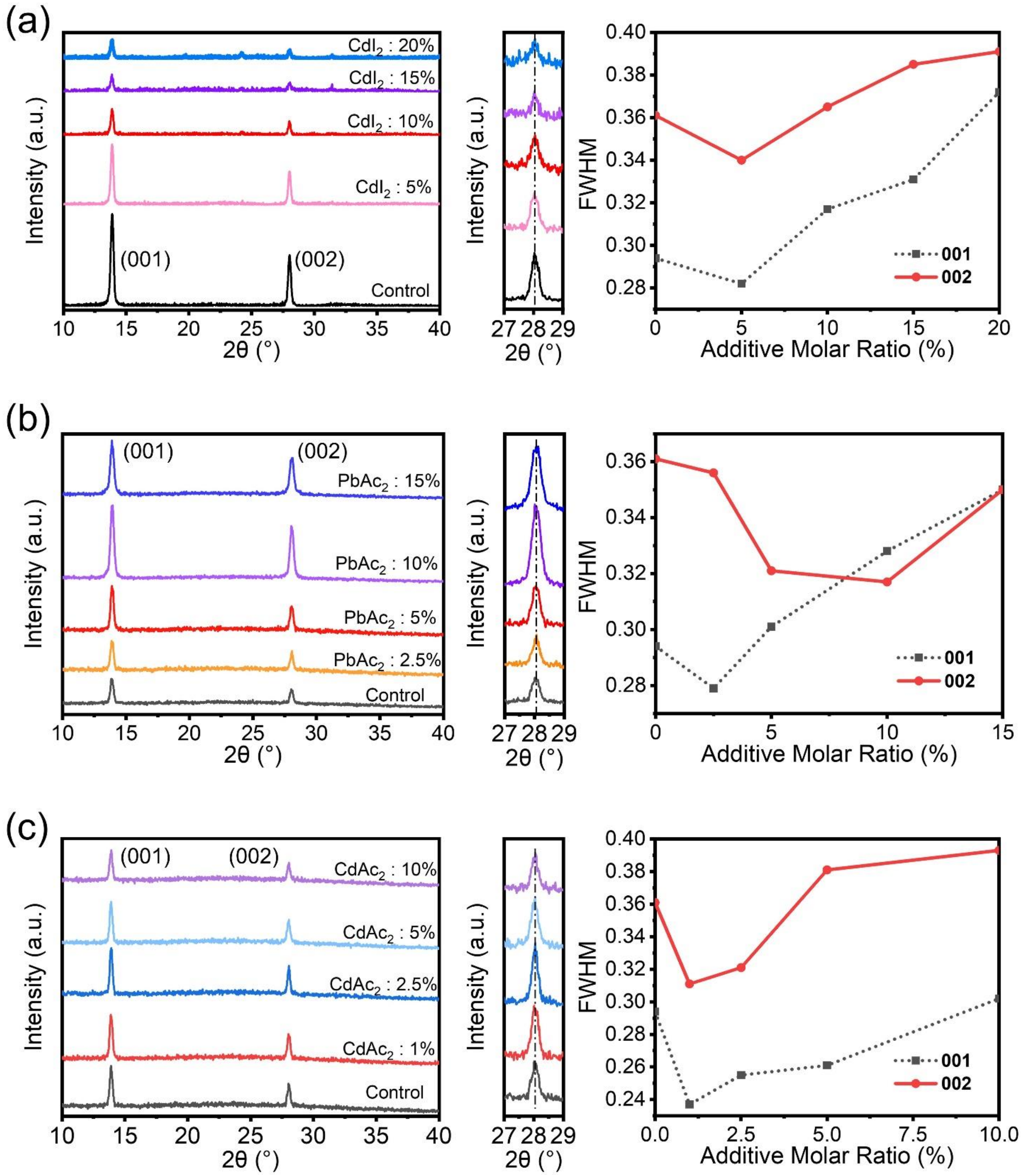

Supplementary Figure 15. XRD patterns of the $FAPbI_3$ films with different amounts of PMs. The comparison of intensity and peak position as well as full width at half maximum (FWHM) statistics in perovskite samples with different amounts of **(a)** CdI_2, **(b)** $PbAc_2$, and **(c)** $CdAc_2$ additives.

Note that all the one-dimensional (1D) XRD patterns were obtained from the 2D images. The peak position located at 28.0°, associated with the (002) peak, did not show any shift with the addition of additives. While the FWHM values of both the (001) and (002) characteristic peaks exhibited obvious changes after the addition of additives. The reduced FWHM indicated the improved crystal quality of $FAPbI_3$ under Cd^{2+} and Ac^{2-} effects.

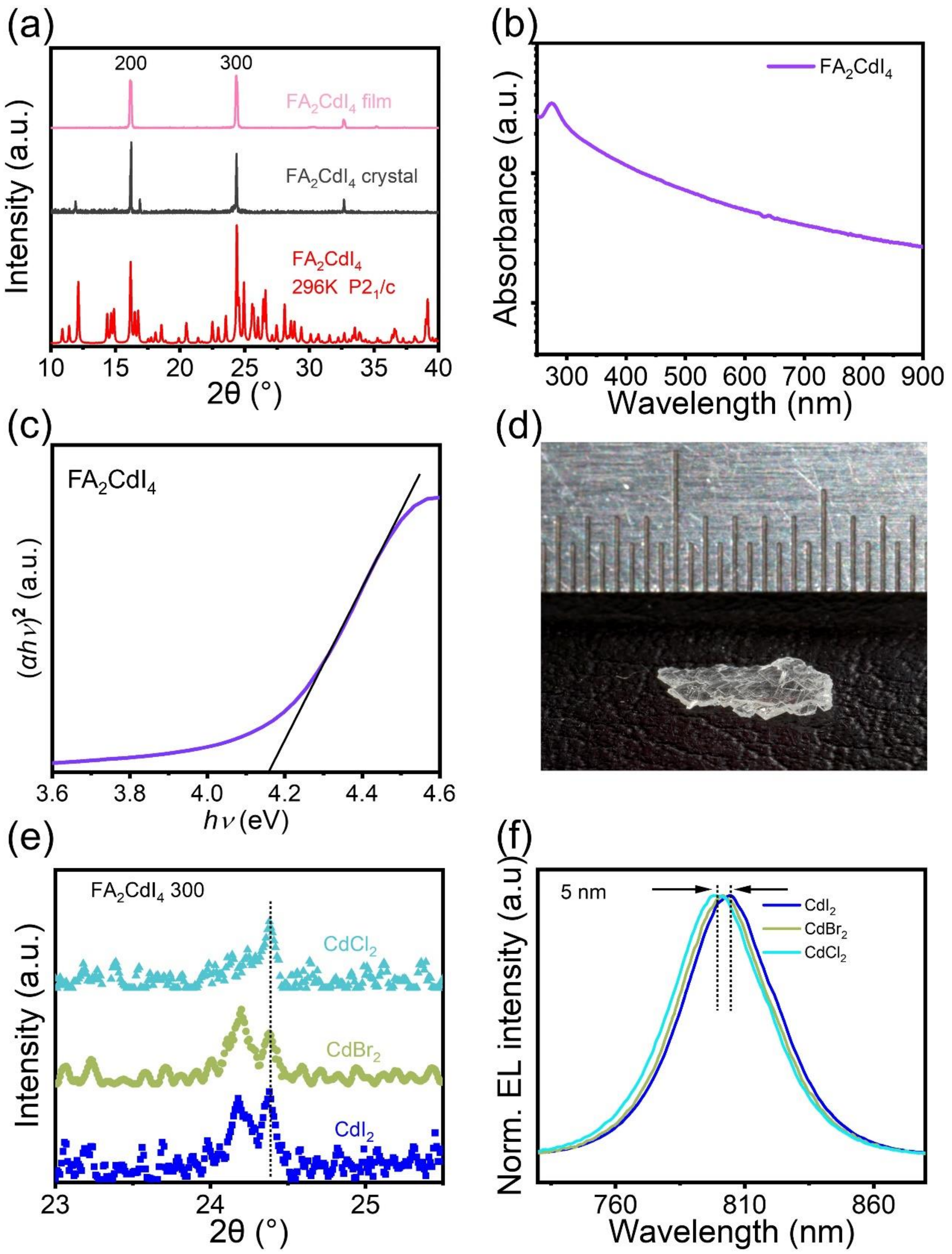

Supplementary Figure 16. Characterization of 0D FA_2CdI_4 perovskitoid. (a) The measured XRD patterns of a FA_2CdI_4 crystal, a stoichiometric FA_2CdI_4 thin film, and simulated XRD pattern for FA_2CdI_4 perovskitoid at 296 K. Two main peaks were observed at 16.18° and 24.38°, which corresponds to (200) and (300) planes of 0D FA_2CdI_4 perovskitoid, respectively. **(b)** Absorption spectrum of a FA_2CdI_4 thin film. **(c)** Tauc plot of a FA_2CdI_4 film. **(d)** Photograph of a 0D FA_2CdI_4 crystal.

In the XRD patterns of the thin films, the proportion of additives was 20 mol%. The appearance of the 23.38° diffraction peak proved the formation of 0D FA_2CdI_4 perovskitoid. The 5 nm shift of the EL spectrum at 2.5 V was due to the increased bandgap, which was caused by the entry of Br- and Cl- into the interior of the perovskite lattice.

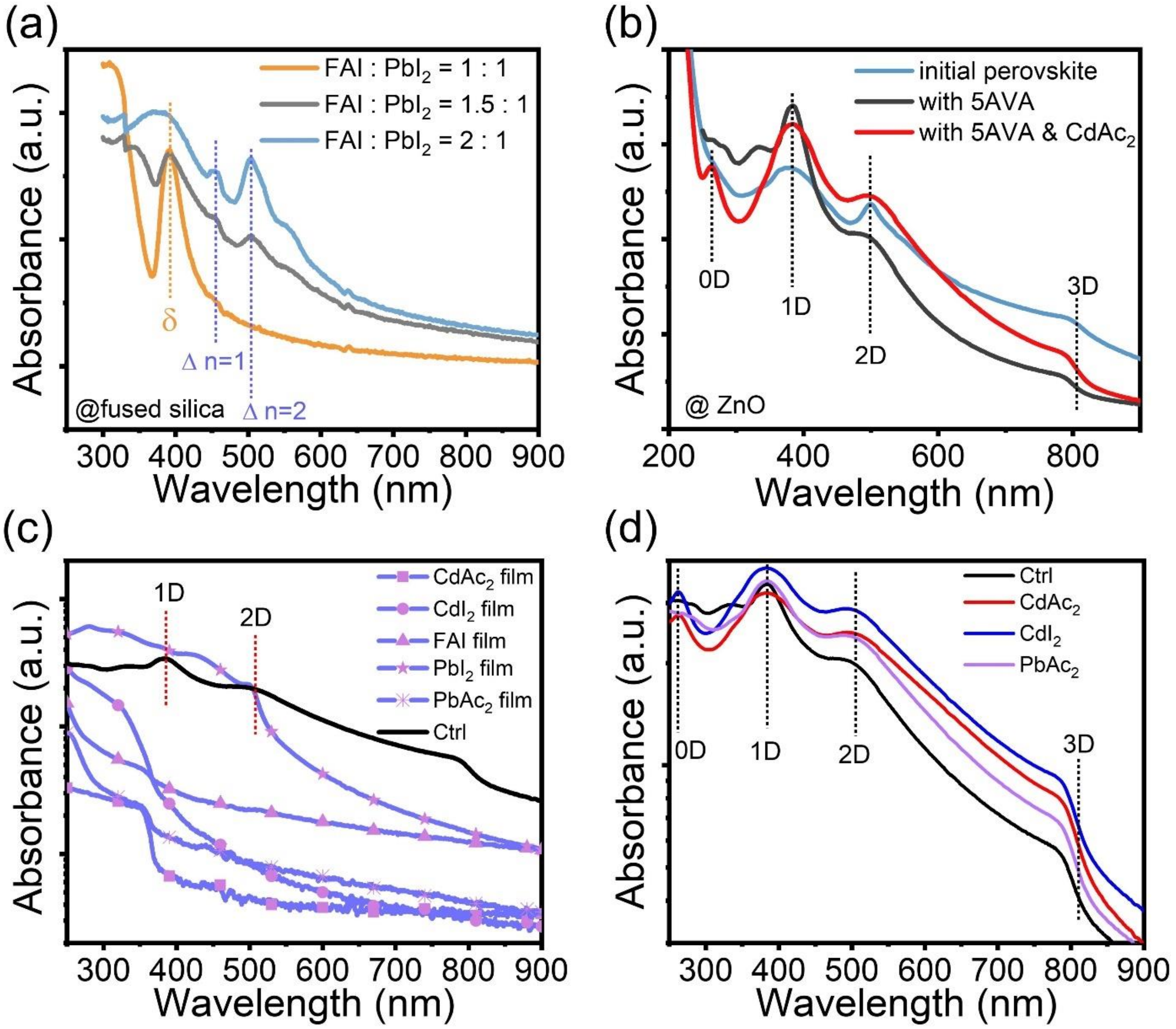

Supplementary Figure 17. Characterization of low dimensionality. (a) Absorption spectra of $FAPbI_3$ films with different mole ratios of FAI and PbI_2 on fused silica substrates. **(b)** Absorption spectra of $FAPbI_3$ films with different additives on ZnO substrates. Here the initial perovskite only contains FAI and PbI_2, and the ratio of FAI to PbI_2 is 2:1. **(c)** Absorption spectra of various initial components and the perovskite films in the control group. **(d)** Absorption spectra of perovskite films with different PMs.

More low-dimensional information can be displayed on fused silica substrates. Different proportions of FAI in the precursor solutions caused an obvious change in the absorption spectra in the range of 260-550 nm. The three shoulder peaks at around 400, 460, and 500 nm are associated with Pb-based low-dimensional phases[2]. The film exhibits one-dimensional δ-phase exciton absorption when there is no overdose of FAI. In the presence of excess FAI in the precursors, the films exhibited 2D $FA_2FA_{n-1}Pb_nI_{3n+1}$ phase exciton absorption. The ZnO substrates played an important role in the formation of α-phase $FAPbI_3$, while the 5AVA seemed not to affect this process. The absorption spectra of perovskite films containing different PMs remained the characteristics of the low-dimensional phases. For comparison, all the films containing Cd^{2+} emerged with a new excitonic absorption peak at around 280-290 nm, which was associated with a 0D FA_2CdI_4 perovskitoid and had nothing to do with the initial component of perovskite.

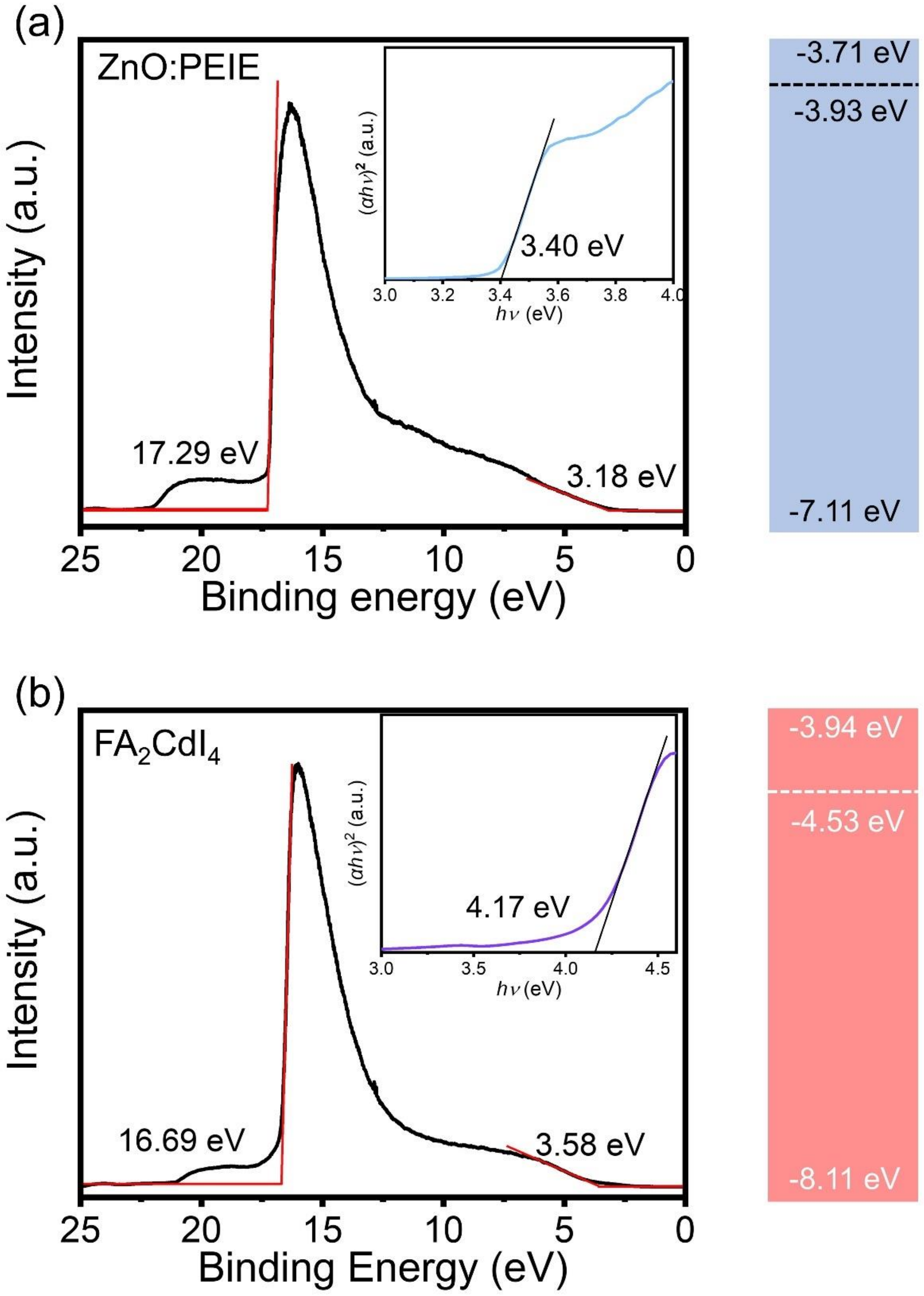

Supplementary Figure 18. UPS spectra of ZnO: PEIE and FA_2CdI_4. The secondary photoelectron cutoff, the ionization edges, the Tauc plots, and the calculated energy level positions of **(a)** a PEIE-modified ZnO film and **(b)** a FA_2CdI_4 film.

The results show that both ZnO and FA_2CdI_4 films are typical n-type semiconductors. The E_{CBM} of FA_2CdI_4 is 0.23 eV lower than that of ZnO, which is more conducive to the downstream flow of electrons.

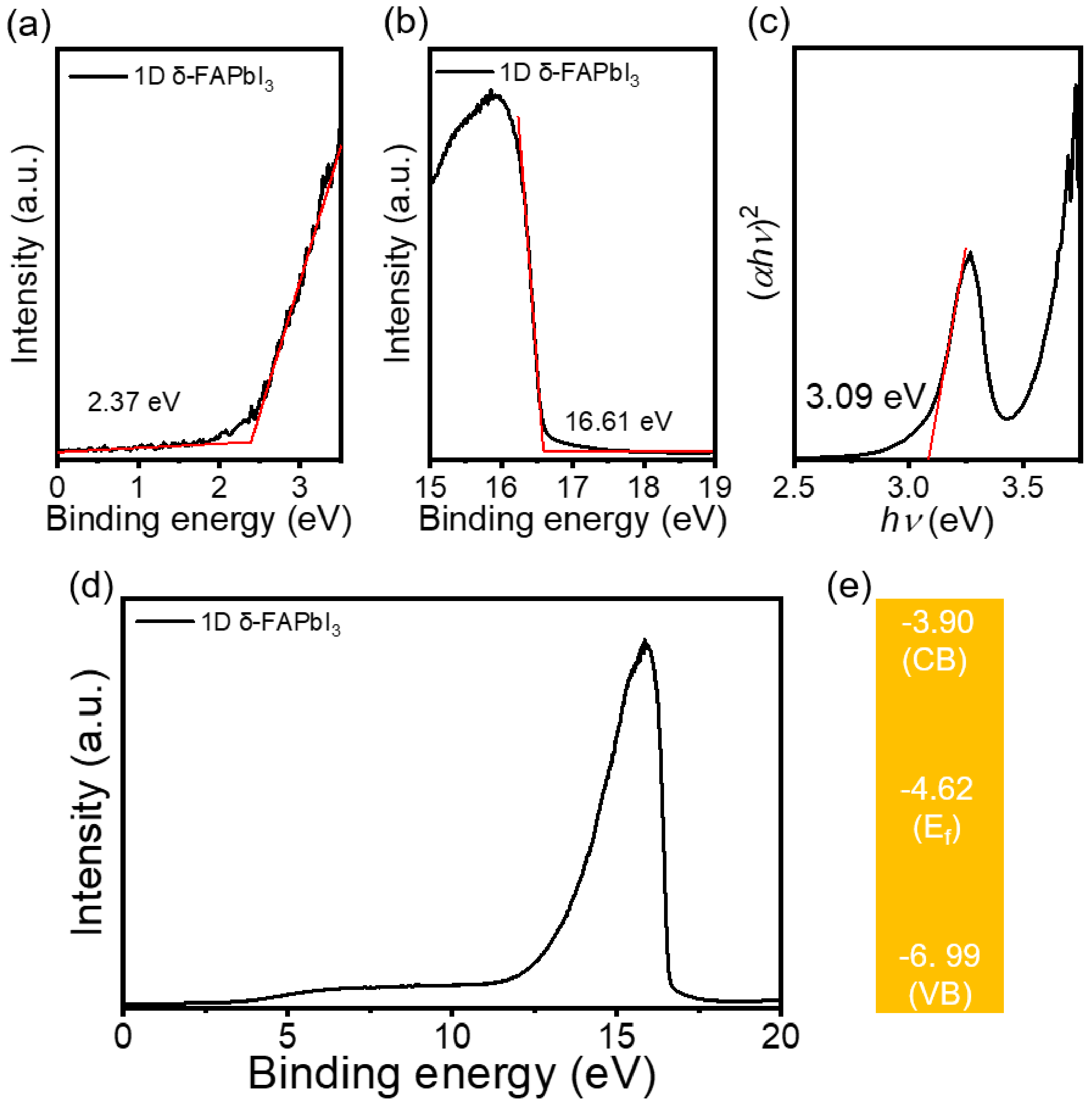

Supplementary Figure 19. Energy levels of 1D δ-phase $FAPbI_3$. (a) The secondary photoelectron cut-off, **(b)** the ionization edge, **(c)** the Tauc plot, **(d)** UPS spectrum, and the **(e)** calculated energy level positions of 1D δ-phase $FAPbI_3$. Note that the perovskite film was deposited on an ultra-thin ZnO substrate.

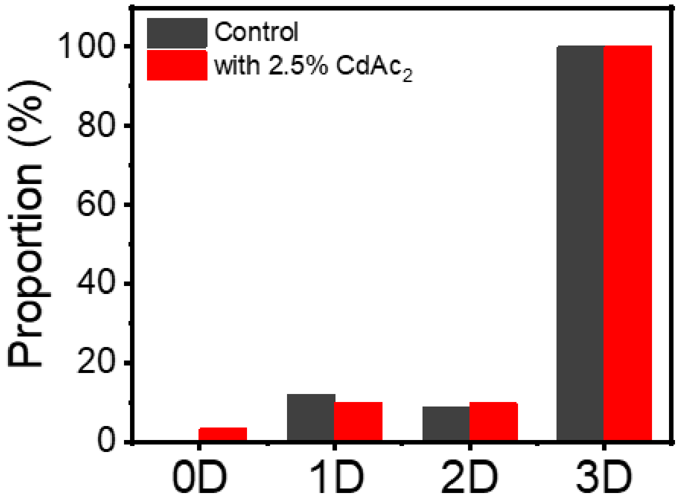

Supplementary Figure 20. Content distribution of different dimensional phases. The ratio of multi-dimensional perovskites (0D: 1D: 2D: 3D) in control group was 0: 12.0: 8.8: 100, and the result in perovskite film with 2.5% $CdAc_2$ was 3.3: 10: 10.1: 100.

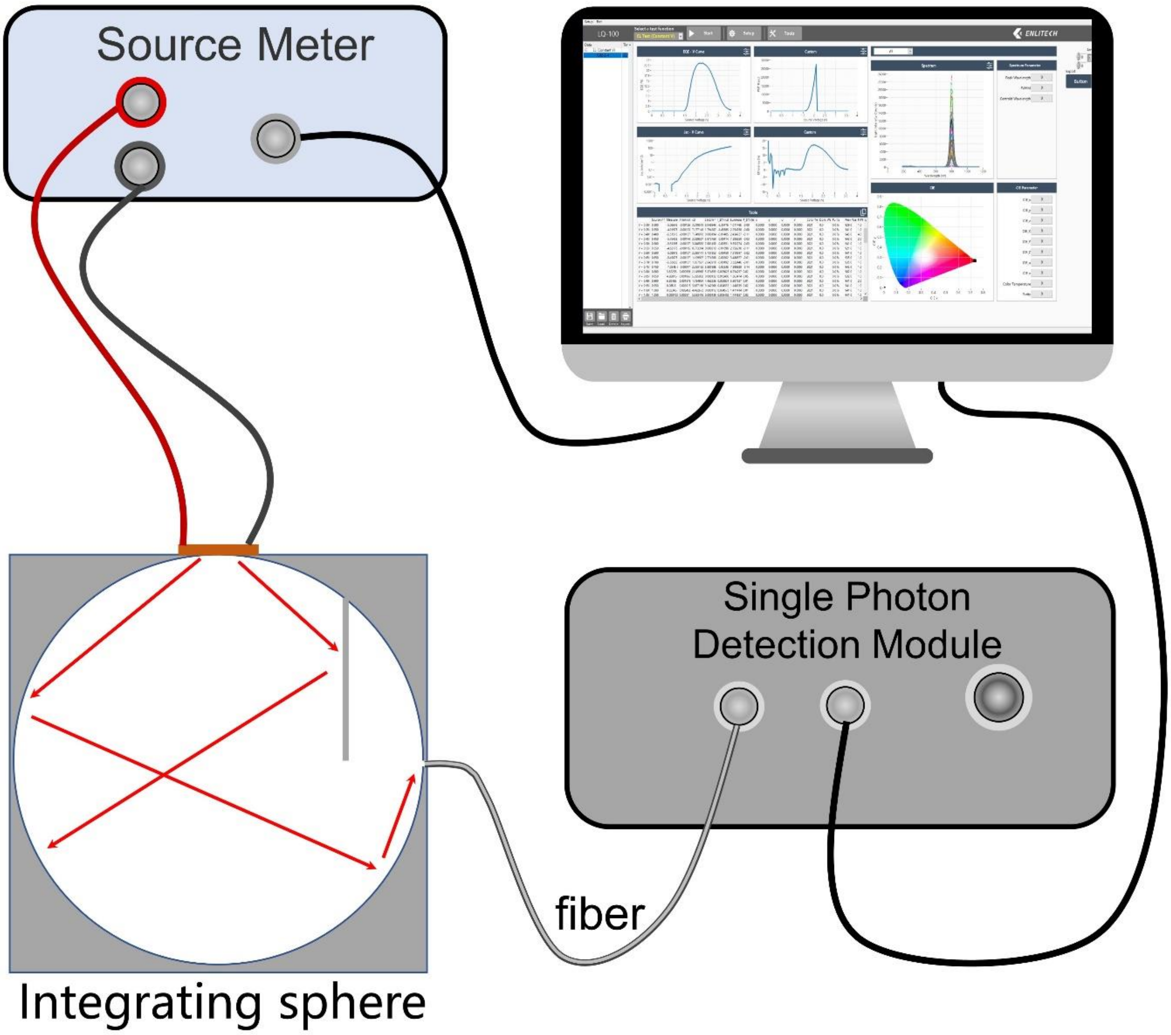

Supplementary Figure 21. Schematic layout of PeLED measurement setup. The design of a small light inlet hole and a light baffle improved the testing accuracy, which followed the setup reported in the literature[3].

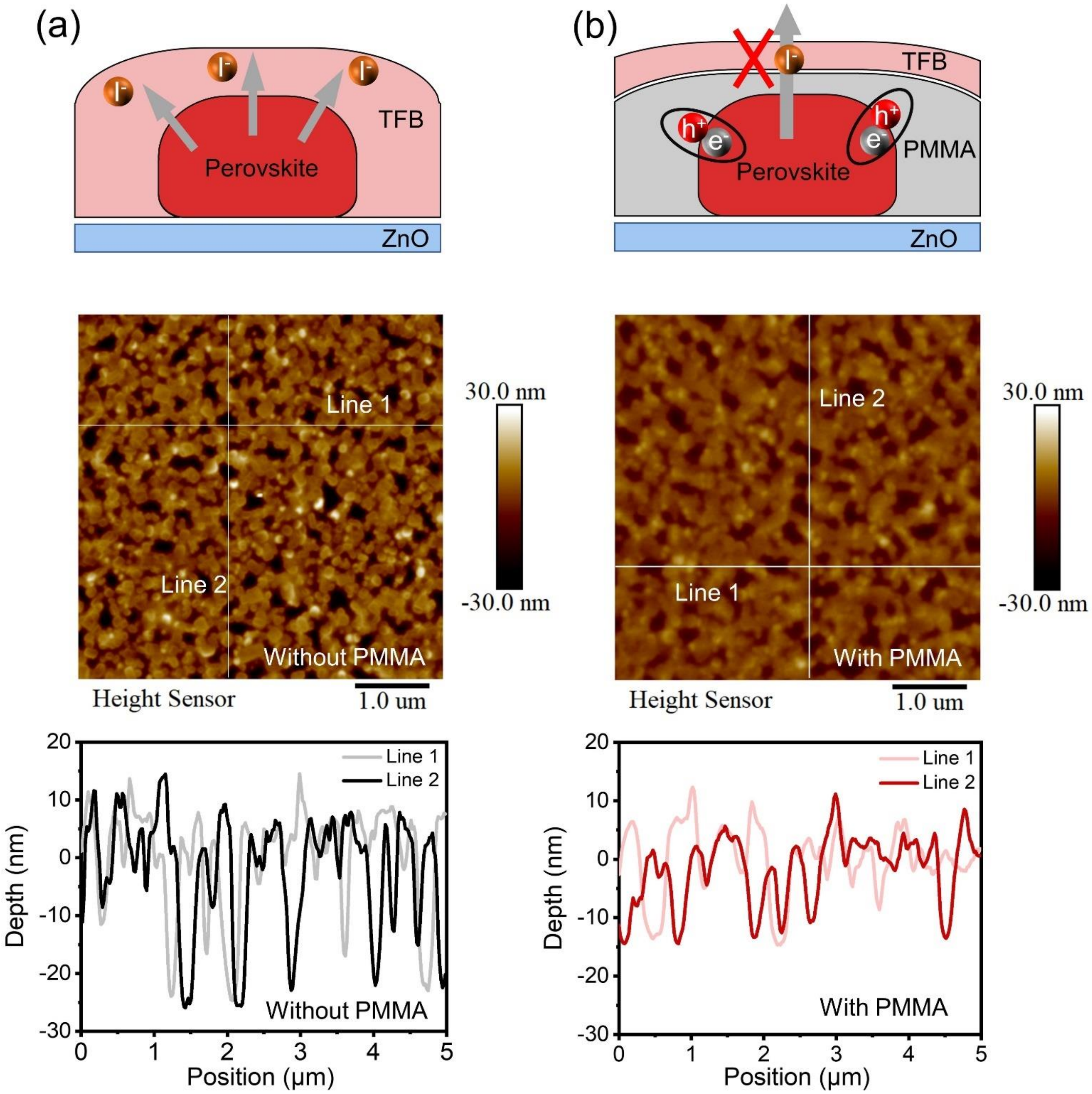

Supplementary Figure 22. Schematic diagrams of electron-hole injection and the morphology of perovskite films with and without PMMA layer. Schematic diagrams of iodide ion migration, carrier recombination, and AFM images of perovskite grains (a) without and **(b)** with PMMA.

The introduction of the PMMA insulating layer can effectively confine the carriers at the top interface of the perovskite, increasing the local carrier density and thus increasing the efficiency as reported in the literature[4]. In addition, the introduction of PMMA also blocked the diffusion of iodide ions to the metal electrode. Moreover, the introduction of PMMA did not change the coupling structure of the thin films. The insulating layer was also used to block the migration channels of iodide ions to TFB and electrode layers.

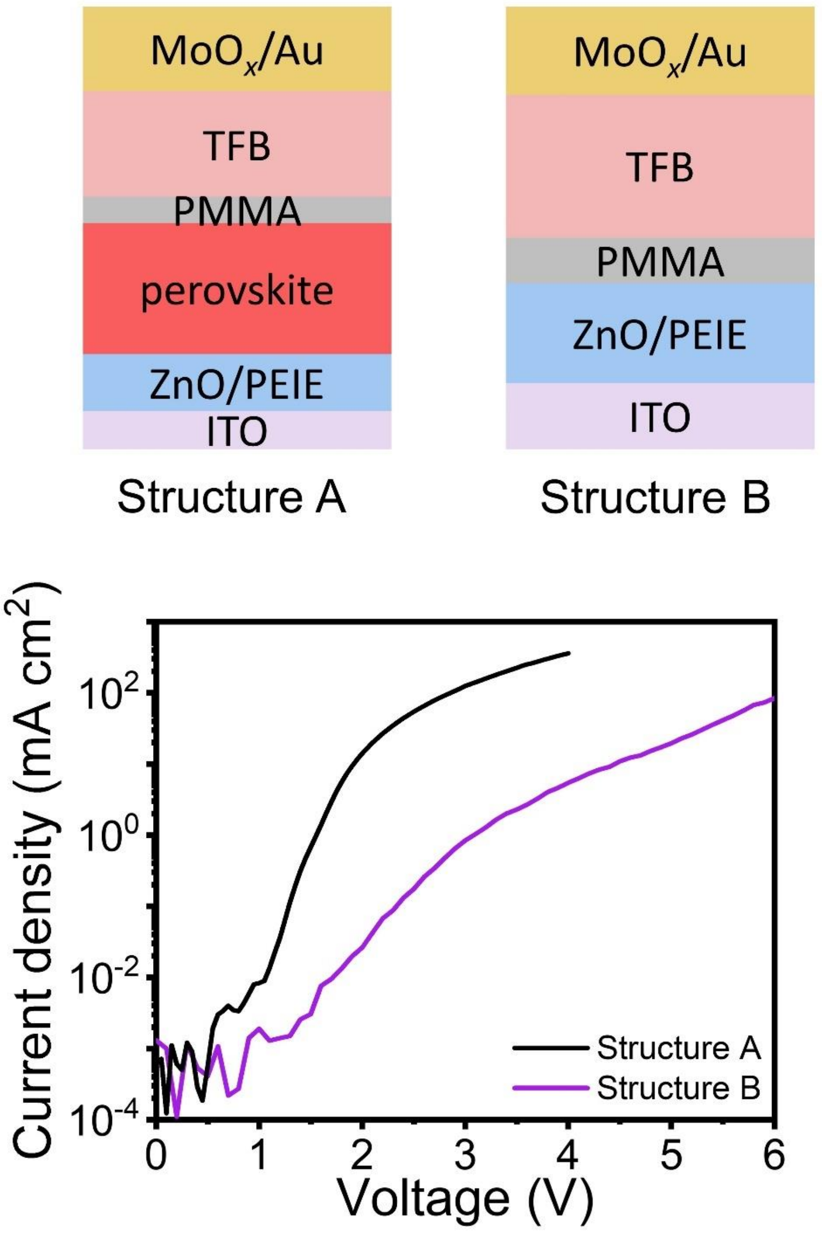

Supplementary Figure 23. Current densities of the devices using different structures. Two different structures of devices were used to observe the regularity of the current change (Structure A: pristine device. Structure B: without perovskite). The current density of the perovskite-free device is two orders of magnitude lower than that of the complete LED device, indicating that the separated grains with visible boundaries did not result in higher leakage currents.

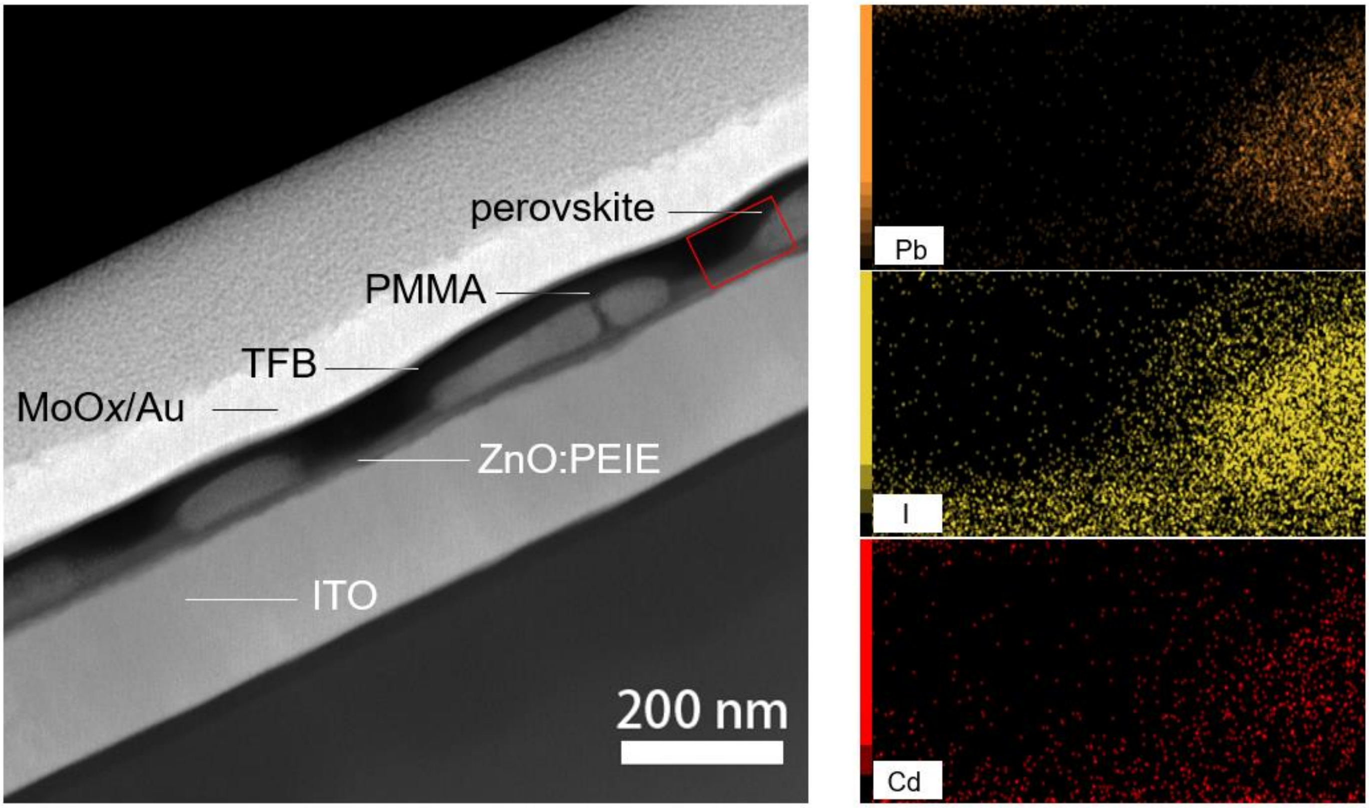

Supplementary Figure 24. HAADF-STEM and selected area element mapping images of a representative device.

HAADF-STEM image demonstrates the nano-island structure of perovskite films. The selected area element mapping shows the composition of perovskite lattice. In addition, there is a layer of cadmium-iodine complex at the bottom of the perovskite lattice, which might be the 0D FA_2CdI_4.

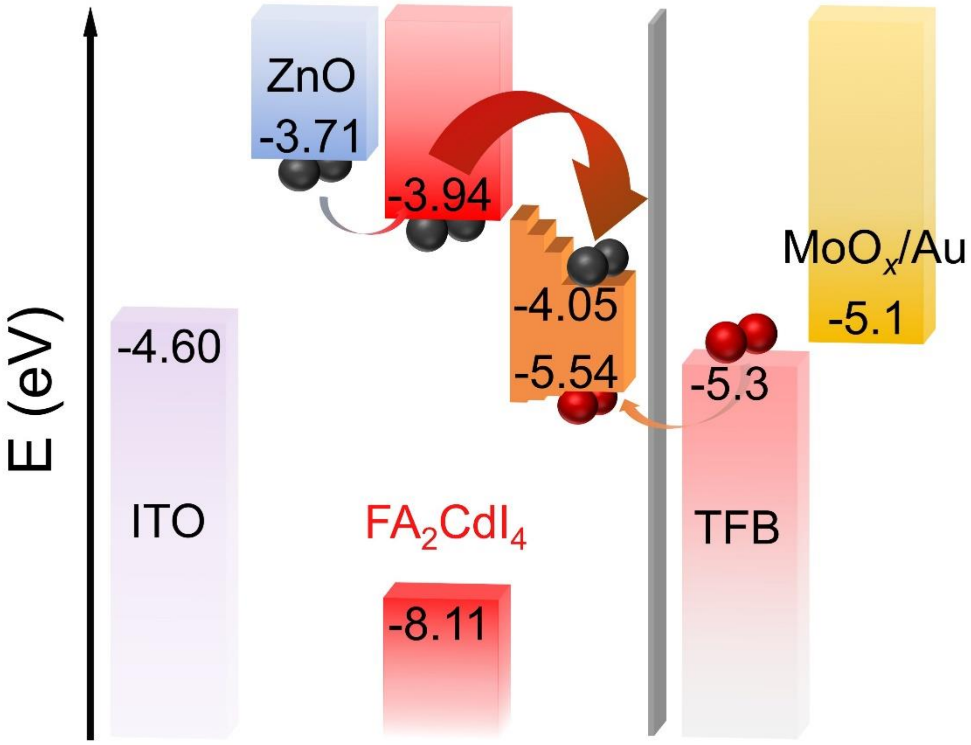

Supplementary Figure 25. Energy band structure of as-fabricated devices. The insertion of 0D FA_2CdI_4 perovskitoid shows smooth electron injection from ZnO to emitting layer, as well as the characteristics of stepped energy bands inside perovskite.

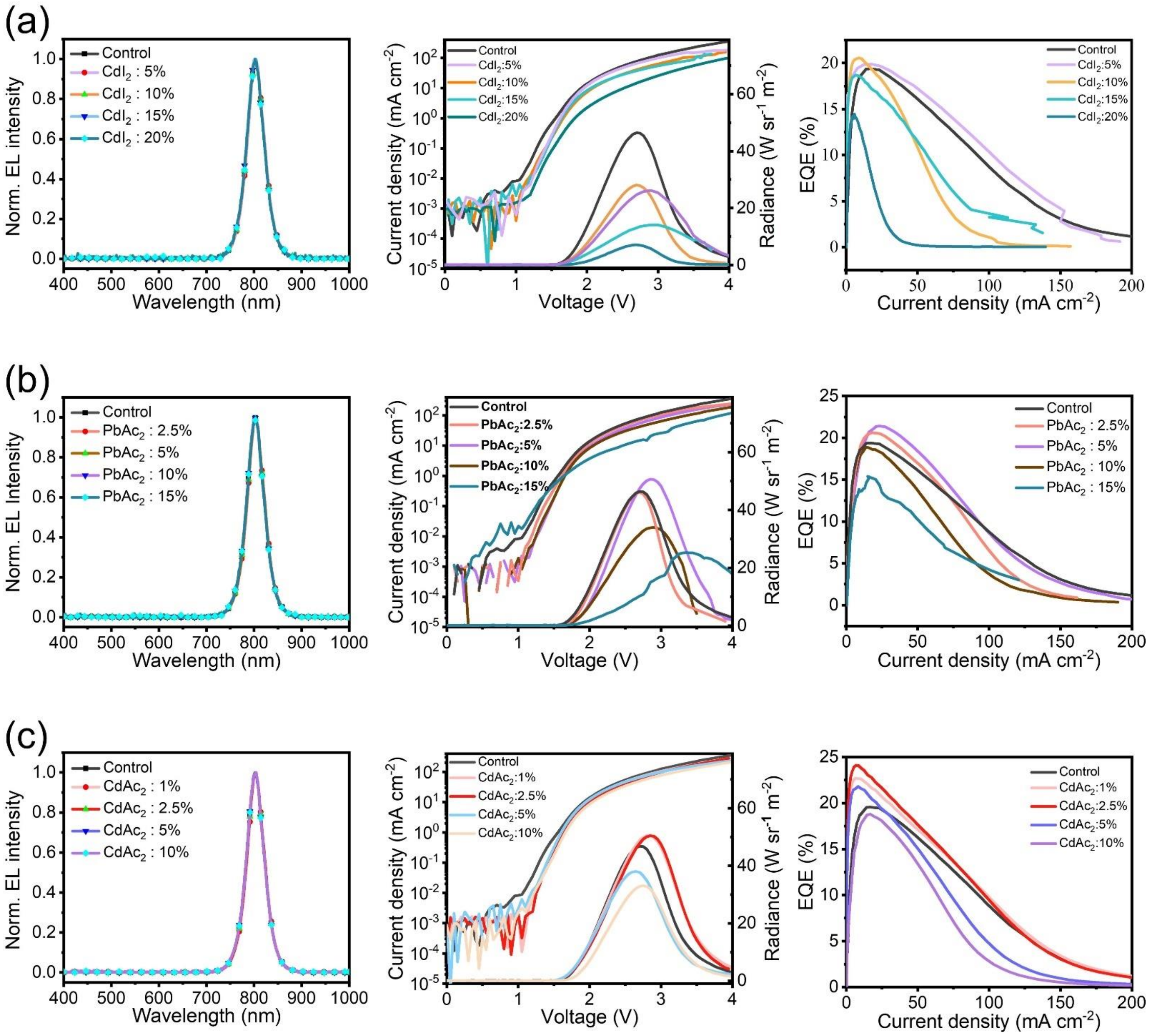

Supplementary Figure 26. Performance characteristics of representative devices based on different concentrations of PMs. EL spectra (at 2.2 V), *J-V-R* curves, EQE-*J* plots of devices based on different concentrations of **(a)** CdI_2, **(b)** $PbAc_2$, and **(c)** $CdAc_2$ additives. The perovskite layers were prepared from the precursors with molar ratios of FAI: PbI_2: 5AVA: PMs = 2: 1: 0.85 :x (x = 0 ~ 0.2). The concentration of Pb^{2+} was fixed at 0.075 M.

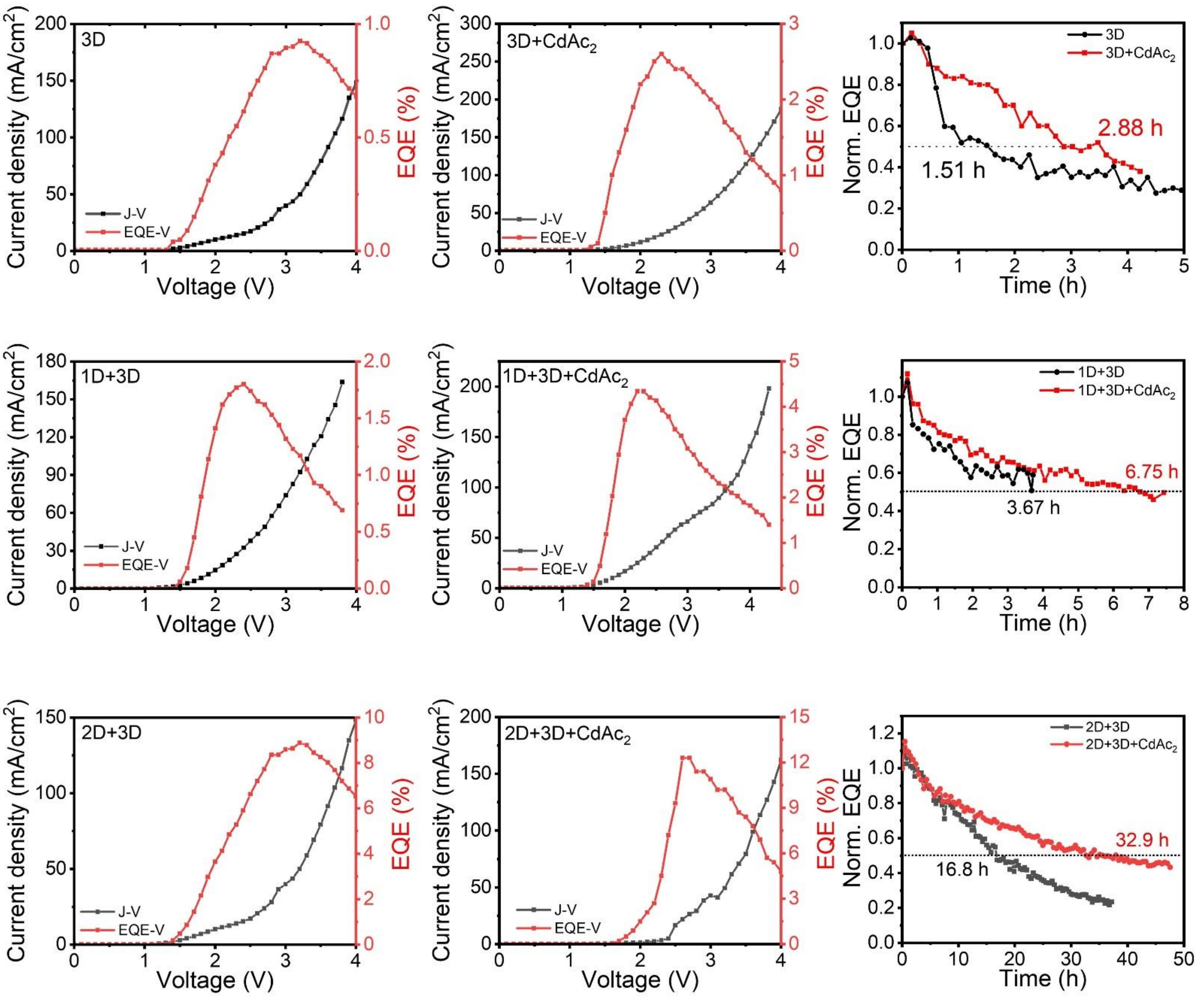

Supplementary Figure 27. Device Performance based on different dimensional phases. Note that the devices without encapsulation were measured in an N_2-filled glovebox.

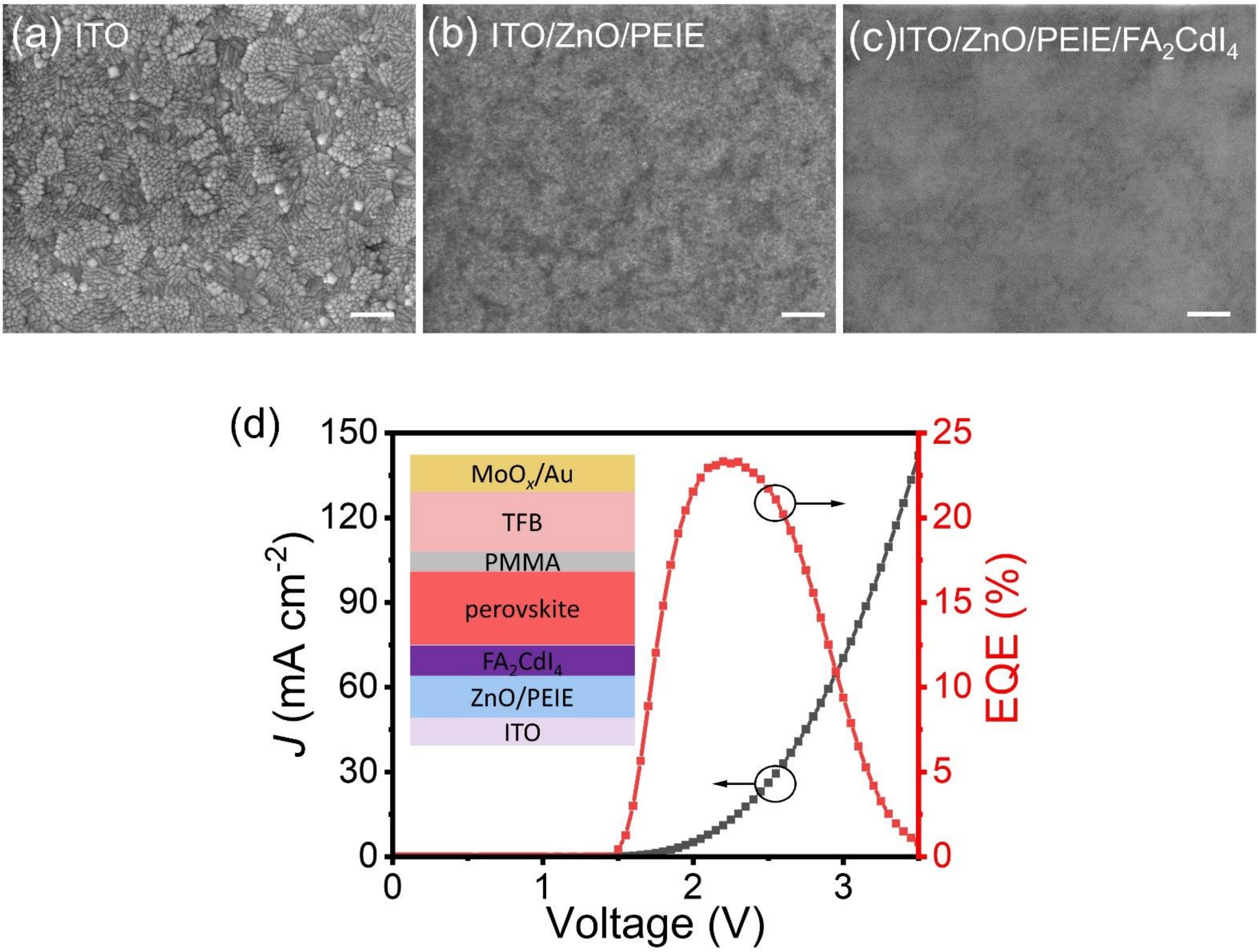

Supplementary Figure 28. Film morphology and peformance of the device with a 0D FA_2CdI_4 insert film. SEM images of **(a)** an ITO substrate, **(b)** a ZnO/ITO substrate, and **(c)** a FA_2CdI_4 film coated on a ZnO/ITO substrate. **(d)** EQE-*J*-*V* plot of a device with a pre-coated ultra-thin layer of FA_2CdI_4.

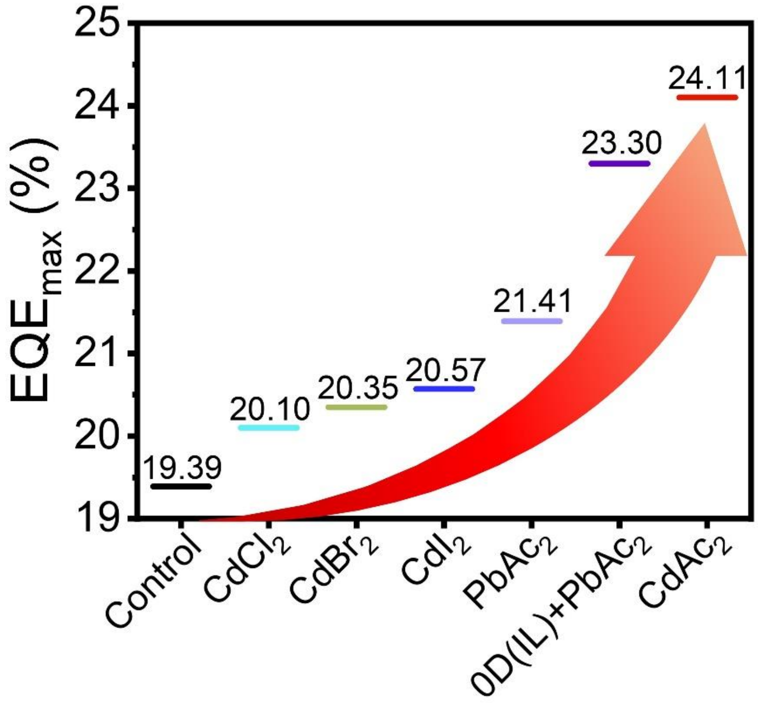

Supplementary Figure 29. Performance comparison of PeLEDs with different additives. As compared with the control group, the EQE improvement of the devices was attributed to the promotion of carrier transport by 0D materials and the modification of perovskite grains by Cd^{2+} and Ac^{-}.

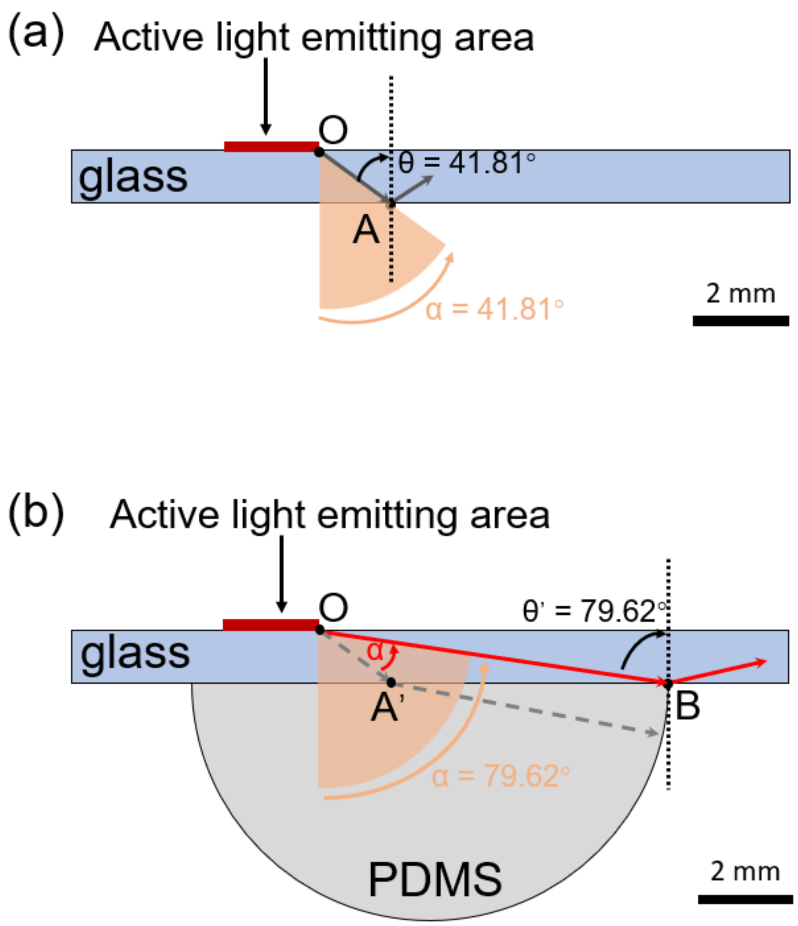

Supplementary Figure 30. Total reflection comparison of the devices. Light out-coupling design **(a)** without and **(b)** with PDMS half-sphere attachment on the PeLED substrate.

Take the light emitted from the right side of the point **O** as examples, the light is refracted from the glass to the air at the point **A**, and the total reflection angle is 41.81° when there is no PDMS hemisphere coverage. All emitted light within 41.81° of point O can be collected. However, the total reflection occurred at the edge of the hemisphere when the PDMS hemisphere is added, where the original total reflection point **A** has been changed to **B**. All emitted light within 79.62° of point O can be collected. The maximum coupling-out angle of a single light-emitting area is increased by more than 37°. Note that the refractive indices of glass, PDMS, and nitrogen are 1.5, 1.4, and 1, respectively.

(a)

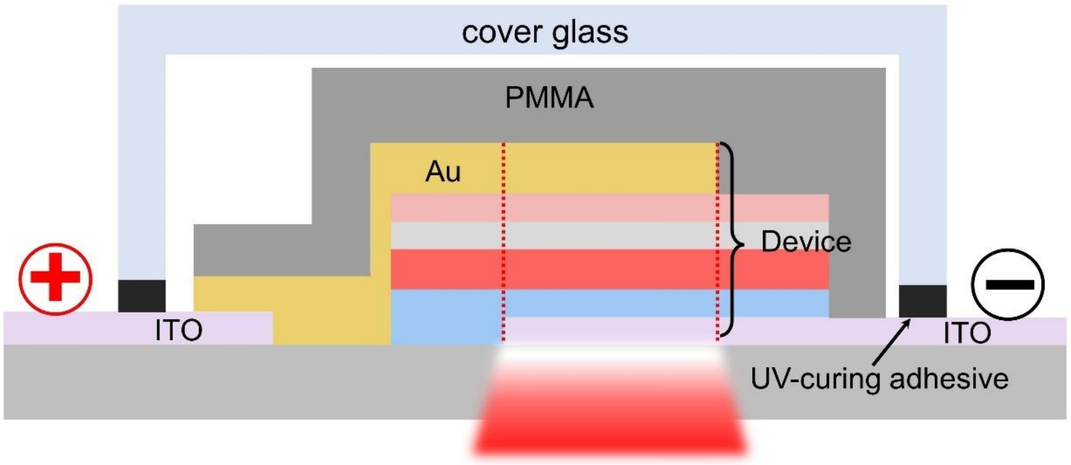

(b)

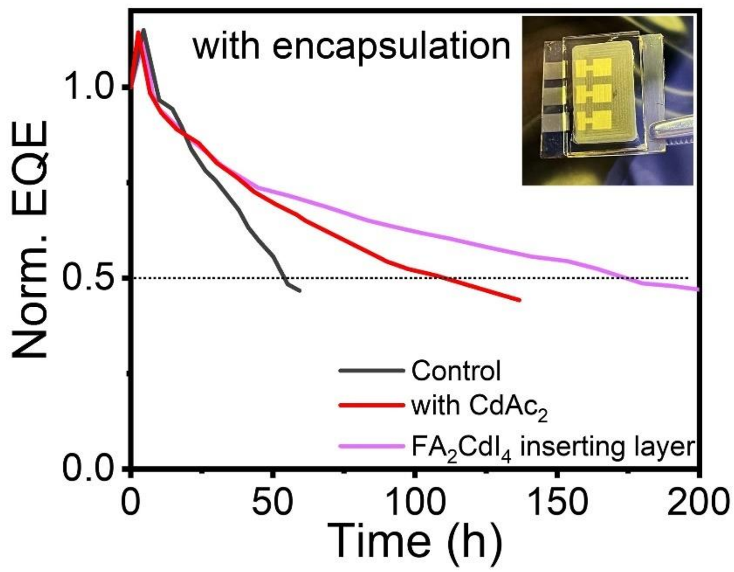

Supplementary Figure 31. Stability measurements of the encapsulated devices. (a) Schematic of encapsulation of PeLEDs with PMMA and cover glass. **(b)** Stability tests of different devices with encapsulation, which were measured in an N_2-filled glovebox.

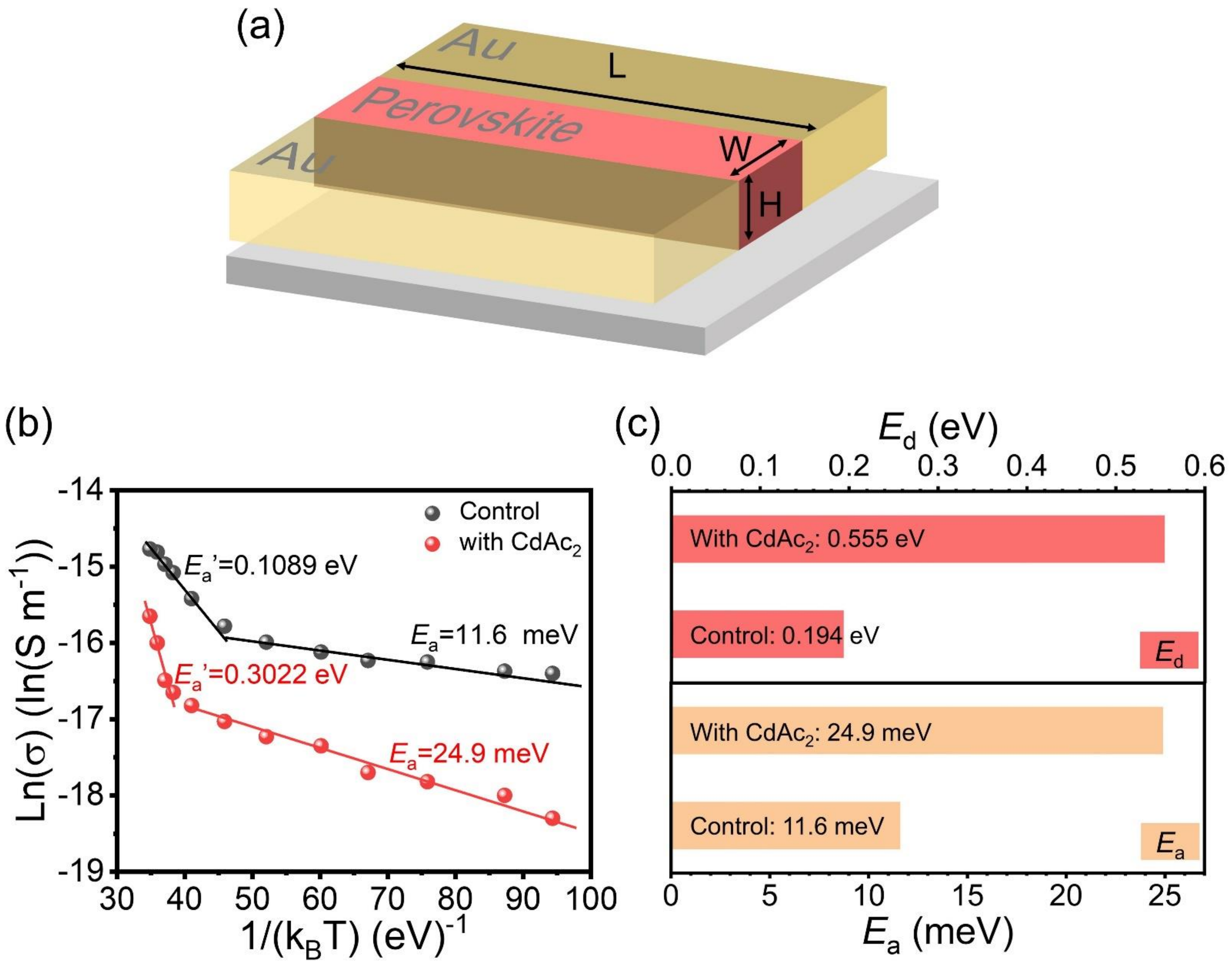

Supplementary Figure 32. Ion migration measurements and results. (a) Schematic diagram of conductivity measurements of lateral perovskites. **(b)** Arrhenius plots of the conductivity of perovskite films under a 10 V mm^{-1} electrical field at different temperatures. **(c)** Comparison of the ion-migration activation energy (E_a) and the formation energy (E_d) of movable ions in perovskite films with and without $CdAc_2$.

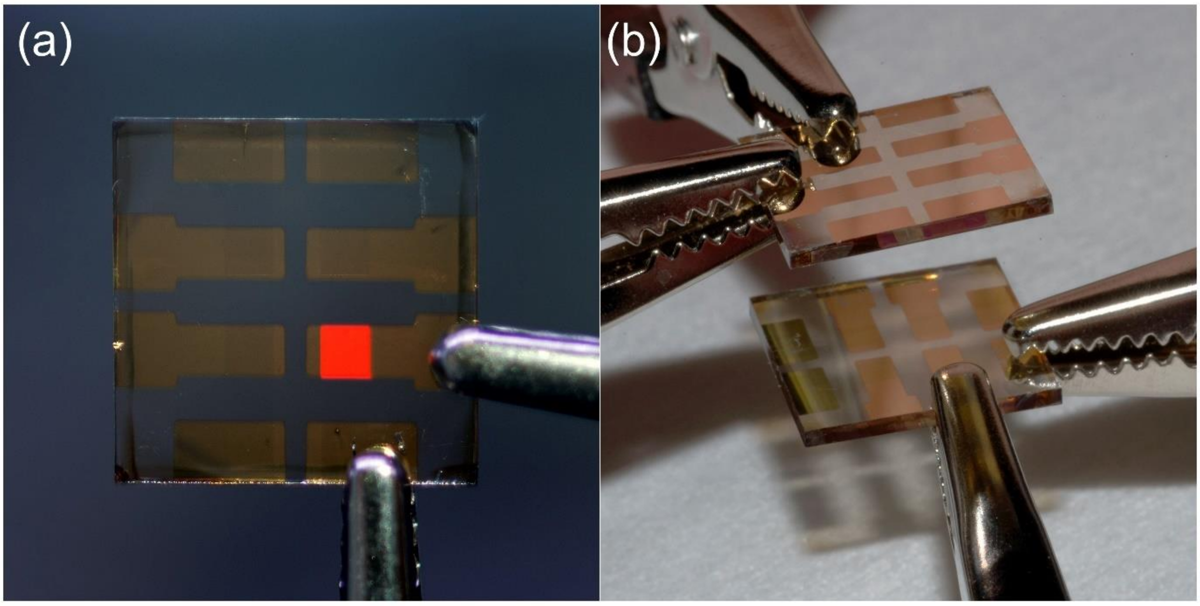

Supplementary Figure 33. Digital photos of luminescence and detection. (a) Photograph of a device excited at 3 V. Note that the color displayed here is not the main wavelength but the peak tail. **(b)** Device twins were combined into a light-emitting & self-driven detection system. The spacing of the two devices is about 1 cm in the light-emitting & self-driven detection system.

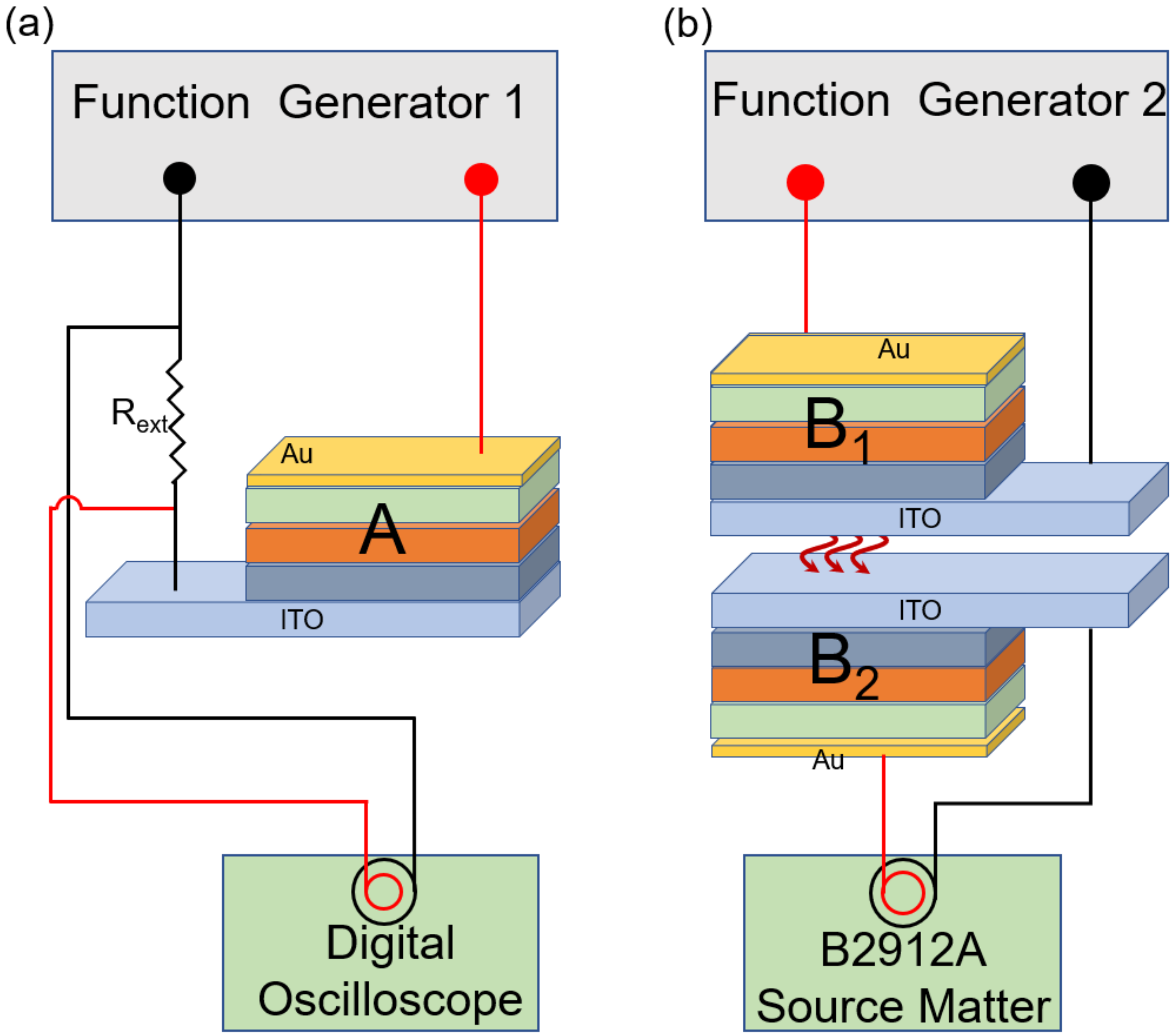

Supplementary Figure 34. Schematic diagram of photoelectric response measurements. (a) The electrical response and **(b)** spectral response tests.

The left part is a schematic of the electrical response of a device. The current versus time of the device under rectangular voltage conditions was examined by a digital oscilloscope, and the results are shown in **Fig. 4h.** The spectral response system consisted of two identical devices, one of which acted as a light source, with a constant voltage of 2.2 V provided by a source meter; and the other device served as NIR light detection at zero bias, enabling self-driven detection. We considered the first device to be of the same nanosecond order as the previous current response from the application of bias to the generation of light.

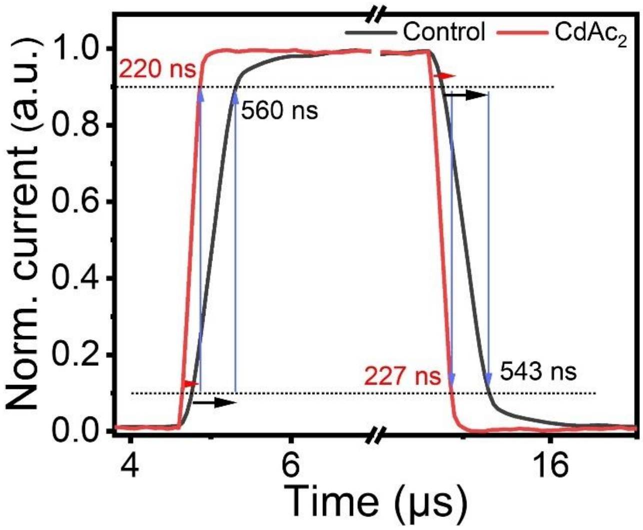

Supplementary Figure 35. The electrical response characteristics of the two representative devices in a single cycle. The rise time of electrical response reduced from 560 ns for the control one to 220 ns for the $CdAc_2$-added device, and the fall time reduced from 543 to 227 ns.

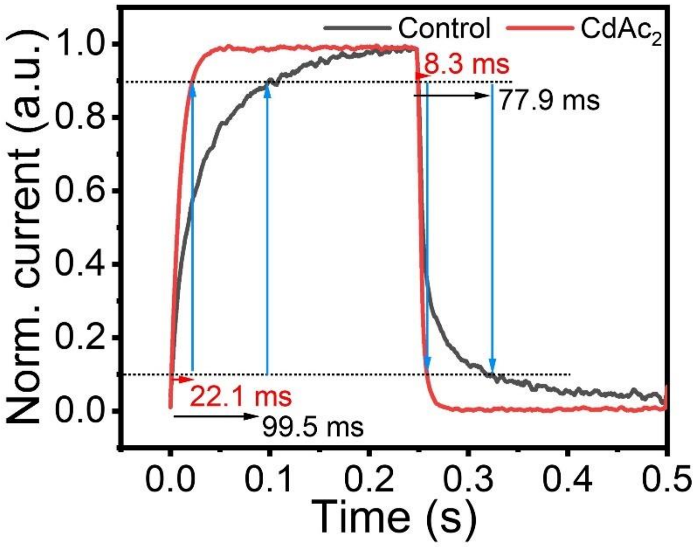

Supplementary Figure 36. The light response of the self-driven near-infrared detection in a single cycle. The light-response rise time reduced from 99.5 ms for the control one to 22.1 ms for the $CdAc_2$-added device, and the fall time reduced from 77.9 to 8.3 ms.

Supplementary Table 1. Photophysical parameters extracted from various optical measurements of the perovskite films.

	A_1	τ_1 (μs)	A_2	τ_1 (μs)	τ_{av}	PLQE (%)	k_{rad} ($\times 10^6$ s^{-1})	k_{nonrad} ($\times 10^6$ s^{-1})	k_{rad}/k_{nonrad}
Control	0.4	0.2	0.1	0.9	0.5	69	1.38	0.62	2.23
CdI_2-added	0.7	0.3	0.2	1.2	0.8	83	1.04	0.21	4.95
$PbAc_2$-added	0.6	0.4	0.2	1.6	1.2	74	0.62	0.21	2.95
**$CdAc_2$-added**	**0.6**	**0.5**	**0.3**	**2.7**	**2.1**	**79**	**0.38**	**0.10**	**3.8**

Supplementary Table 2. Crystal data and structure refinement.

Empirical formula	$C_2N_4H_{10}CdI_4$	
Formula weight	710.14	
Temperature	296.1(5) K	
Wavelength	1.54184 Å	
Crystal system	Monoclinic	
Space group	P 1 21/c 1	
Unit cell dimensions	a = 11.52020(10) Å	a= 90°.
	b = 10.95930(10) Å	b= 108.2090(10)°.
	c = 12.70800(10) Å	g = 90°.
Volume	1524.08(2) $Å^3$	
Z	4	
Density (calculated)	3.095 Mg/m^3	
Absorption coefficient	74.846 mm^{-1}	
F(000)	1240	
Crystal size	0.08 x 0.06 x 0.04 mm^3	
Theta range for data collection	4.040 to 64.991°.	
Index ranges	-13<=h<=13, -12<=k<=12, -14<=l<=14	
Reflections collected	29869	
Independent reflections	2591 [R(int) = 0.1044]	
Completeness to theta = 64.991°	99.9 %	
Absorption correction	Semi-empirical from equivalents	
Max. and min. transmission	1.00000 and 0.11590	
Refinement method	Full-matrix least-squares on F^2	
Data / restraints / parameters	2591 / 28 / 101	
Goodness-of-fit on F^2	1.076	
Final R indices [I>2sigma(I)]	R1 = 0.0726, wR2 = 0.1973	
R indices (all data)	R1 = 0.0744, wR2 = 0.1996	
Extinction coefficient	0.00097(12)	
Largest diff. peak and hole	2.512 and -2.987 e.$Å^{-3}$	

Supplementary Table 3. The fitted formation time constants (τ_{et}) and first-order decays (τ_1) of each GSBs for transient absorption (TA) spectra.

sample	T	GSB_1 (389 nm)	GSB_2 (467 nm)	GSB_3 (507 nm)	GSB_α (780 nm)
control	τ_1 (fs)	242	173	647	-
	τ_{et} (fs)	-	-	-	952
**CdAc2 added**	τ_1 (fs)	**149**	**151**	**571**	**-**
	τ_{et} (fs)	**-**	**-**	**-**	**769**

Here, the kinetics are fitted by a four-exponential function of $\Delta A(t) = A_1exp(-t/\tau_1) + A_2exp(-t/\tau_2) + A_3exp(-t/\tau_3) – C_1exp(-t/\tau_{et}) + A_0$, where the A_1, A_2, A_3 and C_1 are amplitudes, and the τ_1, τ_2 and τ_3 are decay time constants and τ_{et} is the rising component. Only τ_1 and τ_{et} are listed due to the complex slow decay process of τ_2 and τ_3 are attributed to trap-assisted recombination and Auger recombination.

Supplementary Table 4. Electrophysical parameters of the devices with various PMs.

	Additive molar ratio (%)	EQE (max) (%)	Peak radiance ($W \cdot sr^{-1} \cdot m^{-2}$)
**Control**	**0**	**19.39**	**46.4**
**CdI_2-added**	5	19.89	26.2
	**10**	**20.57**	**28.1**
	15	18.69	14.1
	20	14.45	7.1
**$PbAc_2$-added**	2.5	20.6	46.05
	**5**	**21.39**	**50.64**
	10	18.89	33.99
	15	15.40	25.25
**$CdAc_2$-added**	1	22.69	50.49
	**2.5**	**24.11**	**50.47**
	5	21.80	38.05
	10	18.79	33.12

Supplementary Table 5. The performance of the unencapsulated devices with different phase compositions.

Emitter	Fabrication	EQE_{max} (%)	Operational stability (h) (Without encapsulation)
3D	Stoichiometric FAI +150°C annealing	0.93	1.51
$3D+CdAc_2$		2.21	2.88
1D/3D	Stoichiometric FAI +105°C annealing	1.81	3.67
$1D/3D+CdAc_2$		4.2	6.75
2D/3D	excess FAI +150°C annealing	8.9	16.8
$2D/3D+CdAc_2$		12.2	32.9
1D/2D/3D	excess FAI +105°C annealing	19.6	22.6
$1D/2D/3D+CdAc_2$		24.1	39.1

Note that the devices compared in the table are unencapsulated.

Supplementary Table 6. Performance comparison without an additional light out-coupling structure of near-infrared PeLEDs reported in this work and literature.

Perovskite	Additive	EQE_{max} (%)	$EQE_{average}$ (%)	λ_{EL} (nm)	FWHM (nm)	Operating stability	Reference
$FAPbI_3$	5AVA	20.7	19.2	803	Not mentioned	T_{50} = 20 h (I = 100 mA·cm^{-2})	*Nature* 562, 249–253 (2018)
$FAPbI_3$	5AVA	20.2	17.4	799	41	T_{80} = 20h (I = 57 mA·cm^{-2})	*Nature Photonics* 14, 215–218 (2020)
$FAPbI_3$	AEAA	22.2	19.4	800	Not mentioned	T_{50} = 18.6 h (I = 100 mA·cm^{-2})	*Nature Communications* 12, 5081 (2021)
NMA-$FAPbI_3$	Poly-HEMA	20.1	Not mentioned	800	55	T_{50} = 46 h (I = 0.1 mA/cm^{2})	*Nature Photonics* 12, 783–789 (2018)
$FAPbI_3$	ODEA	21.6	19	800	Not mentioned	T_{50} = 1.5h (I = 25 mA·cm^{-2})	*Nature Photonics* 13, 418–424 (2019)
$FAPbI_3$	NMAI	20.2	17.5	variable	46	Not mentioned	*Light: Science & Applications* 9, 89 (2020)
$FAPbI_3$	ODEA+PEAI	22.8	Not mentioned	Around 800	Not mentioned	T_{50} ≈ 2.1h (I = 100 mA·cm^{-2})	*Matter,* 2021, 4(11): 3710-3724
$FA_xCs_yPbI_3$	$Pb(SCN)_2$ +5AVAI	21.2	Not mentioned	787	39	T_{50} ≈ 20 h (I = 100 mA·cm^{-2})	*ACS Appl Mater Interfaces* , 13(24):28546-28554. (2021)
$FAPbI_3$	SFB10	Around 16	Not mentioned	803	Not mentioned	T_{50} ≈ 2000 h (I = 10 mA·cm^{-2})	*Nature Photonics*, 2022, 16(9): 637-643
**$FAPbI_3$**	**5AVA+ $CdAc_2$**	**24.1**	**22.4**	**804**	**46**	**T_{50} = 110.7h (I = 10 mA·cm^{-2})**	**This work**
	**5AVA+$PbAc_2$ +FA_2CdI_4 inserting layer**	**23.3**	-			**T_{50} = 174.3h (I = 10 mA·cm^{-2})**	

Supplementary Table 7. Response time comparison of perovskite devices reported in this work and literature.

Perovskite	electrical response time	light response rise time (ms)	light response fall time (ms)	Detection wavelength (nm)	Reference
$MAPbI_3$	～s	-	-	-	ACS Applied Materials & Interfaces, 2021, 13(30): 35617-35624.
$(FASnI_3)_{0.6}(MAPbI_3)_{0.4}$	-	32	19	810	Advanced Materials Technologies, 2020, 5(1): 1900752.
$MAPbI_3$	-	0.032	0.02	870	Light: Science & Applications, 2020, 9(1): 1-8.
$MAPbI_3$	-	0.2533	0.2304	780	Materials Today Physics, 2021, 18: 100398.
$MAPbI_3$	-	<200	<200	800	Advanced Materials, 2018, 30(21): 1706986.
$MAPbI_3$	-	6.7	4.4	780	Chemical Engineering Journal, 2021, 406: 126779.
Polymer/CsPbBr3	-	2.6	9.6	808	Advanced Functional Materials, 2019, 29(48): 1906756.
$FA_{0.5}MA_{0.5}Pb_{0.4}Sn_{0.6}I_3$	-	～ms	～ms	940	Nature communications, 2022, 13(1): 1-8.
CdS/ $MAPbI_3$	-	0.54	2.21	700	Advanced Materials, 2019, 31(12): 1806725
$(Cs_{0.06}FA_{0.79}MA_{0.15})Pb(I_{0.85}Br_{0.15})_3$ +BNA	-	49	27	785	Small, 2020, 16(24): 2001417
CdS/ $MAPbI_3$	-	3.2	9.6	730	ACS applied materials & interfaces, 2019, 11(43): 40204-40213
$FAPbI_3$	～ms	-	-	800	Nature Communications, 2021, 12(1): 1-8.
**$FAPbI_3$**	**～200 ns**	**23.6**	**8.3**	**804**	**This work**

Supplementary References

1. Ma D, *et al.* An Effective Strategy of Combining Surface Passivation and Secondary Grain Growth for Highly Efficient and Stable Perovskite Solar Cells. *Small* **17**, e2100678 (2021).
2. Yuan Z, *et al.* Unveiling the synergistic effect of precursor stoichiometry and interfacial reactions for perovskite light-emitting diodes. *Nat Commun* **10**, 2818 (2019).
3. Xu W, *et al.* Rational molecular passivation for high-performance perovskite light-emitting diodes. *Nature Photonics* **13**, 418-424 (2019).
4. Jia Y, *et al.* Excess Ion-Induced Efficiency Roll-Off in High-Efficiency Perovskite Light-Emitting Diodes. *ACS Appl Mater Interfaces* **13**, 28546-28554 (2021).
