# Supplementary material for: Synergistic passivation and stepped-dimensional perovskite analogs enable high-efficiency near-infrared light-emitting diodes
Source: Nat Commun. 2022 Dec 2;13:7425. doi: 10.1038/s41467-022-35218-0 (PMC9718757; doi:10.1038/s41467-022-35218-0)
Supplement: Supplementary file 2 — Description of Additional Supplementary Files [file 41467_2022_35218_MOESM2_ESM.pdf]

## Description of additional Supplementary File

**File Name: Supplementary Data 1**

Description: $FA_2CdI_4$ crystal structure results of the single-crystal X-ray crystallography analyses, which has been published on Crystallography Open Database (Cambridge Crystallographic Data Centre, DOI: 10.5517/ccdc.csd.cc2dhc09, CCDC Number: 2219290).
